# Gouda Cheese with Modified Content of β-Casein as a Source of Peptides with ACE- and DPP-IV-Inhibiting Bioactivity: A Study Based on In Silico and In Vitro Protocol

**DOI:** 10.3390/ijms22062949

**Published:** 2021-03-14

**Authors:** Anna Iwaniak, Damir Mogut, Piotr Minkiewicz, Justyna Żulewska, Małgorzata Darewicz

**Affiliations:** 1 Chair of Food Biochemistry, Faculty of Food Science, University of Warmia and Mazury in Olsztyn, Pl. Cieszyński 1, 10-726 Olsztyn-Kortowo, Poland; damir.mogut@uwm.edu.pl (D.M.); minkiew@uwm.edu.pl (P.M.); darewicz@uwm.edu.pl (M.D.); 2 Department of Dairy Science and Quality Management, Faculty of Food Science, University of Warmia and Mazury in Olsztyn, Oczapowskiego 7, 10-719 Olsztyn-Kortowo, Poland; justyna.zulewska@uwm.edu.pl

**Keywords:** bioinformatics, BIOPEP-UWM database, bioactive peptides, β-casein, Gouda cheese

## Abstract

In silico and in vitro methods were used to analyze ACE- and DPP-IV-inhibiting potential of Gouda cheese with a modified content of β-casein. Firstly, the BIOPEP-UWM database was used to predict the presence of ACE and DPP-IV inhibitors in casein sequences. Then, the following Gouda cheeses were produced: with decreased, increased, and normative content of β-casein after 1 and 60 days of ripening each (six variants in total). Finally, determination of the ACE/DPP-IV-inhibitory activity and the identification of peptides in respective Gouda-derived water-soluble extracts were carried out. The identification analyses were supported with in silico calculations, i.e., heatmaps and quantitative parameters. All Gouda variants exhibited comparable ACE inhibition, whereas DPP-IV inhibition was more diversified among the samples. The samples derived from Gouda with the increased content of β-casein (both stages of ripening) had the highest DPP-IV-inhibiting potency compared to the same samples measured for ACE inhibition. Regardless of the results concerning ACE and DPP-IV inhibition among the cheese samples, the heatmap showed that the latter bioactivity was predominant in all Gouda variants, presumably because it was based on the qualitative approach (i.e., peptide presence in the sample). Our heatmap did not include the bioactivity of a single peptide as well as its quantity in the sample. In turn, the quantitative parameters showed that the best sources of ACE/DPP-IV inhibitors were all Gouda-derived extracts obtained after 60 days of the ripening. Although our protocol was efficient in showing some regularities among Gouda cheese variants, in vivo studies are recommended for more extensive investigations of this subject.

## 1. Introduction

According to Mahdi et al [1], food proteins attract the attention of the scientists and food manufacturers due to their nutritional value and health-beneficial properties. The latter stem from, for example, the presence of amino acids and peptides exhibiting biological functions (i.e., bioactive peptides), which are released from proteins when hydrolyzed [1]. Such peptides may exert various bioactivities such as, e.g., antidiabetic [2], antihypertensive, opioid, antioxidative, immunomodulating, or antibacterial [3]. The first two biological functions of peptides are involved in reducing blood pressure and glucose levels, respectively [4]. The ability of peptides to lower the blood pressure is related to the inhibition of angiotensin-converting enzyme (ACE; EC 3.4.15.1). Briefly, ACE catalyzes the conversion of angiotensin I into vasoconstricting angiotensin II and induces the release of aldosterone. It is also involved in the hydrolysis of bradykinin (a potent vasodilator). Thus, the involvement of the peptide inhibitor in the reaction leads to the reduction of blood pressure [5]. In turn, dipeptidyl peptidase IV (DPP IV; EC 3.4.14.5) inactivates incretin hormones (incretins), such as glucagon-like peptide 1 (GLP-1) and glucose-dependent insulinotropic polypeptide (GIP). They are responsible for enhancing meal-induced insulin secretion and contribute to glucose homeostasis [2]. According to Raikos [2], incretins may also affect the suppression of glucagon release, delay gastric emptying, and modulate appetite. Hence, the action of the DPP-IV inhibitor plays an important role in extending the half-life and maintaining the concentrations of active incretins. The antidiabetic function of peptides may additionally result from the inhibition of α-glucosidase (EC 3.2.1.20) and α-amylase (EC 3.2.1.1).

Peptides with ACE- and DPP-IV-inhibiting activities have been identified in milk and dairy products, including ripened cheeses [6,7]. For example, some DPP-IV inhibitors were identified in water-soluble extracts of Gouda cheese. Peptide LPQNIPPL matched β-casein, whereas LPQ matched α_S1_-, α_S2_-, and β-casein [7]. In turn, two peptides known from their antihypertensive effect, IPP and VPP, were identified in 36 cheeses of Swiss origin [6]. Additionally, the effects of peptides with antidiabetic and antihypertensive activities were the subject of meta-analyses [8,9]. Gao et al [8] demonstrated that the consumption of 30 g of cheese per day decreased the relative risk for type 2 diabetes development. Other studies showed that blood pressure reduction was associated with the presence of ACE inhibitory peptides in Gamalost (a cheese) consumed by the Norwegian population [9].

The analysis of peptides derived from food proteins can be supported by the bioinformatic-assisted (in silico) approach, which is based on the use of databases of bioactive compounds (i.e., peptides) as well as programs helping evaluate and compare food proteins as potential sources of bioactive peptides [10]. One of the first in silico studies undertaken by Iwaniak and Dziuba [11] concerned the comparison of animal and plant proteins (97 sequences in total) as the potential sources of ACE inhibitors. Using the BIOPEP-UWM database (formerly known as BIOPEP) of bioactive peptide sequences as well as a mathematical parameter called “A”, meaning the frequency of the occurrence of bioactive fragment(s) in a protein chain, β-casein was found to be the best source of ACE-inhibiting peptides among all proteins analyzed. Similar results were observed when comparing different food protein sequences as the potential sources of DPP-IV inhibitors using the InterPro program as an additional bioinformatic analysis [12]. Ever since, more information on the new peptidic ACE- and DPP-IV inhibitors was published and downloaded into the BIOPEP-UWM database [13]. It may be expected that it could improve the previous results. 

According to Sulieman et al [14], Gouda cheese is considered one of “the most prominent cheeses on the planet, representing 50 to 60 percent of the world’s cheese utilization”. Combining two facts, namely that (i) this ripened cheese is known from its bioactive properties resulting from the presence of peptides (see above) and (ii) β-casein was a relatively good source of ACE- and DPP-IV-inhibiting sequences (based on in silico analysis), this study aimed to find out if the modified content of β-casein would affect the above-mentioned bioactivities of Gouda cheese.

## 2. Results and Discussion

### 2.1. Profiles of the ACE- and DPP-IV-Inhibitory Activity of Casein Sequences—An In Silico Analysis

The simplified profiles of ACE- and DPP-IV-inhibiting activity of the bovine casein sequences are shown in Table 1.

They include the total number of fragments with the above bioactivities that were potentially encrypted in caseins. These numbers include the fragments which were repeated in the casein chain. Moreover, the profiles present the number of such peptides categorized by the length of the chain. The individual amino acid sequences of peptides exhibiting ACE- and DPP-IV-inhibiting activity are present in Table S1 (Supplementary Materials). In silico analyses of the peptide profiles of casein sequences showed that the number of ACE inhibitors matching all proteins analyzed ranged from 80 to 129, which referred to κ- (genetic variant A) and β-casein (genetic variant B), respectively. In turn, the number of DPP-IV-inhibiting peptides ranged from 114 (α_S1_-casein, genetic variant A) to 170 (β-casein, genetic variant E). Additionally, all casein sequences analyzed were potentially the best sources of di- and tripeptide ACE/DPP-IV inhibitors. Several factors may affect the results of in silico analyses. One of them is the number of peptides with a specific activity present in the database [10]. Currently, the BIOPEP-UWM database contains information over 4000 peptides with several dozens of bioactivities. Among them, 1016 and 421 are ACE and DPP-IV inhibitors, respectively. They represent the most abundant activities of peptides present in the BIOPEP-UWM, which affects the probability of exact matching of the protein sequences. Hence, the continuous update of the databases with the sequences of biopeptides is postulated to increase the chance to discover new sequences in food-derived protein sources [15]. Another aspect related to the abundancy of peptides in a protein chain is their length. The shorter the peptide chain, the greater the chance for the exact match to the protein sequence [16]. This rule explains the dominant presence of shorter motifs with ACE- and DPP-IV-inhibiting bioactivity in all caseins analyzed. The third aspect includes the impact of a protein structure on peptides matching it. Our complementary analysis carried out using the ProtParam tool [17,18] allowed calculating the percentages of amino acids present in the casein sequences. The idea of such research relied on the rule “the higher the number of specific amino acid residues in a protein, the better the probability of finding a peptide in it”. The ProtParam analysis revealed that the most abundant amino acids occurring in all genetic variants of α_S1_-caseins were: Glu (11.6–12.1%), Leu (7.5–10.3%), Pro (7.9–9.1%), Ser (7.5–8.6%), and Lys (7.0–7.5%). In the case of all genetic variants of β-caseins analyzed, the ranking of the first 5 major amino acids was as follows: Pro (15.8–16.7%), Leu (10.5–11.0%), Gln (9.6–10.5%), Val (9.1%; all variants), and Glu (8.1–9.1%). The predominant residues occurring in α_S2_-casein (one variant analyzed) were: Lys (11.3%), Glu (10.8%), Ser (7.7%), Gln (7.2%), and Leu (7.2%), whereas in κ-casein (one variant analyzed) these were: Pro (11.1%), Tre (8.9%), Ala (8.4%), Gln (7.9%), and Ser (7.4%). It is well known that the amino acid composition (i.e., structure) of a peptide affects its biological activity [19]. According to the QSAR (i.e., Quantitative Structure-Activity Relationships) approach, the ideal peptidic ACE inhibitor should be composed of N-terminal Ala, Leu, Ile or Val, and C-terminal Pro [20,21]. The amino acid composition of the casein sequences indicates that all of them were rich in Val, Ala, Leu, and Pro (see Table S1). Moreover, the highest number of ACE inhibitors containing these amino acids (127) was observed in the genetic variant B of β-casein. Considering the regularities between the structure and the DPP-IV-inhibiting activity of peptides, it was observed that the majority of these peptides contained N-terminal W and C-terminal Pro [22]. According to Pissurlenkar et al [23], the presence of proline in a peptide is crucial for peptide activity because it acts with Phe and Tyr, which are the part of the active site of the enzyme. Looking at the casein sequences analyzed using the ProtParam program, it was found that the genetic variant B of β-casein was the richest source of Pro (data not shown). Finally, the results of in silico analyses were the premise to create “the ranking” of the casein sequences as the potential sources of ACE- and DPP-IV-inhibiting peptides (see Table 2).

This ranking included the presence of peptides with the ACE/DPP-IV-inhibitory effect which are found in the BIOPEP-UWM database. The appropriate reference information concerning their bioactivity is provided in BIOPEP-UWM (see the link called “references”). The main criterion of the ranking was the total number of peptide fragments with the above bioactivities found in their casein precursors (the higher the number, the better the protein is). The comparison of all casein sequences revealed that all β-casein variants taken for the in silico analyses were the best sources of ACE and DPP-IV inhibitors. Such a conclusion encouraged us to continue the studies under experimental conditions.

### 2.2. Changes in Peptide Profiles of Different Variants of Gouda Cheese Based on RP-HPLC Separation

The RP-HPLC separation of the samples derived from Gouda cheese variants followed the calculation of β-casein content in milks used to produce different variants of cheeses as well as the compositional analysis of cheeses. The data reflects the composition of cheeses after 60 days of ripening.

β-Casein content was understood as the ratio of α_s_-casein to β-casein and the percentage of β-casein in all casein fractions of milks used to produce different variants of Gouda cheese. The results are shown in Table 3.

Casein accounts for approximately 80% of bovine milk proteins. The principal casein fractions are α_s1_-, α_s2_-, β-, and κ-casein, and their relative proportion is estimated at 45.0, 12.0, 35.0, and 8.0%, respectively [24]. Our protocol applied to produce Gouda with increased β-casein content showed that milk used for that purpose had higher percentages of β-CN than other milks. It should be noted that the α_s_-CN to β-CN ratio is a better indicator of changes in the composition of the casein fractions in milk. Creamer et al [25] reported that κ-casein was also prone, though to a lesser extent, to dissociation from the casein micelles when milk is cooled. Therefore, the changes in the κ-casein content will affect the percentage of β-casein in all casein fractions. However, the increase in β-CN content was not as high as expected. The ultrafiltration process applied to concentration β-CN removed from RMF50+PUF50 and then RMF7+PUF50 did not result in a significantly (*p* ≥ 0.05) higher β-CN content in milk (the mixture of PRUF, RMF50 and cream), which could be caused by the deposition of β-CN on the membrane surface. However, further studies are required to better understand this phenomenon.

The composition of Gouda cheese variants after 60 days of ripening is shown in Table 4. Our results concerning the moisture as well as fat contents were similar to data obtained by Jo et al [26] for Gouda cheeses aged for less than 3 months. No significant differences (*p* ≥ 0.05) were observed in the fat content among the different variants of cheeses. The assumed fat content in dry matter (d.m.) was 45%, and our experimental cheeses showed 46.99, 46.74, and 45.24% fat contents (data not shown) for Gouda with reduced, normative, and increased content of β-CN, respectively. The protein content ranged from 26.51 to 27.28%, which are typical values for this type of cheese ([27], p. 170). No significant difference (*p* ≥ 0.05) was detected in the ash content. However, significant differences were demonstrated for the calcium (Ca) content (data not shown), with the lowest value found for Gouda cheese with reduced β-CN content (approx. 8160 mg × kg^−1^) as well as 8995 and 9339 mg Ca per kg of cheese reported for Gouda cheese with normative and increased contents of β-CN, respectively. The differences in the ash content may reflect the treatment of milks subjected to the cheese making process. Calcium in milk exists partly in a soluble form and partly in an insoluble or colloidal form associated with casein ([28], p. 248). About two thirds of calcium is colloidal and bound to casein micelles [29]. Thus, the concentration of colloidal calcium is strongly correlated with the casein content of milk. During the MF process, calcium bound to a casein micelle will be retained by the membrane unless the process is carried out at a low temperature, resulting in the loss of the micellar structure. When the MF process is carried out at refrigerated temperatures (below 10 °C), additional factors play a role in calcium migration between the soluble and the colloidal phase, and thus through the membrane [30]. The micelles are adversely affected by low temperature, at which the β-CN chains start to dissociate and calcium hydroxyphosphate leaves the micelle structure, where it existed in colloidal form, and migrates into the solution [31].

According to the scientific reports, the changes between the proportions of the casein fractions in the ripening cheeses are rather not a new issue [32]. For example, St-Gelais and Haché [33] added powdered β-casein to the newly produced cheese, thereby increasing its content in the product. The modified product was characterized with the higher contents of moisture calcium compared with the control cheese [33]. Generally, any changes in the amount of α-CN or β-CN would modify the properties of milk and cheese. αs-Casein (α-CN) and β-casein (β-CN) are the basic microstructural constituents of cheese. In their study on the coagulating properties of milk, Storry et al [34] observed that rennet clotting was related to the proportions of α-CN and β-CN present in milk. Yun et al [35] demonstrated that the curd tension value increased significantly when milk was fortified with β-CN. These results suggest that β-casein might be essential for curd hardening. Similar results were reported by other authors [36,37]. Van Hekken and Holsinger [32] studied gelling properties of milk gels enriched with β-casein and have shown that the β-casein-enriched fractions, when treated with glucono-δ-lactone and rennet, formed softer gels that had greater syneresis and lower water holding capacities than skim milk gels. They have suggested that milk with a modified α-CN-to-β-CN ratio may be used as the starting material in the production of novel cheeses due to unique gelling properties.

The modified protein profiles may also influence flavor and texture development as cheeses age. Proteolysis is probably the most important biochemical event during the ripening of most cheese varieties, with a major impact on flavor and texture [38,39,40]. To date, it has not yet been fully characterized in any cheese variety but considerable progress has been made for Cheddar ([28], pp. 180 and 410). During Cheddar cheese ripening, αs_1_-casein is completely hydrolyzed within 3–4 months. Although β-casein is readily hydrolyzed by chymosin in solutions, in cheeses it is very resistant to chymosin but slowly hydrolyzed (approximately 50% within 6 months) by plasmin [28,39]. Proteolysis has been well characterized for Cheddar cheese and, generally, similar results apply to other low-cooked, internally bacterially-ripened cheeses (e.g., Dutch types) [41].

It is also well documented in the literature that casein is a rich source of biopeptides in comparison to the other food protein sources [42]. Especially, β-casein is considered the fraction with a high potential regarding its bioactivity resulting from the presence of bioactive peptides. For example, one of its fragments (position 60–70 of the chain) is called a strategic zone due to the presence of peptides with ACE-inhibitory, immunostimulatory, and opioid effects [43]. The abundance of β-casein in biopeptides was also observed using in silico analyses, according to which this sequence was potentially the best source of ACE-inhibiting, DPP-IV-inhibiting, and antithrombotic peptides as well as peptides regulating the action of the stomach mucosal membrane [12]. Taking into account that our in silico ranking indicated β-casein as the theoretically the best source of ACE and DPP-IV inhibitors (among the other fractions of this protein; see Table 2) and considering the differences in the percentages of total peak areas demonstrated for each cheese variant sample using the RP-HPLC-UV/Vis method, it was a “natural process” to try to answer the question: “are there any changes in peptide profiles of water-soluble extracts of Gouda cheese related to β-casein content and ripening duration?”. The RP-HPLC-MS/MS of all samples derived from Gouda cheese as well as the experimental assay of their ACE- and DPP-IV-inhibiting effects were conducted to answer this question.

**Figure 1:**
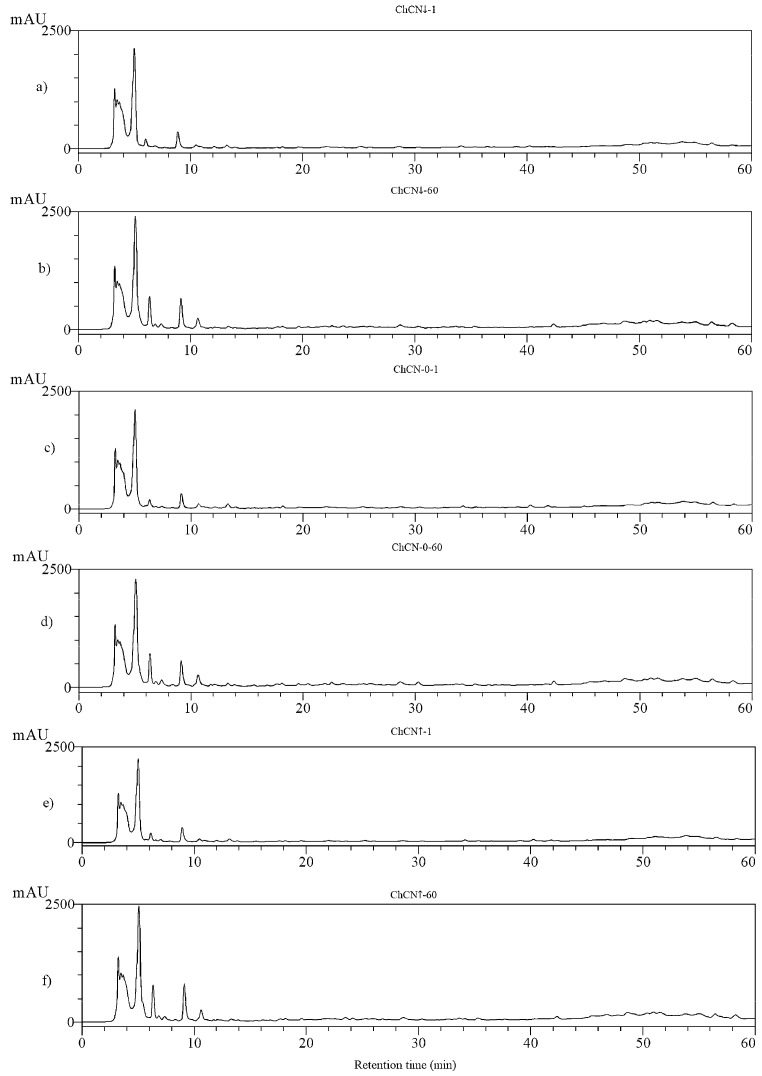
RP-HPLC chromatograms of water-soluble extracts derived from Gouda cheese with modified β-casein content before and after ripening (1 and 60 days, respectively). Abbreviations: ChCN↓-1 (**a**), ChCN↓-60 (**b**), ChCN-0-1 (**c**), ChCN-0-60 (**d**), ChCN↑-1 (**e**), ChCN↑-60 (**f**)—water-soluble extracts derived from Gouda cheese with: reduced (↓), normative (0), and increased (↑) content of β-casein after 1 (suffix 1) and 60 (suffix 60) days of ripening, respectively (see Methods).

Water-soluble extracts derived from three variants of Gouda cheese including the beginning and the end of the ripening process (1st and 60th day, respectively) were the subject of RP-HPLC separation to observe the possible changes that might occur in their peptide profiles. This method, based on the comparison of percentages of peak areas, was successfully applied to monitor the hydrolysis of milk, carp, and herring proteins [16,44]. The chromatograms presenting the separation of the six variants of samples (i.e., ChCN-0-1, ChCN-0-60, ChCN↑-1, ChCN↑-60, ChCN↓-1, and ChCN↓-60) are shown in Figure 1a–f. According to Iwaniak et al [16], the chromatograms of proteolysis products, obtained via the protocol used also in this work, can be divided into three time interval [min] segments, namely: 0.00–13.99; 14.00–40.00, and 40.01–60.00. In the previous work, the first time interval was typical for all chromatograms and contained the highest peaks eluting between 5.00 and 13.99 min, not taken into further interpretation of results because they were injection peaks corresponding to the non-retained substances like, e.g., buffer components, as well as to low-molecular-weight compounds present in the separated samples [44]. In this experiment, the peaks eluted between 3 and 6 min had a similar shape and contained probably the same components in all samples (Figure 1 in the main text and Figure S1 in the Supplementary Materials). These peaks probably contain the components of solvents, such as Bis-TRIS, urea, used for dissolving of the samples as well as TFA used for pH adjustment (see Section 3.7). Differences between chromatograms of samples form cheeses with different ripening times were observed at retention times longer than 6 min (Figure 1 in the main text, Figures S1 and S2 in the Supplementary Materials).

The time intervals between 60.01 and 80.00 min were not taken into account in analyses as recommended previously [16]. In this experiment, the peaks eluted between 6 and 14 min were specific for the ripening time. Especially the height and area of peaks eluted at 6–7 min and 10–11 min increased during cheese ripening. The most noticeable changes in all chromatograms were found between 14.00 and 40.00 min of RP-HPLC separations (Figure 1 in the main text and Figure S2 in the Supplementary Materials). The peaks and groups of peaks, markedly greater after 60 days, were eluted within 17–19 min, 28–29 min, and 32–34 min. The time interval between 40 and 60 min also encompassed peaks, which were larger after 60 days than after 1 day of ripening (42–52 min and 56–59 min). This meant that as the Gouda cheese aged, new peaks occurred at the cost of a decreasing number of peaks appearing at the beginning of the ripening process. Gupta et al [45] made similar observations applying RP-HPLC to analyze water-soluble extracts of Cheddar cheese for the ACE-inhibitory activity. The largest number of peaks was produced by a heterogenous mixture of proteolysis products in UF permeates of water-soluble extracts of Cheddar cheeses [45]. We observed changes in the height and area of peaks with the same retention time at cheese chromatograms after 1 and 60 days. The changes in peaks with the same retention time reflect changes in the composition of peptide mixtures [46]. The changes in the peptide profiles of Gouda cheese-derived samples were also observed when analyzing the differences between the percentages of total peak areas of these two time intervals (Table 5).

The percentage peak areas within particular time intervals did not show any regularity. The fraction of cheese with a low β-casein content after 1 day revealed the highest percentage of the fraction with retention times between 0 and 14 min and the lowest content of the fraction with retention times between 14.01 and 40 min.

The explanation of this observation requires a brief description of enzymes involved in cheese ripening. According to the literature, cheese proteolysis is a complex process resulting from the activity of residual coagulant (rennet), indigenous milk proteases, and starter culture enzymes [47]. The first listed above is the major source of proteolytic enzymes (usually chymosin; EC 3.4.23.4). The specificity of chymosin against casein fractions is well known. Apart from Phe_105_-Met_106_ bond located in κ-casein, it also cleaves several bonds in β-, α_s1_-, and α_s2_-caseins, leading to the production of peptides. According to the scientific reports, α_s2_-casein is more resistant to the action of chymosin than α_s1_-casein [48].

As regards indigenous milk proteases, the major one is plasmin (EC 3.4.21.7). It is specific toward peptide bonds Lys-X and Arg-X (X stands for any amino acid). Apart from κ-casein, which is resistant to the action of plasmin, the order of hydrolysis of caseins by this enzyme is as follows: β-casein ≈ α_s2_-casein > α_s1_-casein [48]. According to McSweeney [48], the most important casein fraction for plasmin action in cheese is β-casein. Its hydrolysis produces the following fragments of β-casein: 1–28, 29–105, 29–107, 1–105, 1–109, 29–209, 106–209, and 108–209 [48]. β-Casein proteolysis by plasmin leads to, e.g., the release of strongly hydrophobic C-terminal fragments with retention times longer than this of the entire protein [49]. The increased area of the peaks eluted between 55 and 59 min in all cheese samples after 60 days may be partially attributed to plasmin action.

Starter culture enzymes possess complex proteolytic systems that are crucial in cheese ripening. When thinking about their action on specific casein, the role of lactocepins needs to be elucidated. Lactocepin is a major proteinase of *Lactococcus*. Its role is to produce short peptides from caseins to let the lactococcal cell grow in milk. In cheese, lactocepins are involved in the further degradation of casein-derived intermediate-size peptides produced using chymosin or plasmin. Studies concerning lactocepins derived from different *Lactococcus* strains led to their classification as P_I_- and P_III_-type proteinases. The first acts rapidly on β-casein and slowly on α_s1_- and κ-caseins, whereas the second one acts the opposite way [48].

To recapitulate, when generally thinking about cheese proteolysis, the mechanism of this enzyme-involving process follows a similar pattern, i.e., the primary action of chymosin (residual coagulant) on κ-casein and participation of chymosin, plasmin as well as microbial enzymes in the ripening process. The “enzyme pattern” of ripening also looks similar. The leading role is ascribed to chymosin hydrolysis of α_s1_-casein (Phe_23_-Phe_24_ bond; except for cheeses cooked at high temperatures). As regards the rate of hydrolysis, α_s1_-casein is hydrolyzed faster than β-casein in many cheese varieties. On the contrary, β-casein is hydrolyzed faster than α_s1_-casein in Swiss-type cheeses, including a concomitant increase in γ-casein, which indicates the impacts of plasmin and denaturation of chymosin during cooking. To summarize, depending on cheese type, its cooking temperature and microflora involved in its ripening affect its proteolysis rate and its peptide profile [48]. According to the literature, Cheddar is the best-characterized cheese considering the factors discussed above [50]. To the best of our knowledge, no such research was undertaken to analyze Gouda. When looking at the results obtained (see Table 5), it is highly likely that β-casein was hydrolyzed faster than other caseins at the beginning of the ripening process due to the plasmin action.

Comparison of the samples “ascribed” to the ripening stage led to observe that, regardless the β-casein content, the similarity between peptide profiles measured by the peak area percentages in both time intervals was more distinctive for the samples derived after 1 day of cheese maturation. In turn, the area of peaks eluted within 14.00–40.00 min and 40.01–60.00 min, for all three cheese variants after 60 days of maturation, was almost equal (see Table 3). This shows that the peptide-protein profiles of individual cheese variants became more similar as the Gouda ripening process continued.

To recapitulate, the differences observed in the peak area percentages among all samples indicated some changes in the peptide profiles of water-soluble extracts derived from Gouda cheese with modified β-casein content before and after the ripening. This observation was the premise for the next step of the study aimed to determine the ACE- and DPP-IV-inhibitory bioactivities of the water-soluble cheese-derived extracts and (depending on the results) identify peptides in the samples.

### 2.3. Identification of ACE- and DPP-IV Inhibitors in Water-Soluble-Extracts of Different Gouda Cheese Variants

The results showing the ACE- and DPP-IV-inhibiting potential of Gouda cheese water-soluble extracts with the modified content of β-casein are shown in Table 6.

All peptidic extracts showed both activities. When considering the ACE-inhibitory effect, there were practically no differences between cheese variants and their ripening stage. The IC_50_ values of ACE inhibition were nearly identical for three samples derived from Gouda with decreased and normative contents of β-casein. The IC_50_ value determined for the first two samples (ChCN↓-1 and ChCN↓-60) was 14.840 mg x mL^−1^, whereas for the last one (ChCN-0-1) it was 14.860 mg × mL^−1^. The highest ACE-inhibitory activity was observed for the ChCN-0-60 sample (IC_50_ = 14.590 mg × mL^−1^). No statistical differences were observed between ChCN-0-1 and ChCN-0-60 samples. Neither the concentration of β-casein nor the ripening stage had a statistically significant change in the ACE inhibiting activity.

More diversified results were obtained for the DPP-IV-inhibitory activity of the Gouda water-soluble extracts. The highest DPP-IV-inhibitory activity was observed for both ChCN↑ samples. Their IC_50_ values were nearly identical (IC_50_ = 9.174 and 9.171 mg × mL^−1^; see Table 6). Identical IC_50_ (18.760 mg × mL^−1^) values were also found for the water-soluble extracts derived from Gouda cheese with a normative β-casein content. The DPP-IV-inhibitory activity of the extracts derived from the cheese with the reduced β-casein content was comparable; however, it slightly increased for the sample obtained after 60 days of ripening (IC_50_ = 18.300 mg × mL^−1^). Nevertheless, this activity was comparable with that of the ChCN↓-1 sample (IC_50_ = 17.990 mg × mL^−1^). To recapitulate, the increase in β-casein content in Gouda cheese had a statistically significant impact on the DPP-IV-inhibitory activity of extracts, but there were no statistically significant changes in the IC_50_ values between day 1 and 60, which suggests that the ripening process did not affect bioactivity. Furthermore, there were no statistically significant changes in the bioactivity between the control and ChCN↓ samples, excluding the ripening stage. Finally, the confirmed ACE- and DPP-IV-inhibitory activity of the extracts derived from all three variants of Gouda cheese and two stages of ripening was the premise for identifying peptides showing the above effects.

Based on the profiles of ACE- and DPP-IV-inhibitory activity of casein fractions, we found that 356 (in total) peptide fragments showing these effects were encrypted in their protein precursors (see Table S1). The RP-HPLC-MS/MS analysis of the water-soluble extracts derived from 6 variants of Gouda cheese allowed identifying 63 peptides (see Table S2; Supplementary Materials). Among them, 11 peptides were known as DPP-IV inhibitors, 48 were ACE inhibitors, and 4 exhibited both activities. Regardless of β-casein content and ripening stage of Gouda cheese, 26 peptides were identified in all its variants. Some sequences (14) were found in ChCN↓-, ChCN↑-, and ChCN-0-type cheeses but after 1 day of ripening. In turn, 20 peptides that were not identified at the beginning of the ripening process appeared in the cheese after 60 days. Two ACE inhibitors, namely KDERF and LKKISQ, were typical of the ChCN↑-1 and ChCN-0-1 samples.

Diversified peptidic characteristics of all Gouda cheese variants is a complex issue to explain. According to Garbowska et al [51], the presence of peptides in cheeses depends on the balance between their synthesis and degradation by the proteolytic system throughout the ripening process. Peptidolytic activity is related to the ripening process as well as type and culture conditions of adjunct starters [51]. According to Garbowska et al [51], the ripening process enhanced the ACE-inhibitory activity of the cheese to a certain level. Once the cheese had reached the optimal affinage, its bioactivity decreased. This phenomenon was explained by successive action of LAB (i.e., lactic acid bacteria) proteases leading to the further degradation of bioactive fragments and, hence, their inactivation [51].

**Figure 2:**
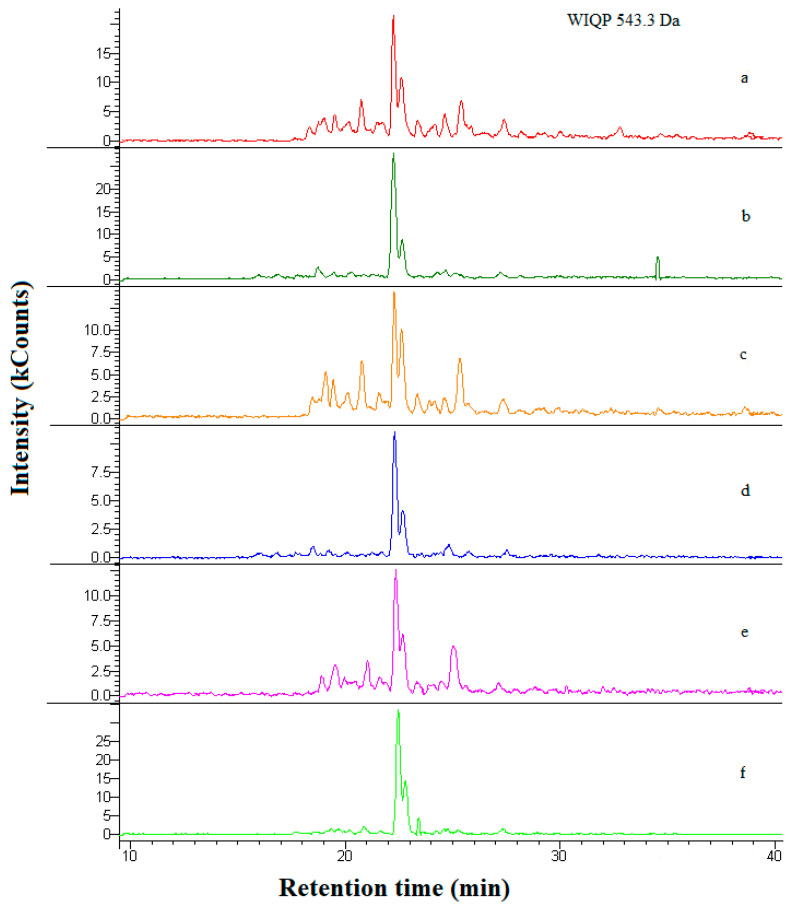
An example of an RP-HPLC-MS/MS chromatogram of the WIQP peptide acting as a DPP-IV inhibitor identified in all Gouda cheese variants. Samples derived from: ChCN↓-1 (**a**), ChCN↓-60 (**b**), ChCN-0-1 (**c**), ChCN-0-60 (**d**), ChCN↑-1 (**e**), ChCN↑-60 (**f**), respectively. Abbreviations: ChCN↓-1, ChCN↓-60, ChCN-0-1, ChCN-0-60, ChCN↑-1, ChCN↑-60—water-soluble extracts derived from Gouda cheese with: reduced (↓), normative (0), and increased (↑) content of β-casein after 1 (suffix 1) and 60 (suffix 60) days of ripening, respectively.

An example of the chromatogram of the identified peptides is presented in Figure 2. It shows the WIQP sequence acting as a DPP-IV inhibitor. According to the results of an in silico analysis, this sequence was encrypted in αs_2_-casein (genetic variant A; fragment 208–211). The WIQP peptide was identified in Gouda cheese, regardless of its β-casein content and ripening stage. The *m/z* of the (M+H)^+^ precursor ion of WIQP was 543.3 Da, and six intensive peaks referring to the individual cheese water-soluble extracts were observed in the 23rd min (t_R_ = 22.460 min).

**Figure 3:**
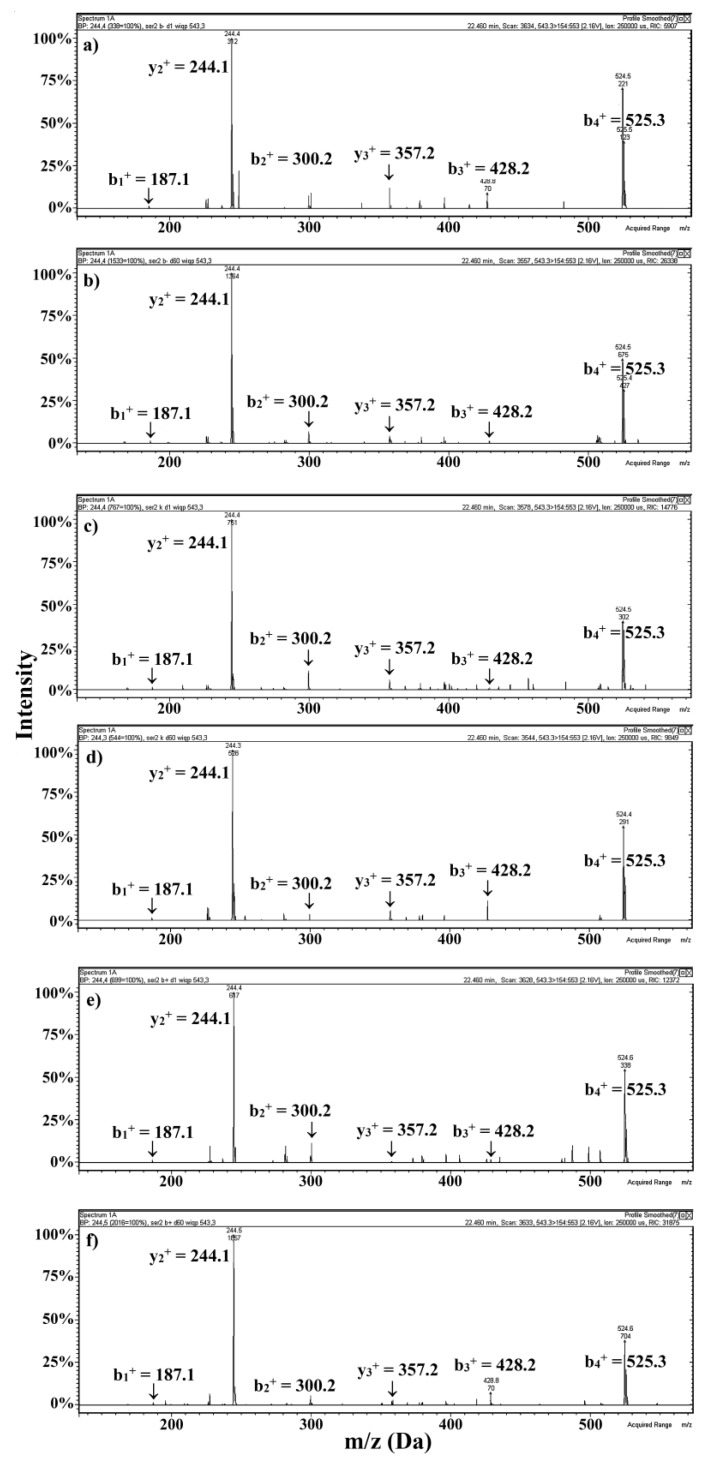
MS/MS spectra of the WIQP DPP-IV inhibitory peptide identified in the water-soluble extracts of the Gouda cheese. Nomenclature of daughter ions according to Roepstorff and Fohlman [52]. Samples derived from: ChCN↓-1 (**a**), ChCN↓-60 (**b**), ChCN-0-1 (**c**), ChCN-0-60 (**d**), ChCN↑-1 (**e**), ChCN↑-60 (**f**), respectively. Abbreviations: ChCN↓-1, ChCN↓-60, ChCN-0-1, ChCN-0-60, ChCN↑-1, ChCN↑-60—water-soluble extracts derived from Gouda cheese with: reduced (↓), normative (0), and increased (↑) content of β-casein after 1 (suffix 1) and 60 (suffix 60) days of ripening, respectively.

Spectra of the WIQP peptide from all cheese samples analyzed are presented in Figure 3. B and Y ions allowing peptide identification were consistent with these predicted theoretically, with the precision achievable by the mass spectrometer applied. Exemplary spectra of all peptides are presented in the Supplementary Materials. The method used enables the identification of short peptides (2–5 amino acid residues) when information concerning proteolytic enzyme specificity is missing. Proteomic software is often unable to detect such peptides using a low resolution ion trap mass spectrometer.

Moreover, we tried to analyze this peptide employing the fragmentomic approach, which assumes that shorter fragments with known bioactivity that are encrypted in a peptide of interest may affect the function of the whole sequence [53]. This concept of peptide research was successfully applied in our previous works [16,54] to study bitter-tasting motif occurring in milk and soybean hydrolysates, and allowed us to reveal that WIQP contained the following motifs with the known effects: IQP and QP (ACE/DPP-IV inhibitors), IQ and WI (DPP-IV inhibitors). However, none of these sequences were identified in cheese samples.

### 2.4. Results of Experimental Data Analysis

**Figure 4:**
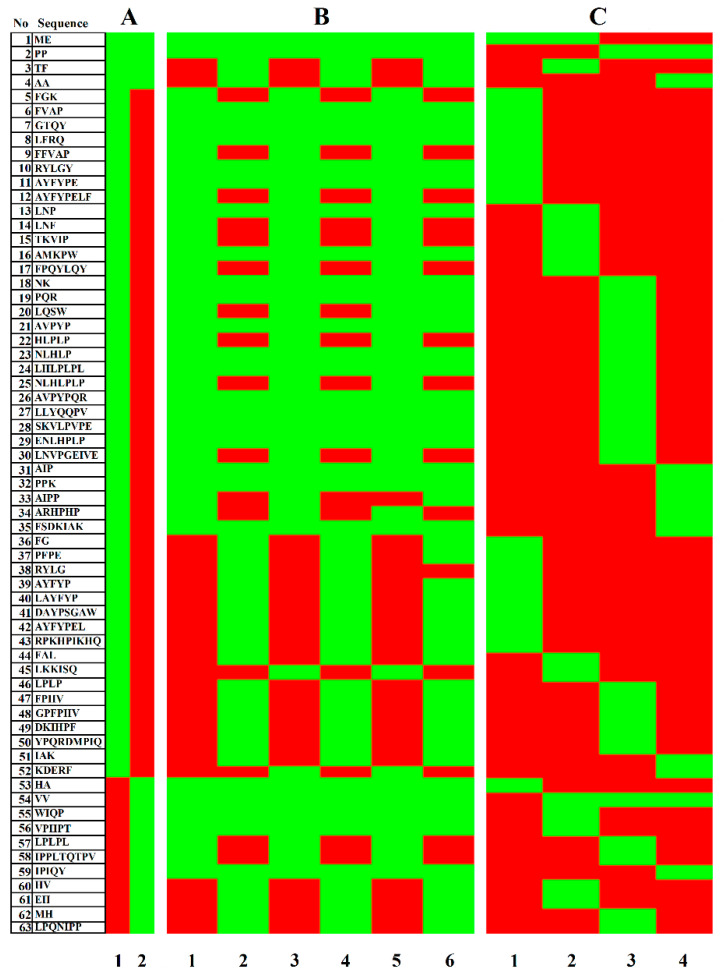
Heatmap of ACE and DPP-IV inhibitors identified in Gouda cheese samples. Figure prepared using the Heatmapper program [55,56]. Map (**A**) (activity): ACE inhibition (A1), DPP-IV inhibition (A2); map (**B**) (peptide presence in a cheese sample): ChCN↓-1 (B1), ChCN↓-60 (B2), ChCN-0-1 (B3), ChCN-0-60 (B4), ChCN↑-1 (B5), ChCN↑-60 (B6); map (**C**) (peptide presence in casein): α_S1_-casein (C1), α_S2_-casein (C2), β-casein (C3), κ-casein (C4); colors: green—“yes”, red—“no”. ChCN↓-1, ChCN↓-60, ChCN-0-1, ChCN-0-60, ChCN↑-1, ChCN↑-60—water-soluble extracts derived from Gouda cheese with: reduced (↓), normative (0), and increased (↑) content of β-casein after 1 (suffix 1) and 60 (suffix 60) days of ripening, respectively.

The next step was to create the heatmaps (see Figure 4) visualizing the results concerning the presence of ACE- and DPP-IV inhibitors in all Gouda cheese variants. Figure 4A–C includes three heatmaps.

The heatmap A shows that the ACE-inhibitory activity was predominant. There were much fewer DPP-IV inhibitors and barely a few peptides showing both bioactivities. The heatmap B shows that although the peptide profiles of the analyzed cheeses were similar regardless of the casein content, they were differentiated by the ripening period (heatmap B). Some peptides (TF and AA; dual bioactivity) were absent in the cheese at the beginning of ripening (see lines 1, 3, and 5) but appeared at its final stage (see lines 2, 4, and 6). Some peptides were observed to disappear during the ripening (e.g., FGK and FFVAP; ACE inhibitors). According to Santiago-López et al [57], peptides are produced during the ripening due to the action of plasmin and LAB-derived enzymes to be subsequently hydrolyzed or accumulate during storage. Hence, peptides observed on the first day of cheese ripening, but not detected after 60 days, might have been the substrates for hydrolysis or other reactions during cheese ripening. Finally, the heatmap C indicates that β-casein was the best source of ACE and DPP-IV inhibitors. Thus, the latter map was the premise to calculate the following parameters: the frequency of released fragments with the ACE- or DPP-IV-inhibitory activity during cheese ripening (A_Eexp._), and the relative frequency of release of fragments with these bioactivities during cheese maturation (W_exp._). The above parameters correspond to A_E_ and W parameters, designed to describe the predicted efficiency of proteolysis [58]. The results of calculations are provided in Table 7.

The A_Eexp._ and W_exp._ parameters indicate the changes taking place in individual proteins during cheese ripening. The highest values of these parameters, referring to the number of ACE/DPP-IV inhibitors identified in a casein source, are marked in bold in Table 7 said the data presented in the Table show that the sample derived from the cheese with the increased content of β-casein after 60 days of ripening was the best source of ACE inhibitors. The parent source of 14 identified peptides was the genetic variant B of β-casein. Considering the W_exp._ values (W_exp_. = 0.122; the highest one), it can be concluded that the ripening process also affected the release of ACE inhibitors that were matching the sequence of α_s1_-casein (genetic variant D). The value above was achieved for the cheese samples derived after 60 days of ripening but regardless of β-casein content. Values of A_Eexp._ were identical for the above cheese samples (A_Eexp._ = 0.061). According to these results, it can be concluded that all variants of Gouda cheese obtained after 60 days of ripening were the best precursors of peptides with the ACE-inhibitory activity.

Considering the DPP-IV-inhibitory activity of cheese variants, the number of identified peptides ranged from two to seven sequences. Depending on A_Eexp._ and W_exp_. values, the cheese samples obtained after 60 days of the ripening were the best sources of DPP-IV inhibitors, regardless of β-casein content in the cheese variant. The peptides in these cheese variants matched β-casein (genetic variant B) and αs_2_-casein (genetic variant A). To recapitulate, similarly to the ACE-inhibiting potential of the samples analyzed, the cheese variants after 60 days of ripening were the best sources of DPP-IV inhibitors.

The results of ACE and DPP-IV inhibitors identification in Gouda water-soluble extracts using the heatmap and quantitative parameters (A_Eexp._, and W_exp._) enabled showing more clearly some regularities than the results obtained under laboratory conditions. For example, the determination of the bioactivity of all cheese samples showed their ACE- and DPP-IV-inhibitory potential in vitro. The ChCN↑-1 and ChCN↑-60 samples exhibited a stronger DPP-IV-inhibiting effect than the analogical samples measured for their ACE-inhibitory potential. This observation might contradict with the conclusion that the ACE-inhibitory activity was predominant in all samples (see heatmap A, Figure 4). To explain it briefly, in silico analyses applied in our study show which bioactive peptides were predominant. However, these analyses take no account of, e.g., the IC_50_ values of individual peptides and numbers of peptides identified under laboratory conditions. Another aspect of the hybrid analysis combining in silico and in vitro protocols [10] is the discrepancy between the numbers of peptides identified using these protocols. In our in silico study, it was possible to find 356 biopeptides showing ACE- and/or DPP-IV-inhibiting effects that were matching particular sequences of caseins. This number includes the repetitions (if possible) of a single biopeptide in a protein sequence. Peptide identification in the Gouda cheese water-soluble extracts using RP-HPLC-MS/MS led to confirm the presence of 63 peptides in the samples. As mentioned above, this identification enables confirming the presence of a peptide in a sample but does not show its quantity as well as the number of repetitions. The issues concerning the differences between the in silico and in vitro results of the biopeptide analysis were discussed in detail in our previous works [16,54].

To recapitulate, regardless of the β-casein content, Gouda cheese variants showed ACE-/DPP-IV-inhibitory activity. However, the results obtained fail to strictly answer the question of whether β-casein content modification enhanced or diminished the ACE-/DPP-IV-inhibitory activity of Gouda cheese. Nevertheless, our results might be the premise for the further in vivo analysis of modified Gouda cheese. Iwaniak and Mogut [59] summarized data on metabolic syndrome-preventive peptides identified in different cheese types. Loads of information concerned the bioactivity of cheeses determined in vitro. It is well-known that some peptides show the effect in vitro, which was not observed in vivo [60]. Thus, the conclusion made by Garbowska et al [51] that the in vitro ACE-inhibitory activity cannot be used as the sole criterion in the evaluation of potentially-hypotensive substances may refer to any bioactivity determined in the food matrix in vitro, including ripening cheeses.

## 3. Materials and Methods

### 3.1. Reagents

Angiotensin-converting enzyme (ACE; 1 UN; cat no. A6778), dipeptidyl peptidase IV (DPP-IV, 10 units/mg protein, cat no. D7052), hippuryl-histidyl-leucine (HHL; cat no. H1635), trifluoroacetic acid (TFA; cat no. T6508), TRIS (hydroxymethyl)aminomethane hydrochloride (TRIS-HCl; cat no. 93313), ethyl acetate (cat. no. 270989), phosphate buffer pH 7.4 (cat no. P3813), Gly-Pro-*p*-nitroanilide (Gly-Pro-*p*-NA; cat no. G2901), borate buffer (cat no. 82634), sodium carbonate (cat no. S7795), sodium dodecyl sulfate (SDS; cat. No. L3771), and urea (cat no. U5378) were purchased from Sigma-Aldrich Sp. z o.o. (Poznań, Poland). Bis-TRIS, i.e., 2,2-bis(hydroxymethyl)-2,2′,2″-nitrilotriethanol (cat no. B9754) and acetonitrile (ACN; cat no. 102644151) were purchased at ABChem (Olsztyn, Poland) and 12% polyacrylamide gel was acquired from Bio-Rad Laboratories Inc., Hercules, CA, USA). Nylon membrane filters (Whatman^®^, 0.2 μm pore size, cat no. WHA7402004) were purchased from Sigma-Aldrich Sp. z o.o. (Poznań, Poland) and Munktell-Filtrak 390 grade filters (cat. no. 8.012.120.900) from EQUIMED (Olsztyn, Poland). All other chemicals used in the experiments were of analytical grade. Water used to formulate solutions and buffers was prepared using a Milli-Q PLUS system (Millipore Corp., New York, NY, USA).

### 3.2. In Silico Analysis

The bioinformatic analysis (in silico) was employed to predict the presence of ACE- and DPP-IV inhibitors in all casein sequences provided in the BIOPEP-UWM database [13] available at http://www.uwm.edu.pl/biochemia/index.php/pl/biopep ([61]; accessed: 11 January 2020). Thus, the 13 following sequences were selected for the analysis: α_S1_—genetic variants A (186/ID 1086), B (199/ID 1087), C (199/ID 1088), and D (214/ID 1089); α_S2_—genetic variant A (222/ID 1090); β—genetic variants: A1 (209/ID 1097), A2 (209/ID 1098), A3 (209/ID 1099), B (209/ID 1100), C (209/ID 1101), E (209/ID 1102), and F (209/ID 1103); κ—genetic variant A (190/ID 1117). The numbers in brackets refer to the number of an amino acid residue in a sequence and the accession number of a sequence in the BIOPEP-UWM database, respectively. The presence of ACE- and DPP-IV-inhibiting peptides in casein sequences was predicted using the function called “Profiles of potential biological activity”. According to Minkiewicz et al [13], the profile of the potential biological activity of protein is defined as the type and the location of peptide with a specific activity in a protein chain. This computation was run using to the following protocol available in the BIOPEP-UWM database: BIOPEP-UWM → Proteins → Analysis → Profiles of potential biological activity → Select activity → Protein database. The option “Select activity” required selecting “ACE inhibitor” and “dipeptidyl peptidase IV inhibitor” keywords available when developing the toolbar. The keywords above are provided in exact words as they can be found in the BIOPEP-UWM database. The last stage before starting the computations, i.e., clicking the “Protein database” bar, was the selection of the particular protein ID (see above). The analyses were carried out in January–March 2020.

### 3.3. Production of Gouda Cheese and Modification of Its β-Casein Content

Gouda cheese was produced on a semi-industrial scale at the University’s Dairy Research and Development Center (Department of Dairy Science and Quality Management, University of Warmia and Mazury in Olsztyn, Poland). Raw milk (about 800 kg) was collected from the University of Warmia and Mazury (UWM, Poland) Experimental Station in Bałdy and transported to the University’s Dairy Research and Development Center (Department of Dairy Science and Quality Management, University of Warmia and Mazury in Olsztyn). Then, raw milk was separated in the dairy technological hall at 45 °C using a Model LWG20 Centrifuge (Spomasz, Gniezno, Poland). The raw skim milk (about 770 kg) was thermized (65 °C; 20 s) using a plate heat exchanger Model P20-HB (Alfa Laval, Lund, Sweden). Then, the thermized skim milk was microfiltered (MF) at 50 °C with a 3X concentration factor (CF) using a pilot-scale microfilter system (TAMI Industries, Nyons, France) equipped with 0.1 µm nominal pore diameter ceramic graded permeability (GP) Membralox membranes (model EP1940GL0.1μAGP1020, Alumina, Pall Corp., East Hills, NY, USA). The membranes in a tubular single stainless steel module consisted of 3 ceramic tubes, 19 channels each with a 4 mm channel diameter, which resulted in 0.72 m^2^ surface area. The microfiltration process resulted in retentate (RMF50—microfiltration retentate produced at 50 °C) and permeate (PMF50—microfiltration permeate produced at 50 °C). Then, the skim milk (150 L) and PMF50 (approx. 270 L) were mixed and subjected to ultrafiltration (UF) at 50 °C with 5X CF to produce retentate (RUF50—ultrafiltration retentate produced at 50 °C) and permeate (PUF50—ultrafiltration permeate produced at 50 °C) using a pilot-scale UF system equipped with a polyethersulfone spiral-wound (SW) membrane (model 3838 HFK-131, NYV/T Spacer (31 mil), Koch Membrane Systems, Inc. Wilmington, MA, USA; nominal separation cutoff: 10,000 Da). The RMF50 and PUF50 were mixed, stored at 2 °C for 24 h, and then microfiltered at 7 °C with 3X CF using a pilot-scale MF system equipped with a fluoropolymer SW membrane (model FSM 0.15PP-3838/48P, Alfa Laval, Lund, Sweden; nominal pore size: 0.15 µm). The produced streams were retentate (RMF7—microfiltration retentate produced at 7 °C) and permeate (PMF7—microfiltration permeate produced at 7 °C). The final RMF7 was diluted with PUF50 to bring it back to the original volume and diafiltered (DF) using the same SW membrane as described above. The conditions were as follows: temperature 7 °C and 3X CF. The retentate and permeate produced were RDF7 (diafiltration retentate produced at 7 °C) and PDF7 (diafiltration permeate produced at 7 °C). The PMF7 and PDF7 permeates were mixed and ultrafiltered at 50 °C to produce retentate (PRUF—ultrafiltration retentate from MF/DF permeates) with a desired protein content to be used in the production of cheese with an increased content of β-casein. A total of three variants of cheeses were produced: control (i.e., normative), with low and high β-casein content. Pasteurized (72 °C/15 s) standardized milk was used to produce the normative cheese. The cheese with a low β-casein content was produced from RDF7 and PUF. In turn, RMF50 and PRUF were used to produce the high-β-casein cheese. Pasteurized (95 °C/3 min) cream was used for fat standardization to obtain a fat content in cheese milk at approx. 3.4% (depending on the protein content). The projected fat content in dry matter of cheese was 45%. The cheese manufacture protocol was typical of Dutch-type cheese (Technological instruction no. 330/88, Central Association of Dairy Cooperatives), i.e., Gouda, and its typical steps included: the addition of CaCl_2_ (0.02%, P.P.H. “STANLAB” Sp.J., Lublin, Poland), culture (CHN19, Chr. Hansen, Hoersholm, Denmark) and rennet (Chymax M 1000, Chr. Hansen) at 32 °C; cutting semi-firm curd after approx. 30–40 min; mixing; whey drainage; water addition; heating up to 38 °C; mixing; molding; pressing; salting; ripening. 

Finally, three variants of Gouda cheese, i.e., with normative, increased, and reduced content of β-casein (CN), were abbreviated as: ChCN-0, ChCN↑, and ChCN↓, respectively. Increased and reduced contents of β-casein should be understood as its respective higher/lower contents compared to the content of this protein fraction in the “normative” cheese.

After completed salting (day one) and 60-day ripening (12 °C), cheese samples were taken immediately for composition analysis. The fat content was determined using the method of Schmid-Bondzyński-Ratzlaff [62]. Cheese moisture was determined gravimetrically by drying 2.0 g of cheese in a forced-air oven at 100 °C for 24 h ([63], methods: 33.2.44 and 990.20). The ash content was determined by incinerating the dried residue of 3.0 g of cheese at a temperature of ≤ 550 °C ([63], methods: 33.7.07 and 935.42). The Kjeldahl method (1.0 g of cheese) was used to determine total nitrogen content (TN) ([63] methods: 2001.14 and 33.7.12A), and the protein content was calculated as TN × 6.38. All analyses were performed in duplicate.

The SDS-PAGE electrophoresis was used to determine the relative protein proportions (band %) according to the protocol of Zulewska et al [64]. The change in the content of β-casein in milks used to produce Gouda cheeses was expressed as the ratio of α_s_-casein to β-casein and the percentage of β-casein of all casein fractions of milks used for Gouda production (detected on SDS-PAGE gels, i.e., α_s_-CN, β-Cn, κ-CN bands) [64].

### 3.4. Water-Soluble Extracts from Gouda Cheese

Each Gouda cheese variant after 1 and 60 days of ripening was subjected to the extraction of water-soluble extracts according to the method of Pritchard et al [65] who produced such extracts from Cheddar cheese.

Firstly, 100 g of grated cheese was mixed with 300 mL of distilled water and then homogenized using a blender (Waring, Snijders Scientific Tilburg, The Netherlands). The slurry was moved to the test tubes, gently stirred, and incubated for 1 h (40 °C, 100 rpm) using a Unimax 1010 thermoincubator (Heidolph Instruments GmbH & CO. KG, Germany). Then, the sample was centrifuged for 40 min (4000 rpm) using a centrifuge (Hermle Labortechnik GmbH, Wehingen, Germany). After the pellet removal, the supernatant was recentrifuged at the same conditions as above. The newly formed supernatant was filtered using the Munktell & Filtrak 390 filter. The incubation conditions of the slurry, like time and temperature (among the others), were firstly introduced by Kuchroo and Fox [66] as a recommendation for the preparation of water-soluble cheese extracts. According to this recommendation, it was possible to achieve the maximum yield of extracted material [66]. Finally, the filtrate was freeze-dried at −70 °C. Finally, six variants of water-soluble extracts were obtained: ChCN-0-1, ChCN-0-60, ChCN↑-1, ChCN↑-60, ChCN↓-1, and ChCN↓-60. The abbreviations mean the origin of Gouda cheese extract sample and the suffix “1” or “60” describes the day of cheese ripening. For example, the symbol “ChCN↑-1” should be read as the water-soluble peptide extract derived from Gouda cheese (Ch) with increased content of β-casein (CN↑-) after the 1st day of cheese ripening (1).

### 3.5. ACE Inhibition by Water-Soluble Gouda Cheese Extracts

The potential of water-soluble peptide cheese extracts to inhibit ACE was measured using the method introduced by Cushman and Cheung [67]. Shortly, ACE bioactivity assessment involves the spectrophotometric monitoring (at λ = 228 nm) of the amount of hippuric acid (HA) released from hippuryl-histydyl-leucine (HHL). Firstly, the water-soluble peptide extract originating from the individual cheese variant was dissolved in 0.1 M borate buffer, pH = 8.3, to the desired concentrations. Then, 20 μL of the water-soluble peptide cheese extracts were combined with 100 μL of the substrate (5 mM HHL dissolved in 0.1 M borate buffer, pH = 8.3), and the mixture was incubated (37°C, 30 min). To start the enzymatic reaction, 20 μL of ACE (4 mU) was added, and the incubation was continued at the same temperature and time conditions. The reaction was terminated by adding 250 μL of 1.0 M HCl. The released HA was eluted using 1.5 mL of ethyl acetate, and then the mixture was centrifuged using a Z 233 M-2Microlitre Centrifuge (Hermle Labortechnik, Germany) (10 min, 3000 g). Next, 1 mL of the upper layer was moved to the test tube and evaporated. Then, 1 mL of distilled water was added, and after 10 min the absorbance (λ = 228 nm) was measured using a UV-Visible spectrophotometer (Spectronic GENESYS 6, Thermo Electron Corporation, USA). The percentage of ACE inhibition was calculated using the following formula:

ACE inhibition (%) = [(A_control_ − A_inhibitor_)/(A_control_ − A_blank_)] × 100 (1)

where:

- A_inhibitor_—absorbance of the sample containing the potential inhibitor (peptide cheese extract);
- A_control_—absorbance of the sample containing water instead of the potential inhibitor;
- A_blank_—absorbance of the sample containing water instead of the enzyme solution and the potential inhibitor. The assay was carried out in triplicate.

According to the enzyme specification, one unit produces 1.0 μM of HA from HHL per one min at 37 °C and pH = 8.3.

### 3.6. DPP-IV Inhibition by Water-Soluble Gouda Cheese Extracts

The ability of water-soluble Gouda cheese extracts to inhibit DPP-IV was determined using the method applied by Lacroix and Li-Chan [68] Briefly, the activity of DPP-IV can be monitored spectrophotometrically at λ = 405 nm by observing the amount of *p*-nitroaniline (*p*-NA) released from a chromogenic substrate, i.e., glycyl-prolyl-*p*-nitroanilide (Gly-Pro-*p*-NA). Thus, the water-soluble extracts (the sample) derived from the individual cheese variant were dissolved in 100 mM TRIS-HCl, pH = 8.0, to the desired concentrations. Then, 25 μL of the sample was combined with 25 μL of the substrate (12 mM Gly-Pro-p-NA dissolved in 100 mM TRIS-HCl, pH = 8.0), and the mixture was pre-incubated (37 °C, 10 min). The reaction was initiated by adding 50 μL DPP-IV (0.02 unit × mL^−1^) dissolved in 100 mM TRIS-HCl, pH = 8.0) and continued for 30 min (37 °C). The reaction was stopped by adding 100 μL of 1 M sodium acetate (pH = 4.0). The absorbance (A) of the released *p*-NA was measured at 405 nm using a UV-Visible spectrophotometer (Spectronic GENESYS 6, Thermo Electron Corporation, USA). The percentage of DPP-IV inhibition was calculated using the following formula:

DPP-IV inhibition (%) = [(A_control_ − A_inhibitor_)/(A_control_ − A_blank_)] × 100 (2)

where:

- A_inhibitor_—absorbance of the sample containing the potential inhibitor (peptide cheese extract);
- A_control_—absorbance of the sample containing TRIS-HCl instead of the potential inhibitor;
- A_blank_—absorbance of the sample containing TRIS-HCl instead of the enzyme solution and the potential inhibitor. The assay was carried out in triplicate.

According to the enzyme specification provided by the supplier, one unit of DPP-IV was defined as the concentration of the enzyme that produces 1 μM of *p*-NA from Gly-Pro-*p*-NA per one minute at 37 °C and pH = 8.0.

The percentages of ACE and DPP-IV inhibition of the samples (i.e., peptide cheese extracts) were used to calculate the IC_50_ values (mg × mL^−1^), describing the sample concentrations corresponding to their 50% inhibition. These values were calculated using GraphPad Prism 5.02 for Windows^®^ [69]. The computations made automatically included standard error (at 95% confidence interval). Calculations were carried out using the program option “inhibition (log) vs. normalized response—variable slope” available in the tab called “Dose-response curves—Inhibition” being the part of the panel called “Nonlinear regression” [69,70]. To determine the IC_50_ values, the assay concentrations of the samples ranged from 2.5–25.0 mg × mL^−1^ (both bioactivities; see above methods). The concentration ranges of 10.0–30.0 mg × mL^−1^ were applied by several authors [71,72,73] to measure the ACE-inhibitory activity of water -soluble cheese extracts. We started from the lowest concentration (2.5 mg × mL^−1^) to get at least five separate concentrations for IC_50_ calculations, followed by the guidelines concerning EC_50_/IC_50_ estimation [74].

Khan and Kumar [75] successfully applied such a number of sample concentrations to create the “concentration—inhibition” plot to calculate the IC_50_ describing the ACE-inhibitory effect of medicinal plants.

### 3.7. Reversed-Phase High Performance Liquid Chromatography with a UV/Vis Detector (RP-HPLC-UV/Vis) Analysis of Peptidic Profiles of Water-Soluble Gouda Cheese Extracts

The changes in the peptidic profiles of the water-soluble extracts of Gouda cheese (six variants, see above) were analyzed using the reversed-phase high performance liquid chromatography (RP-HPLC) with a UV/Vis detector of Shimadzu^®^ system (Tokyo, Japan). This system comprised of: a CBM-20A controller, a DGU-20A5 degasser, an SIL-20AC HT autosampler, two LC-20AD pumps, a CTO-10AS VP thermostat, and an SPD-M20A photodiode detector. The separation was done on a Jupiter Proteo Phenomenex^®^ column (Torrance, CA, USA) with the following parameters: 250 × 2 mm, particle diameter—4 μm, and pore diameter—90Å. The mobile phase was a gradient of water (solvent A) and acetonitrile (solvent B) with 0.01% (*v*/*v*) TFA. The gradient of solvent B had three stages: peptide separation (0 to 40%; 0.00–60.00 min); column washing (40–100%, 60.01–65.00 min; 100%, 65.01–70.00 min); column conditioning (100–0%, 70.00–71.00 min; 0%, 71.01–80.00 min). The sample concentration was 5 mg × mL^−1^ buffer solution (300 μL, pH = 6.6, with 0.1 M BIS-TRIS, 4.0 M urea) and 700 μL of buffer (pH = 2.2, with 6.0 M urea and TFA). The sample was centrifuged at 10,000 rpm for 10 min (Hermle Z 233, M-2, HERMLE LaborTechnik GmbH, Wehingen, Germany) [76]. The injection volume was 10 μL, the flow rate was 0.2 mL × min^−1^, and the column temperature was 30 °C. Chromatograms were acquired at the wavelength of 220 nm [77]. The collected data was analyzed using the Lab Solution (LC Solution) software provided by Shimadzu^®^. The RP-HPLC analyses were performed in duplicate.

### 3.8. Identification of ACE- and DPP-IV Inhibitors in Water-Soluble- Extracts of Different Gouda Cheese Variants

The identification of peptides was performed with the HPLC coupled with mass spectrometry (RP-HPLC-MS/MS) using a VARIAN^®^ 500-MS ion trap mass spectrometer (Agilent Technologies, Santa Clara, CA, USA) with an electrospray ion source and an HPLC assembly consisting of: two 212-LC pumps, a ProStar 410 autosampler, a Degassit degasser (MetaChem Technologies^®^, Torrance, CA, USA), and a nitrogen generator (Parker Domnick Hunter Scientific^®^, Gateshead, UK). The sample preparation and separation conditions, mobile phase, column type, and column parameters (Jupiter Proteo Phenomenex^®^ column, 250 × 2 mm, particle diameter—4 μm, pore diameter—90Å) were the same as for RP-HPLC-UV/Vis analysis but the separation gradient used was as follows: peptide separation (0 to 40%; 0.00–40.00 min); column washing (40–100%, 40.01–45.00 min, 100%, 45.01–50.00 min); column conditioning (100–0%, 50.01–51.00 min; 0%, 51.01–70.00 min). Data was collected in a 5–60 min time segment. The parameters of the mass spectrometer were as follows: needle and shield voltages: 5000 and 600 V, respectively; spraying and drying gas (nitrogen) pressure: 55 and 30 psi, respectively; drying gas temperature: 390 °C; flow rate of damping gas (helium): 0.8 mL × min^−1^; positive polarity with current ionization: 600 V, capillary voltage: 100 V; retardation factor loading: 100%; isolation window: 3.0 Da; excitation storage level: m/z = 100–2000 Da; frequency of data recording: 0.05–0.07 Hz; single scan averaged from 5 microscans; options such as: use of air segment, headspace pressure and alarm buzzer were included [44]. The chromatograms were analyzed using the MS WorkStation v. 6.9 software. All chromatograms were smoothed using the Savitzky and Golay [78] method. All analyses were performed in duplicate. Mass to charge ratios [m/z] of fragment ions were theoretically calculated using the Fragment Ion Calculator available at: http://db.systemsbiology.net:8080/proteomicsToolkit/FragIonServlet.html, accessed: 11 March 2020 [79]. The peptide sequence, in one-letter code, was pasted into the window called “Peptide” and the following software options were marked: “+1”, “+2”, and “+3” (function called “Charge state”) referring to mono-, double-, and triple-ionized ions, respectively, as well as “A, B, C, X, Y, Z” [52,80]. The submitted results included sequences of peptides to be potentially identified, their monoisotopic masses, and m/z of fragment ions. Identification was considered as successful if we found fragment ions providing complete sequence information, i.e., corresponding to the cleavage of all peptide bonds in a given compound.

### 3.9. Analysis and Visualization of Experimental Data

Heatmap was created to visualize the results concerning the presence of ACE- and DPP-IV- inhibiting peptides in the individual samples of Gouda cheese. The heatmap was created using the Heatmapper program [55] available at: http://www.heatmapper.ca/ (accessed: 10 July 2020) [56].

Finally, two parameters were calculated: the frequency of the released ACE or DPP-IV inhibitory fragments during cheese ripening (A_Eexp_.) and the relative frequency of the release of the fragments with the above-mentioned activities during cheese ripening (W_exp_.). They were described using the following equations, similar to those proposed previously by Minkiewicz et al [58]:

A_Eexp._ = d_exp._/N (3)

where:

- d_exp._—the number of peptides (ACE or DPP-IV inhibitors) from an individual protein, identified in cheese during its ripening;
- N—the number of amino acid residues in a protein (taken from the BIOPEP-UWM database).

W = A_Eexp._/A (4)

where:

A—the frequency of the occurrence of ACE or DPP-IV inhibitors in a protein sequence (taken from the BIOPEP-UWM database).

## 4. Conclusions

The protocol applied in our study led to confirm that all Gouda cheese variants exhibited ACE- and DPP-IV-inhibitory bioactivities. The ACE-inhibitory bioactivity was comparable for all cheese variants. More noticeable differences were observed when analyzing their DPP-IV-inhibiting effect. The water-soluble extracts derived from Gouda cheese with the increased content of β-casein (at both analyzed stages of ripening) had a better DPP-IV-inhibiting potency than the identical samples measured for their ACE inhibition. In turn, the heatmap showed the ACE inhibition to be the predominant activity of Gouda cheese. Such a phenomenon results from the sole presence, and not a quantity, of peptides in a sample as confirmed by the RP-HPLC-MS/MS. In turn, A_Eexp._ and W_exp._ values led to confirm that the samples derived from Gouda cheese after 60 days of ripening were the best sources of biopeptides, regardless of β-casein content. Our results might be the premise for further research involving in vivo studies on ACE-/DPP-IV inhibition by Gouda cheese with modified β-casein content.

## Figures and Tables

**Table 1:** Number of DPP-IV and ACE inhibitors potentially present in bovine casein sequences.

Casein/Gen. var.	F ^a^	Number of Peptides Present in Caseins														Total
		2- ^b^	3-	4-	5-	6-	7-	8-	9-	10-	11-	12-	13-	14-	19-	
α_S1_/A	ACE_i_^c^	72	6	4	2	5	1	2	1	-	-	-	-	-	-	93
	DPP-IV_i_^d^	113	1	-	-	-	-	-	-	-	-	-	-	-	-	114
α_S1_/B	ACE_i_	76	7	5	3	5	1	2	1	-	-	-	-	-	-	100
	DPP-IV_i_	121	1	-	-	-	-	-	-	-	-	-	-	-	-	122
α_S1_/C	ACE_i_	77	7	5	3	5	1	2	1	-	-	-	-	-	-	101
	DPP-IV_i_	120	1	-	-	-	-	-	-	-	-	-	-	-	-	121
α_S1_/D	ACE_i_	79	8	6	3	5	2	2	1	-	-	1	-	-	-	107
	DPP-IV_i_	131	1	-	-	-	-	-	-	-	-	-	-	-	-	132
α_S2_/A	ACE_i_	65	9	1	3	3	1	3	2	-	1	-	-	-	-	88
	DPP-IV_i_	151	1	1	-	1	-	-	-	-	-	-	-	-	-	154
β/A^1^	ACE_i_	66	14	5	4	6	7	5	5	4	1	1	1	1	-	120
	DPP-IV_i_	159	3	2	-	-	1	-	1	-	-	-	-	-	-	166
β/A^2^	ACE_i_	67	14	5	5	6	7	6	6	4	1	1	1	1	1	125
	DPP-IV_i_	160	3	2	-	-	2	-	1	-	-	-	-	-	-	168
β/A^3^	ACE_i_	66	14	5	4	6	7	5	5	4	1	1	1	1	-	120
	DPP-IV_i_	158	3	2	-	-	1	-	1	-	-	-	-	-	-	165
β/B	ACE_i_	69	14	6	6	7	8	5	6	3	1	1	1	1	1	129
	DPP-IV_i_	159	4	2	1	-	2	-	1	-	-	-	-	-	-	169
β/C	ACE_i_	67	14	5	4	6	7	5	5	4	1	1	1	1	-	121
	DPP-IV_i_	160	3	2	-	-	1	-	1	-	-	-	-	-	-	167
β/E	ACE_i_	68	14	5	5	6	7	6	6	4	1	1	1	1	1	126
	DPP-IV_i_	162	3	2	-	-	2	-	1	-	-	-	-	-	-	170
β/F	ACE_i_	65	13	5	4	6	7	5	5	4	1	1	1	1	-	118
	DPP-IV_i_	159	3	2	-	-	1	-	1	-	-	-	-	-	-	166
κ/A	ACE_i_	53	10	4	5	5	1	-	1	1	-	-	-	-	-	80
	DPP-IV_i_	142	2	1	2	-	-	-	-	-	-	-	-	-	-	147

^a^ biological function; ^b^ numbers from 2 to 19 refer to the number of residues in a peptide chain; ^c^ ACE_i_—ACE inhibitor; ^d^ DPP-IV_i_—dipeptidyl peptidase IV inhibitor.

**Table 2:** “Ranking” of casein sequences as the best potential sources of ACE- and DPP-IV inhibitors ^1^.

Casein/Genetic Variant	Total Number of ACE Inhibitors	Casein/Genetic Variant	Total Number of DPP-IV Inhibitors
β/B	129	β/E	170
β/E	126	β/B	169
β/A^2^	125	β/A^2^	168
β/C	121	β/C	167
β/A^1^	120	β/A^1^	166
β/A^3^	120	β/F	166
β/F	118	β/A^3^	165
α_S1_/D	107	α_S2_/A	154
α_S1_/C	101	κ/A	147
α_S1_/B	100	α_S1_/D	132
α_S1_/A	93	α_S1_/B	122
α_S2_/A	88	α_S1_/C	121
κ/A	80	α_S1_/A	114

^1^ based on the total number of peptidic ACE-/DPP-IV inhibitors found in casein sequences.

**Table 3:** The ratio of α_s_-casein (α_s_-CN) to β-casein (β-CN) and percentage of β-casein in all casein fractions of milks to produce different variants of Gouda cheese.

Milk Type	α_s_-CN/β-CN	β-CN (%)
MCN↓ ^1^	^a^ 2.58	^c^ 22.11
MCN-0	^b^ 1.52	^b^ 31.93
MCN↑	^c^ 1.28	^a^ 35.65

^1^ MCN↓, MCN-0, MCN↑ milk with reduced (↓), normative (0), and increased (↑) content of β-casein used to produce Gouda cheese; ^a,b,c^—means that share the same letter within the same column were not statistically significant (*p* ≥ 0.05).

**Table 4:** The composition of different variants of Gouda cheese (data concerns cheeses after 60 days of ripening).

Composition (% wt/wt)	ChCN↓ ^1^	Variant of Gouda Cheese	ChCN↑
		ChCN-0	
Moisture	40.19 ^b^	41.85 ^a^	39.88 ^b^
Dry matter	59.81 ^a^	58.15 ^b^	60.12 ^a^
Protein	27.28 ^a^	26.87 ^a^	26.51 ^a^
Fat	28.11 ^a^	27.18 ^a^	27.20 ^a^
Ash	3.78 ^a^	3.75 ^a^	4.06 ^a^

^1^ ChCN↓, ChCN-0-1, ChCN-0, ChCN↑—Gouda cheese with reduced (↓), normative (0), and increased (↑) content of β-casein, respectively; ^a,b^—means that share the same letter within the same row were not statistically significant (*p* ≥ 0.05).

**Table 5:** Peak area percentages calculated for water-soluble extracts of Gouda cheese with the modified β-casein content separated using RP-HPLC.

Time Segment (min)	Relative Peak Area (%) ^1^					
	ChCN↓-1 ^2^	ChCN↓-60	ChCN-0-1	ChCN-0-60	ChCN↑-1	ChCN↑-60
0.00–14.00	65.11	51.37	54.53	55.73	56.18	54.64
14.01–40.00	6.29	12.09	12.30	14.92	15.29	15.14
40.01–60.00	28.61	36.54	33.17	29.36	28.53	30. 22

^1^ Area of all peaks between 0.00 and 60.00 min is 100% (see Figure 1); ^2^ ChCN↓-1, ChCN↓-60, ChCN-0-1, ChCN-0-60, ChCN↑-1, ChCN↑-60, water-soluble extracts derived from Gouda cheese with: reduced (↓), normative (0), and increased (↑) content of β-casein after 1 (suffix 1) and 60 (suffix 60) days of ripening, respectively (see Methods).

**Table 6:** ACE- and DPP-IV-inhibiting (IC_50_) potential of the water-soluble extracts derived from Gouda with the modified content of β-casein.

IC_50_ (mg × mL^−1^)						
Sample/Activity	ChCN↓-1 ^1^	ChCN-0-1	ChCN↑-1	ChCN↓-60	ChCN-0-60	ChCN↑-60
ACE_i_^2^	14.840 ^a^	14.860 ^a^	14.740 ^a^	14.840 ^a^	14.590 ^a^	15.370 ^a^
DPP-IV_i_^3^	17.990 ^b^	18.760 ^b^	9.174 ^a^	18.300 ^b^	18.760 ^b^	9.171 ^a^

^1^ ChCN↓-1, ChCN↓-60, ChCN-0-1, ChCN-0-60, ChCN↑-1, ChCN↑-60—water-soluble extracts derived from Gouda cheese with: reduced (↓), normative (0), and increased (↑) content of β-casein after 1 (suffix 1) and 60 (suffix 60) days of ripening, respectively (see Methods); ^2^ ACE_i_—ACE-inhibitory activity; ^3^ DPP-IV_i_—dipeptidyl peptidase IV-inhibitory activity; ^a,b^—means that share the same letter within the same row were not statistically significant (*p* < 0.05).

**Table 7:** Values of A_Eexp._ and W_exp._ calculated for the ACE- and DPP-IV-inhibitory activity of peptides released from different Gouda cheese variants.

**ACE_i_^1^**														
**S ^2^**	**P ^3^**	**α_S1_^A 4^**	**α_S1_^B^**	**α_S1_^C^**	**α_S1_^D^**	**α_S2_^A^**	**β^A1^**	**β^A2^**	**β^A3^**	**β^B 8^**	**β^C^**	**β^E^**	**β^F^**	**κ^A^**
**ChCN↓-1**		I ^5^												
		7	9	9	9	6	10	10	10	**12**	10	10	10	6
	A_Eexp._^6^	0.038	0.045	0.045	0.042	0.027	0.048	0.048	0.048	**0.057**	0.048	0.048	0.048	0.032
	W_exp._^7^	0.075	0.090	0.089	0.084	0.068	0.083	0.080	0.083	**0.093**	0.083	0.079	0.085	0.075
**ChCN-0 -1**	P	I												
		7	9	9	9	7	10	10	10	**12**	10	10	10	7
	A_Eexp._	0.038	0.045	0.045	0.042	0.032	0.048	0.048	0.048	**0.057**	0.048	0.048	0.048	0.037
	W_exp._	0.075	0.090	0.089	0.084	0.080	0.083	0.080	0.083	**0.093**	0.083	0.079	0.085	0.088
**ChCN↑-1**	P	I												
		7	9	9	9	7	10	10	10	**12**	10	10	10	6
	A_Eexp._	0.038	0.045	0.045	0.042	0.032	0.048	0.048	0.048	**0.057**	0.048	0.048	0.048	0.032
	W_exp._	0.075	0.090	0.089	0.084	0.080	0.083	0.080	0.083	**0.093**	0.083	0.079	0.085	0.075
**ChCN↓-60**	P	I												
		11	12	12	**13**	5	12	12	12	**13**	12	12	12	6
	A_Eexp._	0.059	0.060	0.060	**0.061**	0.023	0.057	0.057	0.057	**0.062**	0.057	0.057	0.057	0.032
	W_exp_	0.118	0.120	0.119	**0.122**	0.057	0.100	0.096	0.100	0.101	0.099	0.095	0.102	0.075
**ChCN-0-60**	P	I												
		11	12	12	**13**	5	12	12	12	**13**	12	12	12	6
	A_Eexp._	0.059	0.060	0.060	**0.061**	0.023	0.057	0.057	0.057	**0.062**	0.057	0.057	0.057	0.032
	W_exp._	0.118	0.120	0.119	**0.122**	0.057	0.100	0.096	0.100	0.101	0.099	0.095	0.102	0.075
**ChCN↑-60**	P	I												
		11	12	12	13	5	13	13	13	**14**	13	13	13	7
	A_Eexp._	0.059	0.060	0.060	**0.061**	0.023	0.062	0.062	0.062	**0.067**	0.062	0.062	0.062	0.037
	W_exp._	0.118	0.120	0.119	**0.122**	0.057	0.108	0.104	0.108	0.109	0.107	0.103	0.110	0.088
**DPP-IV_i_^1^**														
**S**	P	α_S1_^A^	α_S1_^B^	α_S1_^C^	α_S1_^D^	α_S2_^A^	β^A1^	β^A2^	β^A3^	**β^B^**	β^C^	β^E^	β^F^	κ^A^
**ChCN↓-1**		I												
		2	2	2	2	**4**	3	3	3	**4**	3	3	3	3
	A_Eexp_	0.011	0.010	0.010	0.009	0.018	0.014	0.014	0.014	**0.019**	0.014	0.014	0.014	0.016
	W_exp_	0.018	0.016	0.017	0.015	**0.026**	0.018	0.018	0.018	0.024	0.018	0.018	0.018	0.020
**ChCN-0 -1**	P	I												
		2	2	2	2	**4**	3	3	3	**4**	3	3	3	3
	A_Eexp._	0.011	0.010	0.010	0.009	0.018	0.014	0.014	0.014	**0.019**	0.014	0.014	0.014	0.016
	W_exp_	0.018	0.016	0.017	0.015	**0.026**	0.018	0.018	0.018	0.024	0.018	0.018	0.018	0.020
**ChCN↑-1**	P	I												
		2	2	2	2	**4**	3	3	3	**4**	3	3	3	3
	A_Eexp._	0.011	0.010	0.010	0.009	0.018	0.014	0.014	0.014	**0.019**	0.014	0.014	0.014	0.016
	W_exp._	0.018	0.016	0.017	0.015	**0.026**	0.018	0.018	0.018	0.024	0.018	0.018	0.018	0.020
**ChCN↓-60**	P	I												
		2	2	2	2	**7**	4	4	4	4	4	4	4	4
	A_Eexp._	0.011	0.010	0.010	0.009	**0.032**	0.019	0.019	0.019	0.019	0.019	0.019	0.019	0.021
	W_exp._	0.018	0.016	0.017	0.015	**0.046**	0.024	0.024	0.024	0.024	0.024	0.024	0.024	0.027
**ChCN-0-60**	P	I												
		2	2	2	2	**7**	4	4	4	4	4	4	4	4
	A_Eexp._	0.011	0.010	0.010	0.009	**0.032**	0.019	0.019	0.019	0.019	0.019	0.019	0.019	0.021
	W_exp._	0.018	0.016	0.017	0.015	**0.046**	0.024	0.024	0.024	0.024	0.024	0.024	0.024	0.027
**ChCN↑-60**	P	I												
		2	2	2	2	**7**	4	4	4	4	4	4	4	4
	A_Eexp._	0.011	0.010	0.010	0.009	**0.032**	0.019	0.019	0.019	0.019	0.019	0.019	0.019	0.021
	W_exp._	0.018	0.016	0.017	0.015	**0.046**	0.024	0.024	0.024	0.024	0.024	0.024	0.024	0.027

^1^ ACE_i_ and DPP-IV_i_—ACE-and DPP-IV-inhibitory activity, respectively; ^2^ S—cheese sample: ChCN↓-1, ChCN↓-60, ChCN-0-1, ChCN-0-60, ChCN↑-1, ChCN↑-60—water-soluble extracts derived from Gouda cheese with: reduced (↓), normative (0), and increased (↑) content of β-casein after 1 (suffix 1) and 60 (suffix 60) days of ripening, respectively; ^3^ P—parameter; ^4^ casein source including genetic variant provided in a superscript; ^5^ I—number of experimentally identified peptides; ^6^ A_Eexp._—the frequency of the released ACE or DPP-IV inhibitory fragments during cheese ripening; ^7^ W_exp._—the relative frequency of the release of the fragments with ACE- or DPP-IV-inhibitory activity during cheese ripening; ^8^ bold—the highest values of individual parameters.

## Data Availability

The data presented in this study are available in present article as well as Supplementary Materials (Tables S1 and S2, Figures S1–S64).

## Supplementary Materials

The following are available online at https://www.mdpi.com/1422-0067/22/6/2949/s1, Table S1: Peptides with ACE- and DPP-IV-inhibitory activity encrypted in casein sequences. Data obtained using the BIOPEP-UWM database [13,61] (accessed January–March 2020), Table S2: RP-HPLC-MS/MS identification of peptides with ACE and/or DPP-IV-inhibitory activity in different Gouda cheese variants, Figure S1: RP-HPLC chromatograms of water-soluble extracts derived from Gouda cheese with modified β-casein content before and after ripening (1st and 60th day, respectively), within the time interval 1–10 min, Figure S2: RP-HPLC chromatograms of water-soluble extracts derived from Gouda cheese with modified β-casein content before and after ripening (1st and 60th day, respectively), within the time interval 10–60 min, Figures S3–S64: MS/MS spectra of peptides.

## Abbreviations

ACE	angiotensin-converting enzyme (EC 3.4.15.1)
ACE_i_	ACE inhibitor
A_Eexp._	the frequency of the released ACE or DPP-IV inhibitory fragments during cheese ripening described by the following equation: A_Eexp._ = d_exp._/N, where: d_exp._—the number of peptides (ACE or DPP-IV inhibitors) identified in cheese during its ripening; N—the number of amino acid residues from an individual protein (taken from the BIOPEP-UWM database)
ACN	acetonitrile
Ala	alanine
Arg	arginine
BIOPEP-UWM	database of bioactive peptide sequences [13,61]
Bis-TRIS	2,2-bis(hydroxymethyl)-2,2′,2″-nitrilotriethanol
ChCN-0	Gouda cheese with the normative content of β-casein
ChCN-0-1	water-soluble extracts derived from Gouda cheese with the normative content of β-casein after 1 day of ripening
ChCN-0-60	water-soluble extracts derived from Gouda cheese with the normative content of β-casein after 60 days of ripening
ChCN↑	Gouda cheese with the increased content of β-casein
ChCN↑-1	water-soluble extracts derived from Gouda cheese with the increased content of β-casein after 1 day of ripening
ChCN↑-60	water-soluble extracts derived from Gouda cheese with the increased content of β-casein after 60 days of ripening
ChCN↓	Gouda cheese with the reduced content of β-casein
ChCN↓-1	water-soluble extracts derived from Gouda cheese with the reduced content of β-casein after 1 day of ripening
ChCN↓-60	water-soluble extracts derived from Gouda cheese with the reduced content of β-casein after 60 days of ripening
CF	Concentration Factor
CN	casein
DF	diafiltrate
DPP IV	dipeptidyl peptidase IV (EC 3.4.14.5)
DPP-IV_i_	dipeptidyl peptidase IV inhibitor
GIP	glucose-dependent insulinotropic polypeptide
Gly-Pro-*p*-NA	glycyl-prolyl-*p*-nitroanilide
Gln	glutamine
Glu	glutamic acid
HA	hippuric acid
HHL	hippuryl-histidyl-leucine
HPLC	High Performance Liquid Chromatography
IC_50_	concentration of a sample corresponding to is half inhibitory activity (mg x mL^−1^)
Ile	isoleucine
LAB	lactic acid bacteria
Leu	leucine
Lys	lysine
MCN↓	milk used to produce Gouda cheese with the reduced content of β-CN
MCN-0	milk used to produce Gouda cheese with the normative content of β-CN
MCN↑	milk used to produce Gouda cheese with the increased content of β-CN
MF	microfiltrate
m/z	mass-to-charge ratio
PDF7	diafiltration permeate produced at 7 °C
PMF50	microfiltration permeate produced at 50 °C
PMF7	microfiltration permeate produced at 7 °C
*p*-NA	p-nitroaniline
Pro	proline
PRUF	ultrafiltration retentate from MF/DF permeates
PUF50	ultrafiltration permeate produced at 50 °C
QSAR	Quantitative Structure-Activity Relationship
RDF7	diafiltration retentate produced at 7 °C
RMF50	microfiltration retentate produced at 50 °C
RMF7	microfiltration retentate produced at 7 °C
RP-HPLC	Reversed-Phase High Performance Liquid Chromatography
RP-HPLC-MS/MS	Reversed-Phase High Performance Liquid Chromatography on-line with tandem Mass Spectrometry
RP-HPLC-UV/Vis	Reversed-Phase High Performance Liquid Chromatography with UV/Vis detector
RUF50	ultrafiltration retentate produced at 50 °C
SDS-PAGE	Sodium Dodecyl Sulfate Polyacrylamide Gel Electrophoresis
Ser	serine
SW	polyethersulfone spiral-wound membrane
TFA	trifluoroacetic acid
Tre	threonine
TRIS-HCl	tris(hydroxymethyl)aminomethane hydrochloride
t_R_	retention time (min)
TN	total nitrogen content
Tyr	tyrosine
UF	ultrafiltrate
UWM	University of Warmia and Mazury in Olsztyn, Poland
W_exp._	the relative frequency of the release of the fragments with above-mentioned activities during cheese ripening described by the following equation: W = A_Eexp._/A where: A—the frequency of the occurrence of ACE or DPP-IV inhibitors in a protein sequence (taken from the BIOPEP-UWM database); A_Eexp._—see above.
Val	valine
