# Supplementary material for: Gouda Cheese with Modified Content of β-Casein as a Source of Peptides with ACE- and DPP-IV-Inhibiting Bioactivity: A Study Based on In Silico and In Vitro Protocol
Source: Int J Mol Sci. 2021 Mar 14;22(6):2949. doi: 10.3390/ijms22062949 (PMC8001443; doi:10.3390/ijms22062949)
Supplement: Supplementary file 1 [file ijms-22-02949-s001.pdf]

Supplement

Gouda cheese with modified content of β-casein as a source of peptides with ACE- and DPP-IV-inhibiting bioactivity: a study based on *in silico* and *in vitro* protocol

Anna Iwaniak, Damir Mogut, Piotr Minkiewicz, Justyna Żulewska and Małgorzata Darewicz

Table S1. Peptides with ACE- and DPP-IV-inhibitory activity encrypted in casein sequences. Data obtained using the BIOPEP-UWM database [13,61] (accessed January-March 2020).

Sequence	Number of ACE- and DPP-IV inhibitors encrypted in a specific casein sequence/variant																									
	α_{s1}/A		α_{s1}/B		α_{s1}/C		α_{s1}/D		α_{s2}/A		β/A1		β/A2		β/A3		β/B		β/C		β/E		β/F		κ/A	
	Activity																									
	A[1]	D[2]	A	D	A	D	A	D	A	D	A	D	A	D	A	D	A	D	A	D	A	D	A	D	A	D
**AA**[3]	-	-	-	-	-	-	-	-	-	-	-	-	-	-	-	-	-	-	-	-	-	-	-	-	1	1
AE	-	2	-	2	-	2	-	2	-	1	-	-	-	-	-	-	-	-	-	-	-	-	-	-	-	-
**AF**	-	-	-	-	-	-	-	-	-	-	1	1	1	1	1	1	1	1	1	1	1	1	1	1	-	-
AI	-	-	-	-	-	-	-	-	1	-	-	-	-	-	-	-	-	-	-	-	-	-	-	-	1	-
AL	-	-	-	-	-	-	-	1	-	3	-	-	-	-	-	-	-	-	-	-	-	-	-	-	-	2
**AP**	1	1	2	2	2	2	2	2	-	-	1	1	1	1	1	1	1	1	1	1	1	1	1	1	-	-
AR	-	-	-	-	-	-	1	-	-	-	-	-	-	-	-	-	-	-	-	-	-	-	-	-	1	-
AS	-	-	-	-	-	-	-	-	-	-	-	-	-	-	-	-	-	-	-	-	-	-	-	-	-	1
AT	-	-	-	-	-	-	-	-	-	1	-	-	-	-	-	-	-	-	-	-	-	-	-	-	-	1
AV	-	-	-	-	-	-	-	1	-	2	-	1	-	1	-	1	-	1	-	1	-	1	-	1	-	3
**AW**	1	1	1	1	1	1	1	1	-	-	-	-	-	-	-	-	-	-	-	-	-	-	-	-	-	-
**AY**	2	2	2	2	2	2	2	2	-	-	-	-	-	-	-	-	-	-	-	-	-	-	-	-	-	-
DA	2	-	2	-	2	-	2	-	-	-	-	-	-	-	-	-	-	-	-	-	-	-	-	-	-	-
DQ	-	1	-	1	-	1	-	1	-	1	-	-	-	-	-	-	-	-	-	-	-	-	-	-	-	-
EA	1	-	1	-	1	-	1	-	-	-	1	-	1	-	1	-	1	-	1	-	1	-	1	-	1	-

**EG**	-	-	-	-	-	-	1	1	-	-	-	-	-	-	-	-	-	-	-	-	-	-	-	-	-	-
EH	-	-	-	-	-	-	-	-	-	1	-	-	-	-	-	-	-	-	-	-	-	-	-	-	-	-
**EI**	2	2	2	2	2	2	2	2	1	1	1	1	1	1	1	1	1	1	1	1	1	1	1	1	2	2
**EK**	2	2	2	2	1	1	2	2	2	2	2	2	2	2	2	2	1	1	3	3	2	2	2	2	1	1
EP	-	1	-	1	-	1	-	1	-	-	-	-	-	-	-	-	-	2	-	-	-	-	-	-	-	1
ES	-	2	-	2	-	2	-	2	-	3	-	3	-	3	-	3	-	2	-	3	-	3	-	3	-	2
ET	-	-	-	-	-	-	-	-	-	1	-	-	-	-	-	-	-	-	-	-	-	-	-	-	-	-
**EV**	-	-	1	1	1	1	2	2	4	4	1	1	1	1	1	1	1	1	1	1	1	1	1	1	1	1
**EY**	-	-	-	-	-	-	-	-	1	1	-	-	-	-	-	-	-	-	-	-	-	-	-	-	-	-
FA	-	-	-	-	-	-	-	-	-	1	-	1	-	1	-	1	-	1	-	1	-	1	-	1	-	-
FG	1	-	1	-	1	-	1	-	-	-	-	-	-	-	-	-	-	-	-	-	-	-	-	-	-	-
FL	-	-	-	-	-	-	-	-	-	1	-	2	-	2	-	2	-	2	-	2	-	2	-	2	-	3
**FP**	1	1	1	1	1	1	1	1	1	1	4	4	4	4	4	4	4	4	4	4	4	4	4	4	-	-
FQ	-	-	-	-	-	-	-	-	-	-	-	1	-	1	-	1	-	1	-	1	-	1	-	1	-	-
**FR**	1	1	1	1	1	1	1	1	-	-	-	-	-	-	-	-	-	-	-	-	-	-	-	-	-	-
FY	2	-	2	-	2	-	2	-	1	-	-	-	-	-	-	-	-	-	-	-	-	-	-	-	-	-
**GA**	1	1	1	1	1	1	1	1	-	-	-	-	-	-	-	-	-	-	-	-	-	-	-	-	1	1
**GE**	-	-	-	-	-	-	-	-	-	-	1	1	1	1	1	1	1	1	1	1	1	1	1	1	1	1
**GI**	1	1	1	1	1	1	1	1	-	-	-	-	-	-	-	-	-	-	-	-	-	-	-	-	-	-
GK	1	-	1	-	2	-	1	-	-	-	-	-	-	-	-	-	-	-	-	-	-	-	-	-	-	-
**GL**	1	1	1	1	1	1	1	1	-	-	-	-	-	-	-	-	-	-	-	-	-	-	-	-	1	1
**GP**	-	-	-	-	-	-	-	-	1	1	3	3	3	3	3	3	3	3	3	3	3	3	3	3	-	-
GS	2	-	2	-	2	-	2	-	1	-	-	-	-	-	-	-	-	-	-	-	-	-	-	-	-	-
GT	1	-	1	-	1	-	1	-	-	-	-	-	-	-	-	-	-	-	-	-	-	-	-	-	-	-
**GV**	1	1	1	1	1	1	1	1	-	-	1	1	1	1	1	1	1	1	1	1	1	1	1	1	-	-
**GY**	1	1	1	1	1	1	1	1	-	-	-	-	-	-	-	-	-	-	-	-	-	-	-	-	-	-
HA	-	1	-	1	-	1	-	1	-	-	-	-	-	-	-	-	-	-	-	-	-	-	-	-	-	-
HI	-	1	-	1	-	1	-	1	-	-	-	-	-	-	-	-	-	-	-	-	-	-	-	-	-	-
HK	-	-	-	-	-	-	-	-	-	-	1	-	1	-	-	-	1	-	1	-	1	-	1	-	-	-
**HL**	-	-	-	-	-	-	-	-	-	-	1	1	1	1	1	1	1	1	1	1	1	1	1	1	1	1
**HP**	1	1	1	1	1	1	1	1	-	-	1	1	1	1	1	1	1	1	1	1	1	1	1	1	2	2
HS	-	1	-	1	-	1	-	1	-	-	-	-	-	-	-	-	-	-	-	-	-	-	-	-	-	-
HV	-	-	-	-	-	-	-	-	-	1	-	-	-	-	-	-	-	-	-	-	-	-	-	-	-	-

**HY**	-	-	-	-	-	-	-	-	1	1	-	-	-	-	-	-	-	-	-	-	-	-	-	-	-	-
**IA**	-	-	-	-	-	-	-	-	-	-	-	-	-	-	-	-	-	-	-	-	-	-	-	-	2	2
IE	-	-	-	-	-	-	-	-	-	-	1	-	1	-	1	-	1	-	1	-	1	-	1	-	1	-
IF	-	-	-	-	-	-	-	-	1	-	-	-	-	-	-	-	-	-	-	-	-	-	-	-	-	-
IG	3	-	3	-	3	-	3	-	1	-	-	-	-	-	-	-	-	-	-	-	-	-	-	-	-	-
IH	-	1	-	1	-	1	-	1	-	-	-	2	-	1	-	2	-	1	-	2	-	1	-	2	-	-
II	-	-	-	-	-	-	-	-	-	1	-	1	-	1	-	1	-	1	-	1	-	1	-	1	-	-
IL	-	-	-	-	-	-	-	1	-	-	-	-	-	-	-	-	-	-	-	-	-	-	-	-	-	2
IN	-	-	-	-	-	-	-	-	-	2	-	1	-	1	-	1	-	1	-	1	-	1	-	1	-	3
**IP**	1	1	1	1	1	1	1	1	1	1	1	1	2	2	1	1	2	2	1	1	2	2	1	1	3	3
IQ	-	1	-	1	-	1	-	1	-	1	-	1	-	1	-	1	-	1	-	1	-	1	-	1	-	1
**IR**	-	-	-	-	-	-	-	-	-	-	-	-	-	-	-	-	-	-	-	-	-	-	-	-	1	1
**KA**	-	-	-	-	-	-	-	-	2	2	1	1	1	1	1	1	1	1	1	1	1	1	1	1	-	-
**KE**	4	4	4	4	4	4	4	4	2	2	2	2	2	2	2	2	2	2	2	2	3	3	2	2	-	-
**KF**	-	-	-	-	-	-	-	-	3	3	1	1	1	1	1	1	1	1	1	1	1	1	1	1	-	-
KH	-	3	-	3	-	3	-	3	-	1	-	1	-	1	-	-	-	1	-	1	-	1	-	1	-	-
KI	-	-	-	-	-	-	-	-	-	2	-	2	-	2	-	2	-	2	-	2	-	2	-	2	-	1
KK	-	1	-	1	-	1	-	1	-	3	-	1	-	1	-	1	-	1	-	1	-	1	-	1	-	1
KL	-	-	-	-	-	-	1	-	1	-	-	-	-	-	-	-	-	-	-	-	-	-	-	-	-	-
**KP**	-	-	-	-	-	-	-	-	1	1	-	-	-	-	-	-	-	-	-	-	-	-	-	-	2	2
**KR**	-	-	-	-	-	-	-	-	1	1	-	-	-	-	-	-	-	-	-	-	-	-	-	-	-	-
KS	-	-	-	-	-	-	-	-	-	-	-	-	-	-	-	-	-	-	-	-	-	-	-	-	-	2
KT	-	1	-	1	-	1	-	1	-	4	-	-	-	-	-	-	-	-	-	-	-	-	-	-	-	1
KV	-	2	-	2	-	2	-	2	-	1	-	2	-	2	-	2	-	2	-	2	-	2	-	2	-	-
**KY**	1	1	1	1	1	1	1	1	-	-	1	1	1	1	1	1	1	1	1	1	1	1	1	1	1	1
**LA**	1	1	1	1	1	1	2	2	2	2	-	-	-	-	-	-	-	-	-	-	-	-	-	-	1	1
LF	1	-	1	-	1	-	1	-	-	-	-	-	-	-	-	-	-	-	-	-	-	-	-	-	-	-
LG	2	-	2	-	2	-	2	-	-	-	1	-	1	-	1	-	1	-	1	-	1	-	1	-	1	-
LH	-	1	-	1	-	1	-	1	-	-	-	1	-	1	-	1	-	1	-	1	-	1	-	1	-	-
LI	-	-	-	-	-	-	-	1	-	-	-	-	-	-	-	-	-	-	-	-	-	-	-	-	-	1
LL	-	1	-	2	-	2	-	3	-	1	-	3	-	3	-	3	-	2	-	3	-	3	-	4	-	-
**LN**	-	-	1	1	1	1	1	1	4	4	1	1	1	1	1	1	1	1	1	1	1	1	1	1	1	1
LP	-	1	-	1	-	1	-	1	-	1	-	4	-	4	-	4	-	5	-	4	-	4	-	4	-	2

LQ	-	-	-	-	-	-	-	-	1	-	3	-	3	-	3	-	3	-	3	-	3	-	3	-	1	-
LT	-	-	-	-	-	-	-	1	-	1	-	3	-	3	-	3	-	3	-	3	-	3	-	3	-	1
LV	-	-	-	-	-	-	-	1	-	-	-	1	-	1	-	1	-	1	-	1	-	1	-	1	-	1
**LW**	1	1	1	1	1	1	1	1	-	-	-	-	-	-	-	-	-	-	-	-	-	-	-	-	-	-
LY	-	-	-	-	-	-	-	-	1	-	1	-	1	-	1	-	1	-	1	-	1	-	1	-	-	-
MA	-	-	-	-	-	-	-	-	-	1	-	1	-	1	-	1	-	1	-	1	-	1	-	1	-	2
**ME**	2	2	2	2	2	2	2	2	2	2	-	-	-	-	-	-	-	-	-	-	-	-	-	-	-	-
**MF**	-	-	-	-	-	-	-	-	-	-	1	1	1	1	1	1	1	1	1	1	1	1	1	1	-	-
**MG**	-	-	-	-	-	-	-	-	-	-	1	1	1	1	1	1	1	1	1	1	1	1	1	1	-	-
MH	-	-	-	-	-	-	-	-	-	-	-	1	-	1	-	1	-	1	-	1	-	1	-	1	-	-
MI	-	1	-	1	-	1	-	1	-	-	-	-	-	-	-	-	-	-	-	-	-	-	-	-	-	-
MK	-	1	-	1	-	1	-	2	-	2	-	-	-	-	-	-	-	-	-	-	-	-	-	-	-	1
MM	-	-	-	-	-	-	-	-	-	-	-	-	-	-	-	-	-	-	-	-	-	-	-	-	-	1
MP	-	1	-	1	-	1	-	1	-	-	-	2	-	2	-	2	-	2	-	2	-	2	-	2	-	-
NA	-	-	-	-	-	-	-	-	-	2	-	-	-	-	-	-	-	-	-	-	-	-	-	-	-	-
NE	-	1	-	2	-	2	-	2	-	2	-	-	-	-	-	-	-	-	-	-	-	-	-	-	-	-
**NF**	-	-	-	-	-	-	-	-	1	1	-	-	-	-	-	-	-	-	-	-	-	-	-	-	-	-
NK	-	-	-	-	-	-	-	-	-	-	1	-	1	-	1	-	1	-	1	-	1	-	1	-	-	-
NL	-	-	-	1	-	1	-	1	-	1	-	1	-	1	-	1	-	1	-	1	-	1	-	1	-	-
NM	-	-	-	-	-	-	-	-	-	1	-	-	-	-	-	-	-	-	-	-	-	-	-	-	-	-
NN	-	-	-	-	-	-	-	-	-	-	-	-	-	-	-	-	-	-	-	-	-	-	-	-	-	1
NP	-	1	-	1	-	1	-	1	-	2	-	-	-	-	-	-	-	-	-	-	-	-	-	-	-	-
NQ	-	1	-	1	-	1	-	1	-	1	-	-	-	-	-	-	-	-	-	-	-	-	-	-	-	3
NR	-	-	-	-	-	-	-	-	-	2	-	-	-	-	-	-	-	-	-	-	-	-	-	-	-	-
NT	-	-	-	-	-	-	-	-	-	1	-	-	-	-	-	-	-	-	-	-	-	-	-	-	-	3
NV	-	-	-	-	-	-	-	-	-	-	-	1	-	1	-	1	-	1	-	1	-	1	-	1	-	-
**NY**	-	-	-	-	-	-	-	-	-	-	-	-	-	-	-	-	-	-	-	-	-	-	-	-	1	1
PA	-	-	-	-	-	-	-	-	-	-	-	-	-	-	-	-	-	-	-	-	-	-	-	-	-	3
PF	-	1	-	1	-	1	-	1	-	-	-	6	-	6	-	6	-	6	-	6	-	6	-	6	-	1
**PG**	-	-	-	-	-	-	-	-	-	-	2	2	2	2	2	2	2	2	2	2	2	2	2	2	-	-
**PH**	-	-	-	-	-	-	-	-	-	-	1	1	1	1	1	1	1	1	1	1	1	1	1	1	2	2
PI	-	2	-	2	-	2	-	2	-	2	-	3	-	3	-	3	-	3	-	3	-	3	-	3	-	2
PK	-	1	-	1	-	1	-	1	-	1	-	2	-	2	-	2	-	2	-	2	-	2	-	2	-	1

**PL**	2	2	2	2	2	2	2	2	-	-	3	3	3	3	3	3	4	4	3	3	3	3	3	3	-	-
PM	-	1	-	1	-	1	-	1	-	-	-	-	-	-	-	-	-	-	-	-	-	-	-	-	-	-
PN	-	3	-	3	-	3	-	3	-	-	-	-	-	1	-	-	-	1	-	-	-	1	-	-	-	-
**PP**	-	-	-	-	-	-	-	-	-	-	5	5	5	5	5	5	4	4	5	5	5	5	4	4	2	2
**PQ**	3	3	3	3	3	3	2	2	2	2	3	3	3	3	3	3	4	4	3	3	3	3	3	3	-	-
PS	-	3	-	3	-	3	-	3	-	1	-	-	-	-	-	-	-	-	-	-	-	-	-	-	-	1
**PT**	-	-	-	-	-	-	-	-	1	1	1	1	1	1	1	1	1	1	1	1	1	1	1	1	4	4
PV	-	-	-	-	-	-	-	-	-	-	-	5	-	5	-	5	-	5	-	5	-	5	-	5	-	1
PW	-	-	-	-	-	-	-	-	-	2	-	-	-	-	-	-	-	-	-	-	-	-	-	-	-	-
PY	-	-	-	-	-	-	-	-	-	1	-	1	-	1	-	1	-	1	-	1	-	1	-	1	-	2
QA	-	1	-	1	-	1	-	1	-	-	-	1	-	1	-	1	-	1	-	1	-	1	-	1	-	1
QD	-	-	-	-	-	-	-	-	-	-	-	1	-	1	-	1	-	1	-	1	-	1	-	1	-	1
QE	-	1	-	2	-	2	-	2	-	2	-	-	-	-	-	-	-	1	-	-	-	-	-	-	-	2
QF	-	1	-	1	-	1	-	1	-	1	-	-	-	-	-	-	-	-	-	-	-	-	-	-	-	1
**QG**	2	2	2	2	2	2	1	1	1	1	-	-	-	-	-	-	-	-	-	-	-	-	-	-	-	-
QH	-	-	-	-	-	-	-	-	-	1	-	-	-	-	-	-	-	-	-	-	-	-	-	-	-	-
QI	-	-	-	-	-	-	-	-	-	-	-	-	-	-	-	-	-	-	-	-	-	-	-	-	-	1
QK	3	-	3	-	3	-	3	-	4	-	-	-	-	-	1	-	1	-	-	-	-	-	-	-	1	-
QL	-	3	-	3	-	3	-	3	-	1	-	-	-	-	-	-	-	-	-	-	-	-	-	-	-	-
QN	-	-	-	-	-	-	-	-	-	-	-	1	-	1	-	1	-	1	-	1	-	1	-	1	-	1
QP	-	1	-	-	-	-	-	-	-	1	-	5	-	5	-	5	-	3	-	5	-	5	-	5	-	2
QQ	-	1	-	1	-	1	-	1	-	-	-	3	-	3	-	3	-	2	-	3	-	3	-	3	-	1
QS	-	-	-	-	-	-	-	-	-	-	-	6	-	6	-	6	-	6	-	6	-	6	-	6	-	-
QT	-	-	-	-	-	-	-	-	-	-	-	3	-	3	-	3	-	3	-	3	-	3	-	3	-	-
QV	-	1	-	1	-	1	-	-	-	1	-	-	-	-	-	-	-	-	-	-	-	-	-	-	-	2
QW	-	-	-	-	-	-	-	-	-	-	-	-	-	-	-	-	-	-	-	-	-	-	-	-	-	1
QY	-	1	-	1	-	1	-	1	-	3	-	-	-	-	-	-	-	-	-	-	-	-	-	-	-	1
RF	-	-	1	-	1	-	1	-	-	-	-	-	-	-	-	-	-	-	-	-	-	-	-	-	1	-
RG	-	-	-	-	-	-	-	-	-	-	-	1	-	1	-	1	-	1	-	1	-	1	-	1	-	-
RH	-	-	-	-	-	-	-	-	-	-	-	-	-	-	-	-	-	-	-	-	-	-	-	-	-	1
RI	-	-	-	-	-	-	-	-	-	-	-	1	-	1	-	1	-	1	-	1	-	1	-	1	-	-
**RL**	2	2	2	2	2	2	2	2	1	1	-	-	-	-	-	-	-	-	-	-	-	-	-	-	-	-
RN	-	-	-	-	-	-	-	-	-	2	-	-	-	-	-	-	-	-	-	-	-	-	-	-	-	-

**RP**	1	1	1	1	1	1	1	1	-	-	-	-	-	-	-	-	-	-	-	-	-	-	-	-	-	-
RY	1	-	1	-	1	-	1	-	2	-	-	-	-	-	-	-	-	-	-	-	-	-	-	-	1	-
**SF**	1	1	1	1	1	1	1	1	-	-	-	-	-	-	-	-	-	-	-	-	-	-	-	-	2	2
SG	1	-	1	-	2	-	1	-	-	-	-	-	-	-	-	-	-	-	-	-	-	-	-	-	1	-
SI	-	1	-	1	-	1	-	1	-	2	-	1	-	1	-	1	-	1	-	1	-	1	-	1	-	-
SK	-	1	-	1	-	1	-	1	-	2	-	2	-	2	-	2	-	2	-	2	-	3	-	2	-	-
SL	-	-	-	-	-	-	-	-	-	-	-	5	-	5	-	5	-	5	-	5	-	5	-	5	-	-
SP	-	-	-	-	-	-	-	-	-	-	-	-	-	-	-	-	-	-	-	-	-	-	-	-	-	3
SV	-	1	-	1	-	1	-	1	-	-	-	1	-	1	-	1	-	1	-	1	-	1	-	1	-	-
SW	-	-	-	-	-	-	-	-	-	-	-	1	-	1	-	1	-	1	-	1	-	1	-	1	-	-
**SY**	-	-	-	-	-	-	-	-	-	-	-	-	-	-	-	-	-	-	-	-	-	-	-	-	1	1
TA	-	-	-	-	-	-	-	-	-	-	-	-	-	-	-	-	-	-	-	-	-	-	-	-	-	1
TD	-	1	-	1	-	1	-	1	-	-	-	1	-	1	-	1	-	1	-	1	-	1	-	1	-	-
**TE**	1	1	1	1	1	1	1	1	3	3	2	2	2	2	2	2	2	2	2	2	2	2	2	2	2	2
**TF**	-	-	-	-	-	-	-	-	1	1	-	-	-	-	-	-	-	-	-	-	-	-	-	-	-	-
TI	-	-	-	-	-	-	-	-	-	-	-	-	-	-	-	-	-	-	-	-	-	-	-	-	-	3
TK	-	-	-	-	-	-	-	-	-	3	-	-	-	-	-	-	-	-	-	-	-	-	-	-	-	-
TL	-	-	-	-	-	-	-	-	-	1	-	1	-	1	-	1	-	1	-	1	-	1	-	1	-	2
TM	-	1	-	1	-	1	-	1	-	1	-	-	-	-	-	-	-	-	-	-	-	-	-	-	-	1
TP	-	-	-	-	-	-	-	-	-	1	-	1	-	1	-	1	-	1	-	1	-	1	-	1	-	1
**TQ**	1	1	1	1	1	1	1	1	-	-	2	2	2	2	2	2	2	2	2	2	2	2	2	2	-	-
TR	-	-	-	-	-	-	-	-	-	-	-	1	-	1	-	1	-	1	-	1	-	1	-	1	-	-
TS	-	-	-	-	-	-	-	-	-	1	-	-	-	-	-	-	-	-	-	-	-	-	-	-	-	2
TT	-	1	-	1	-	1	-	1	-	-	-	-	-	-	-	-	-	-	-	-	-	-	-	-	-	2
TV	-	-	-	-	-	-	-	-	-	3	-	1	-	1	-	1	-	1	-	1	-	1	-	1	-	3
TY	-	-	-	-	-	-	-	-	-	1	-	-	-	-	-	-	-	-	-	-	-	-	-	-	-	-
VA	-	-	-	1	-	1	-	3	-	2	-	-	-	-	-	-	-	-	-	-	-	-	-	-	-	2
VD	-	-	-	-	-	-	-	-	-	2	-	-	-	-	-	-	-	-	-	-	-	-	-	-	-	-
**VE**	1	1	1	1	1	1	1	1	-	-	2	2	2	2	2	2	3	3	2	2	2	2	2	2	1	1
**VF**	1	1	1	1	1	1	1	1	1	1	-	-	-	-	-	-	-	-	-	-	-	-	-	-	-	-
VI	-	-	-	-	-	-	-	-	-	1	-	-	-	-	-	-	-	-	-	-	-	-	-	-	-	1
**VK**	-	-	-	-	-	-	-	-	2	2	1	1	1	1	1	1	1	1	1	1	1	1	1	1	-	-
VL	-	-	-	1	-	1	-	1	-	1	-	3	-	3	-	3	-	3	-	3	-	3	-	3	-	2

VM	-	-	-	-	-	-	-	-	-	-	-	2	-	2	-	2	-	2	-	2	-	2	-	2	-	-
VN	-	2	-	2	-	2	-	2	-	-	-	-	-	-	-	-	-	-	-	-	-	-	-	-	-	-
**VP**	5	5	5	5	5	5	5	5	1	1	4	4	4	4	4	4	4	4	4	4	4	4	4	4	1	1
VQ	-	-	-	-	-	-	-	-	-	-	-	1	-	1	-	1	-	-	-	1	-	1	-	1	-	1
**VR**	-	-	-	-	-	-	-	-	2	2	1	1	1	1	1	1	1	1	1	1	1	1	1	1	1	1
VS	-	-	-	-	-	-	-	-	-	1	-	1	-	1	-	1	-	1	-	1	-	1	-	1	-	-
VT	-	-	-	-	-	-	-	-	-	-	-	-	-	-	-	-	-	-	-	-	-	-	-	-	-	2
VV	-	-	-	-	-	-	-	-	-	1	-	2	-	2	-	2	-	2	-	2	-	2	-	2	-	1
**VY**	-	-	-	-	-	-	-	-	1	1	1	1	1	1	1	1	1	1	1	1	1	1	1	1	-	-
WD	-	-	-	-	-	-	-	-	-	1	-	-	-	-	-	-	-	-	-	-	-	-	-	-	-	-
WI	-	-	-	-	-	-	-	-	-	1	-	-	-	-	-	-	-	-	-	-	-	-	-	-	-	-
WM	-	-	-	-	-	-	-	-	-	-	-	1	-	1	-	1	-	1	-	1	-	1	-	1	-	-
WQ	-	-	-	-	-	-	-	-	-	-	-	-	-	-	-	-	-	-	-	-	-	-	-	-	-	1
WY	-	1	-	1	-	1	-	1	-	-	-	-	-	-	-	-	-	-	-	-	-	-	-	-	-	-
**YA**	-	-	-	-	-	-	-	-	-	-	-	-	-	-	-	-	-	-	-	-	-	-	-	-	1	1
YF	-	1	-	1	-	1	-	1	-	-	-	-	-	-	-	-	-	-	-	-	-	-	-	-	-	-
**YG**	-	-	-	-	-	-	-	-	-	-	-	-	-	-	-	-	-	-	-	-	-	-	-	-	1	1
YI	-	-	-	-	-	-	-	-	-	-	-	-	-	-	-	-	-	-	-	-	-	-	-	-	-	1
**YK**	1	1	1	1	1	1	1	1	1	1	-	-	-	-	-	-	-	-	-	-	-	-	-	-	-	-
**YL**	2	2	2	2	2	2	2	2	4	4	-	-	-	-	-	-	-	-	-	-	-	-	-	-	-	-
**YP**	2	2	2	2	2	2	2	2	-	-	3	3	3	3	3	3	3	3	3	3	3	3	3	3	2	2
YQ	-	1	-	1	-	1	-	1	-	5	-	1	-	1	-	1	-	1	-	1	-	1	-	1	-	1
YS	-	-	-	-	-	-	-	-	-	1	-	-	-	-	-	-	-	-	-	-	-	-	-	-	-	-
YT	-	1	-	1	-	1	-	1	-	-	-	-	-	-	-	-	-	-	-	-	-	-	-	-	-	-
YV	-	1	-	1	-	1	-	1	-	1	-	-	-	-	-	-	-	-	-	-	-	-	-	-	-	1
YY	-	1	-	1	-	1	-	1	-	-	-	-	-	-	-	-	-	-	-	-	-	-	-	-	-	2
AIP	-	-	-	-	-	-	-	-	-	-	-	-	-	-	-	-	-	-	-	-	-	-	-	-	1	-
AVP	-	-	-	-	-	-	-	-	1	-	1	-	1	-	1	-	1	-	1	-	1	-	1	-	-	-
FAL	-	-	-	-	-	-	-	-	1	-	-	-	-	-	-	-	-	-	-	-	-	-	-	-	-	-
FFL	-	-	-	-	-	-	-	-	-	-	-	-	-	-	-	-	-	-	-	-	-	-	-	-	1	-
FGK	1	-	1	-	1	-	1	-	-	-	-	-	-	-	-	-	-	-	-	-	-	-	-	-	-	-
GEP	-	-	-	-	-	-	-	-	-	-	-	-	-	-	-	-	-	-	-	-	-	-	-	-	1	-
GLP	1	-	1	-	1	-	1	-	-	-	-	-	-	-	-	-	-	-	-	-	-	-	-	-	-	-

GPV	-	-	-	-	-	-	-	-	-	-	1	-	1	-	1	-	1	-	1	-	1	-	1	-	-	-
IAK	-	-	-	-	-	-	-	-	-	-	-	-	-	-	-	-	-	-	-	-	-	-	-	-	1	-
IPI	-	-	-	-	-	-	-	-	-	-	-	-	-	-	-	-	-	-	-	-	-	-	-	-	-	1
IPP	-	-	-	-	-	-	-	-	-	-	1	-	1	-	1	-	1	-	1	-	1	-	1	-	1	-
IPY	-	-	-	-	-	-	-	-	1	-	-	-	-	-	-	-	-	-	-	-	-	-	-	-	-	-
**IQP**	-	-	-	-	-	-	-	-	1	1	-	-	-	-	-	-	-	-	-	-	-	-	-	-	-	-
IQY	-	-	-	-	-	-	-	-	-	-	-	-	-	-	-	-	-	-	-	-	-	-	-	-	1	-
LAY	1	-	1	-	1	-	1	-	-	-	-	-	-	-	-	-	-	-	-	-	-	-	-	-	-	-
LGP	-	-	-	-	-	-	-	-	-	-	1	-	1	-	1	-	1	-	1	-	1	-	1	-	-	-
LLP	-	-	-	-	-	-	-	-	-	-	-	-	-	-	-	-	-	-	-	-	-	-	1	-	-	-
LNF	-	-	-	-	-	-	-	-	1	-	-	-	-	-	-	-	-	-	-	-	-	-	-	-	-	-
LNP	-	-	-	-	-	-	-	-	1	-	-	-	-	-	-	-	-	-	-	-	-	-	-	-	-	-
LPL	-	-	-	-	-	-	-	-	-	-	-	-	-	-	-	-	-	2	-	-	-	-	-	-	-	-
LPP	-	-	-	-	-	-	-	-	-	-	2	-	2	-	2	-	1	-	2	-	2	-	1	-	-	-
**LQP**	-	-	-	-	-	-	-	-	-	-	1	1	1	1	1	1	1	1	1	1	1	1	1	1	-	-
LVY	-	-	-	-	-	-	-	-	-	-	1	-	1	-	1	-	1	-	1	-	1	-	1	-	-	-
PLG	1	-	1	-	1	-	1	-	-	-	-	-	-	-	-	-	-	-	-	-	-	-	-	-	-	-
PLP	-	-	-	-	-	-	-	-	-	-	1	-	1	-	1	-	2	-	1	-	1	-	-	-	-	-
PLW	1	-	1	-	1	-	1	-	-	-	-	-	-	-	-	-	-	-	-	-	-	-	-	-	-	-
PPK	-	-	-	-	-	-	-	-	-	-	-	-	-	-	-	-	-	-	-	-	-	-	-	-	1	-
PPL	-	-	-	-	-	-	-	-	-	-	-	2	-	2	-	2	-	1	-	2	-	2	-	2	-	-
PQR	-	-	-	-	-	-	-	-	-	-	1	-	1	-	1	-	1	-	1	-	1	-	1	-	-	-
PSY	-	-	-	-	-	-	-	-	-	-	-	-	-	-	-	-	-	-	-	-	-	-	-	-	1	-
PYP	-	-	-	-	-	-	-	-	-	-	1	-	1	-	1	-	1	-	1	-	1	-	1	-	1	-
RYQ	-	-	-	-	-	-	-	-	1	-	-	-	-	-	-	-	-	-	-	-	-	-	-	-	-	-
TVY	-	-	-	-	-	-	-	-	1	-	-	-	-	-	-	-	-	-	-	-	-	-	-	-	-	-
VAP	-	-	1	-	1	-	1	-	-	-	-	-	-	-	-	-	-	-	-	-	-	-	-	-	-	-
VAV	-	-	-	-	-	-	1	-	-	-	-	-	-	-	-	-	-	-	-	-	-	-	-	-	-	-
VLP	-	-	-	-	-	-	-	-	-	-	1	-	1	-	1	-	1	-	1	-	1	-	1	-	-	-
VPL	-	1	-	1	-	1	-	1	-	-	-	-	-	-	-	-	-	-	-	-	-	-	-	-	-	-
VPP	-	-	-	-	-	-	-	-	-	-	1	-	1	-	1	-	1	-	1	-	1	-	1	-	-	-
VRY	-	-	-	-	-	-	-	-	1	-	-	-	-	-	-	-	-	-	-	-	-	-	-	-	-	-
VYP	-	-	-	-	-	-	-	-	-	-	1	-	1	-	1	-	1	-	1	-	1	-	1	-	-	-

YGL	-	-	-	-	-	-	-	-	-	-	-	-	-	-	-	-	-	-	-	-	-	-	-	-	1	-
YPY	-	-	-	-	-	-	-	-	-	-	-	-	-	-	-	-	-	-	-	-	-	-	-	-	-	1
YVP	1	-	1	-	1	-	1	-	-	-	-	-	-	-	-	-	-	-	-	-	-	-	-	-	-	-
AFLL	-	-	-	-	-	-	-	-	-	-	1	-	1	-	1	-	1	-	1	-	1	-	1	-	-	-
AIPP	-	-	-	-	-	-	-	-	-	-	-	-	-	-	-	-	-	-	-	-	-	-	-	-	1	-
AYPS	1	-	1	-	1	-	1	-	-	-	-	-	-	-	-	-	-	-	-	-	-	-	-	-	-	-
FLQP	-	-	-	-	-	-	-	-	-	-	-	1	-	1	-	1	-	1	-	1	-	1	-	1	-	-
FVAP	-	-	1	-	1	-	1	-	-	-	-	-	-	-	-	-	-	-	-	-	-	-	-	-	-	-
GTQY	1	-	1	-	1	-	1	-	-	-	-	-	-	-	-	-	-	-	-	-	-	-	-	-	-	-
LFRQ	1	-	1	-	1	-	1	-	-	-	-	-	-	-	-	-	-	-	-	-	-	-	-	-	-	-
LHLP	-	-	-	-	-	-	-	-	-	-	1	-	1	-	1	-	1	-	1	-	1	-	1	-	-	-
LPLP	-	-	-	-	-	-	-	-	-	-	-	-	-	-	-	-	1	-	-	-	-	-	-	-	-	-
LQSW	-	-	-	-	-	-	-	-	-	-	1	-	1	-	1	-	1	-	1	-	1	-	1	-	-	-
LVYP	-	-	-	-	-	-	-	-	-	-	1	-	1	-	1	-	1	-	1	-	1	-	1	-	-	-
PFPE	-	-	-	-	-	-	1	-	-	-	-	-	-	-	-	-	-	-	-	-	-	-	-	-	-	-
RYLG	1	-	1	-	1	-	1	-	-	-	-	-	-	-	-	-	-	-	-	-	-	-	-	-	-	-
RYPS	-	-	-	-	-	-	-	-	-	-	-	-	-	-	-	-	-	-	-	-	-	-	-	-	1	-
VLGP	-	-	-	-	-	-	-	-	-	-	-	1	-	1	-	1	-	1	-	1	-	1	-	1	-	-
VRSP	-	-	-	-	-	-	-	-	-	-	-	-	-	-	-	-	-	-	-	-	-	-	-	-	1	-
VRYL	-	-	-	-	-	-	-	-	1	-	-	-	-	-	-	-	-	-	-	-	-	-	-	-	-	-
VVPP	-	-	-	-	-	-	-	-	-	-	1	-	1	-	1	-	1	-	1	-	1	-	1	-	-	-
WIQP	-	-	-	-	-	-	-	-	-	1	-	-	-	-	-	-	-	-	-	-	-	-	-	-	-	-
YAKP	-	-	-	-	-	-	-	-	-	-	-	-	-	-	-	-	-	-	-	-	-	-	-	-	1	-
YPYY	-	-	-	-	-	-	-	-	-	-	-	-	-	-	-	-	-	-	-	-	-	-	-	-	-	1
AMKPW	-	-	-	-	-	-	-	-	1	-	-	-	-	-	-	-	-	-	-	-	-	-	-	-	-	-
AVPYP	-	-	-	-	-	-	-	-	-	-	1	-	1	-	1	-	1	-	1	-	1	-	1	-	-	-
AYFYP	1	-	1	-	1	-	1	-	-	-	-	-	-	-	-	-	-	-	-	-	-	-	-	-	-	-
FFVAP	-	-	1	-	1	-	1	-	-	-	-	-	-	-	-	-	-	-	-	-	-	-	-	-	-	-
FPIIV	-	-	-	-	-	-	-	-	-	-	1	-	1	-	1	-	1	-	1	-	1	-	1	-	-	-
HLPLP	-	-	-	-	-	-	-	-	-	-	-	-	-	-	-	-	1	-	-	-	-	-	-	-	-	-
IPIQY	-	-	-	-	-	-	-	-	-	-	-	-	-	-	-	-	-	-	-	-	-	-	-	-	-	1
KDERF	-	-	-	-	-	-	-	-	-	-	-	-	-	-	-	-	-	-	-	-	-	-	-	-	1	-
LPLPL	-	-	-	-	-	-	-	-	-	-	-	-	-	-	-	-	-	1	-	-	-	-	-	-	-	-

**LPYPY**	-	-	-	-	-	-	-	-	-	-	-	-	-	-	-	-	-	-	-	-	-	-	-	-	1	1
NLHLP	-	-	-	-	-	-	-	-	-	-	1	-	1	-	1	-	1	-	1	-	1	-	1	-	-	-
PGPIP	-	-	-	-	-	-	-	-	-	-	-	-	1	-	-	-	1	-	-	-	1	-	-	-	-	-
RHPHP	-	-	-	-	-	-	-	-	-	-	-	-	-	-	-	-	-	-	-	-	-	-	-	-	1	-
RYLGY	1	-	1	-	1	-	1	-	-	-	-	-	-	-	-	-	-	-	-	-	-	-	-	-	-	-
RYQKF	-	-	-	-	-	-	-	-	1	-	-	-	-	-	-	-	-	-	-	-	-	-	-	-	-	-
SLVYP	-	-	-	-	-	-	-	-	-	-	1	-	1	-	1	-	1	-	1	-	1	-	1	-	-	-
SRYPS	-	-	-	-	-	-	-	-	-	-	-	-	-	-	-	-	-	-	-	-	-	-	-	-	1	-
TKVIP	-	-	-	-	-	-	-	-	1	-	-	-	-	-	-	-	-	-	-	-	-	-	-	-	-	-
YAKPA	-	-	-	-	-	-	-	-	-	-	-	-	-	-	-	-	-	-	-	-	-	-	-	-	1	-
ARHPHP	-	-	-	-	-	-	-	-	-	-	-	-	-	-	-	-	-	-	-	-	-	-	-	-	1	-
AYFYPE	1	-	1	-	1	-	1	-	-	-	-	-	-	-	-	-	-	-	-	-	-	-	-	-	-	-
DKIHPF	-	-	-	-	-	-	-	-	-	-	1	-	1	-	1	-	1	-	1	-	1	-	1	-	-	-
EKDERF	-	-	-	-	-	-	-	-	-	-	-	-	-	-	-	-	-	-	-	-	-	-	-	-	1	-
EMPFPK	-	-	-	-	-	-	-	-	-	-	1	-	1	-	1	-	1	-	1	-	1	-	1	-	-	-
FALPQY	-	-	-	-	-	-	-	-	1	-	-	-	-	-	-	-	-	-	-	-	-	-	-	-	-	-
KVLPVP	-	-	-	-	-	-	-	-	-	-	1	-	1	-	1	-	1	-	1	-	1	-	1	-	-	-
LAYFYP	1	-	1	-	1	-	1	-	-	-	-	-	-	-	-	-	-	-	-	-	-	-	-	-	-	-
LHLPLP	-	-	-	-	-	-	-	-	-	-	-	-	-	-	-	-	1	-	-	-	-	-	-	-	-	-
LKKISQ	-	-	-	-	-	-	-	-	1	-	-	-	-	-	-	-	-	-	-	-	-	-	-	-	-	-
PYVRYL	-	-	-	-	-	-	-	-	1	-	-	-	-	-	-	-	-	-	-	-	-	-	-	-	-	-
QSLVYP	-	-	-	-	-	-	-	-	-	-	1	-	1	-	1	-	1	-	1	-	1	-	1	-	-	-
TTMPLW	1	-	1	-	1	-	1	-	-	-	-	-	-	-	-	-	-	-	-	-	-	-	-	-	-	-
VPITPT	-	-	-	-	-	-	-	-	-	1	-	-	-	-	-	-	-	-	-	-	-	-	-	-	-	-
VTSTAV	-	-	-	-	-	-	-	-	-	-	-	-	-	-	-	-	-	-	-	-	-	-	-	-	1	-
VYPFPG	-	-	-	-	-	-	-	-	-	-	1	-	1	-	1	-	1	-	1	-	1	-	1	-	-	-
YFYPEL	1	-	1	-	1	-	1	-	-	-	-	-	-	-	-	-	-	-	-	-	-	-	-	-	-	-
YIPIQY	-	-	-	-	-	-	-	-	-	-	-	-	-	-	-	-	-	-	-	-	-	-	-	-	1	-
YKVPQL	1	-	1	-	1	-	1	-	-	-	-	-	-	-	-	-	-	-	-	-	-	-	-	-	-	-
YQEPVL	-	-	-	-	-	-	-	-	-	-	-	-	-	-	-	-	1	-	-	-	-	-	-	-	-	-
YQQPVL	-	-	-	-	-	-	-	-	-	-	1	-	1	-	1	-	-	-	1	-	1	-	1	-	-	-
YYQQKP	-	-	-	-	-	-	-	-	-	-	-	-	-	-	-	-	-	-	-	-	-	-	-	-	1	-
AVPYPQR	-	-	-	-	-	-	-	-	-	-	1	-	1	-	1	-	1	-	1	-	1	-	1	-	-	-

AYFYPEL	1	-	1	-	1	-	1	-	-	-	-	-	-	-	-	-	-	-	-	-	-	-	-	-	-	-
FPEVFGK	-	-	-	-	-	-	1	-	-	-	-	-	-	-	-	-	-	-	-	-	-	-	-	-	-	-
FPGPIPN	-	-	-	-	-	-	-	-	-	-	-	-	-	1	-	-	-	1	-	-	-	1	-	-	-	-
FPQYLQY	-	-	-	-	-	-	-	-	1	-	-	-	-	-	-	-	-	-	-	-	-	-	-	-	-	-
FSDKIAK	-	-	-	-	-	-	-	-	-	-	-	-	-	-	-	-	-	-	-	-	-	-	-	-	1	-
GPFPIIV	-	-	-	-	-	-	-	-	-	-	1	-	1	-	1	-	1	-	1	-	1	-	1	-	-	-
KVLPVPQ	-	-	-	-	-	-	-	-	-	-	-	-	-	-	-	-	1	-	-	-	-	-	-	-	-	-
LHLPLPL	-	-	-	-	-	-	-	-	-	-	-	-	-	-	-	-	1	-	-	-	-	-	-	-	-	-
LLYQQPV	-	-	-	-	-	-	-	-	-	-	1	-	1	-	1	-	-	-	1	-	1	-	1	-	-	-
LPQNIPP	-	-	-	-	-	-	-	-	-	-	-	1	-	1	-	1	-	1	-	1	-	1	-	1	-	-
LTLTDVE	-	-	-	-	-	-	-	-	-	-	1	-	1	-	1	-	1	-	1	-	1	-	1	-	-	-
NLHLPLP	-	-	-	-	-	-	-	-	-	-	-	-	-	-	-	-	1	-	-	-	-	-	-	-	-	-
TESQSLT	-	-	-	-	-	-	-	-	-	-	1	-	1	-	1	-	-	-	1	-	1	-	1	-	-	-
TQSLVYP	-	-	-	-	-	-	-	-	-	-	1	-	1	-	1	-	1	-	1	-	1	-	1	-	-	-
YPFPGPI	-	-	-	-	-	-	-	-	-	-	1	-	1	-	1	-	1	-	1	-	1	-	1	-	-	-
AYFYPELF	1	-	1	-	1	-	1	-	-	-	-	-	-	-	-	-	-	-	-	-	-	-	-	-	-	-
DAYPSGAW	1	-	1	-	1	-	1	-	-	-	-	-	-	-	-	-	-	-	-	-	-	-	-	-	-	-
ENLHLPLP	-	-	-	-	-	-	-	-	-	-	-	-	-	-	-	-	1	-	-	-	-	-	-	-	-	-
FALPQYLK	-	-	-	-	-	-	-	-	1	-	-	-	-	-	-	-	-	-	-	-	-	-	-	-	-	-
MKPWIQPK	-	-	-	-	-	-	-	-	1	-	-	-	-	-	-	-	-	-	-	-	-	-	-	-	-	-
NMAINPSK	-	-	-	-	-	-	-	-	1	-	-	-	-	-	-	-	-	-	-	-	-	-	-	-	-	-
QTQSLVYP	-	-	-	-	-	-	-	-	-	-	1	-	1	-	1	-	1	-	1	-	1	-	1	-	-	-
RGPFPIIV	-	-	-	-	-	-	-	-	-	-	1	-	1	-	1	-	1	-	1	-	1	-	1	-	-	-
SKVLPVPE	-	-	-	-	-	-	-	-	-	-	1	-	1	-	1	-	-	-	1	-	1	-	1	-	-	-
VYPFPGPI	-	-	-	-	-	-	-	-	-	-	1	-	1	-	1	-	1	-	1	-	1	-	1	-	-	-
YPFPGPIP	-	-	-	-	-	-	-	-	-	-	-	-	1	-	-	-	1	-	-	-	1	-	-	-	-	-
YPVQPFTE	-	-	-	-	-	-	-	-	-	-	1	-	1	-	1	-	-	-	1	-	1	-	1	-	-	-
ALNEINQFY	-	-	-	-	-	-	-	-	1	-	-	-	-	-	-	-	-	-	-	-	-	-	-	-	-	-
AQTQSLVYP	-	-	-	-	-	-	-	-	-	-	1	-	1	-	1	-	1	-	1	-	1	-	1	-	-	-
IPPLTQTPV	-	-	-	-	-	-	-	-	-	-	-	1	-	1	-	1	-	1	-	1	-	1	-	1	-	-
LNVPGEIVE	-	-	-	-	-	-	-	-	-	-	1	-	1	-	1	-	1	-	1	-	1	-	1	-	-	-
QSWMHQPHQ	-	-	-	-	-	-	-	-	-	-	1	-	1	-	1	-	1	-	1	-	1	-	1	-	-	-
RPKHPIKHQ	1	-	1	-	1	-	1	-	-	-	-	-	-	-	-	-	-	-	-	-	-	-	-	-	-	-

VRGPFPIIV	-	-	-	-	-	-	-	-	-	-	1	-	1	-	1	-	1	-	1	-	1	-	1	-	-	-
YPFPGPIPN	-	-	-	-	-	-	-	-	-	-	-	-	1	-	-	-	1	-	-	-	1	-	-	-	-	-
YPQRDMPIQ	-	-	-	-	-	-	-	-	-	-	1	-	1	-	1	-	1	-	1	-	1	-	1	-	-	-
YYAKPAAVR	-	-	-	-	-	-	-	-	-	-	-	-	-	-	-	-	-	-	-	-	-	-	-	-	1	-
FAQTQSLVYP	-	-	-	-	-	-	-	-	-	-	1	-	1	-	1	-	1	-	1	-	1	-	1	-	-	-
GPVRGPFPII	-	-	-	-	-	-	-	-	-	-	1	-	1	-	1	-	1	-	1	-	1	-	1	-	-	-
NIPPLTQTPV	-	-	-	-	-	-	-	-	-	-	1	-	1	-	1	-	1	-	1	-	1	-	1	-	-	-
YIPIQYVLSR	-	-	-	-	-	-	-	-	-	-	-	-	-	-	-	-	-	-	-	-	-	-	-	-	1	-
YQQPVLGPVR	-	-	-	-	-	-	-	-	-	-	1	-	1	-	1	-	-	-	1	-	1	-	1	-	-	-
ALNEINQFYQK	-	-	-	-	-	-	-	-	1	-	-	-	-	-	-	-	-	-	-	-	-	-	-	-	-	-
TPVVVPPFLQP	-	-	-	-	-	-	-	-	-	-	1	-	1	-	1	-	1	-	1	-	1	-	1	-	-	-
FFVAPFPEVFGK	-	-	-	-	-	-	1	-	-	-	-	-	-	-	-	-	-	-	-	-	-	-	-	-	-	-
HPFAQTQSLVYP	-	-	-	-	-	-	-	-	-	-	1	-	1	-	1	-	1	-	1	-	1	-	1	-	-	-
IHPFAQTQSLVYP	-	-	-	-	-	-	-	-	-	-	1	-	1	-	1	-	1	-	1	-	1	-	1	-	-	-
KIHPFAQTQSLVYP	-	-	-	-	-	-	-	-	-	-	1	-	1	-	1	-	1	-	1	-	1	-	1	-	-	-
LVYPFPGPIPNSLPQ NIPP	-	-	-	-	-	-	-	-	-	-	-	-	1	-	-	-	1	-	-	-	1	-	-	-	-	-
Total number of peptides	93	114	100	122	101	121	107	132	88	154	120	166	125	168	120	165	129	169	121	167	126	170	118	166	80	147

[1]A – ACE inhibitor, [2]D - DPP-IV inhibitory, [3]**bold** – sequence with dual (i.e., ACE- and DPP-IV-inhibitory) activity

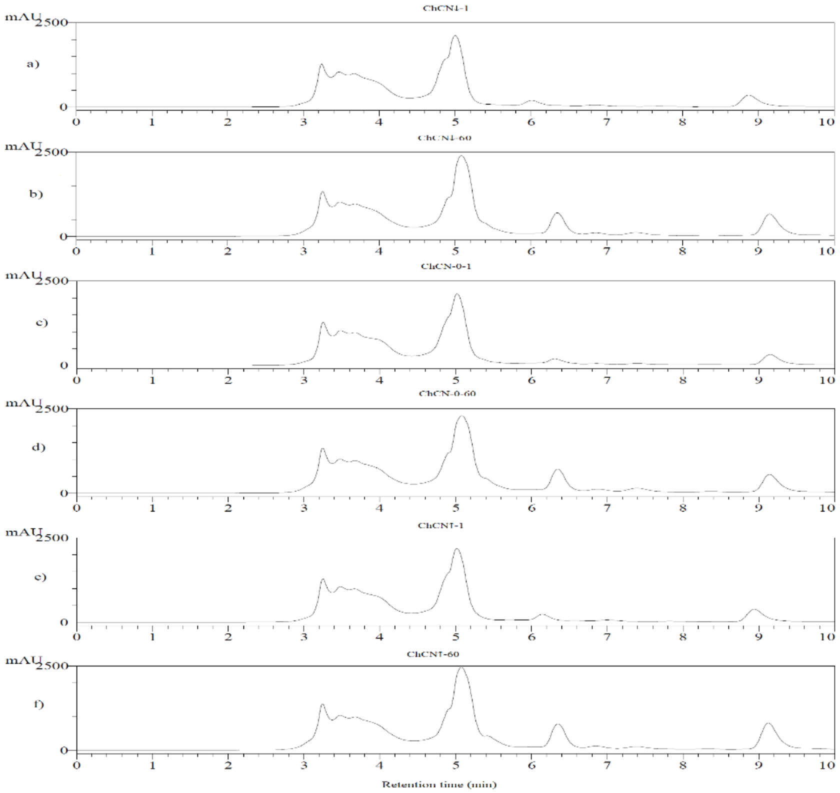

Figure S1. RP-HPLC chromatograms of water-soluble extracts derived from Gouda cheese with modified β-casein content before and after ripening (1st and 60th day, respectively), within the time interval 1-10 min. Abbreviations: ChCN↓-1 (a), ChCN↓-60 (b), ChCN-0-1 (c), ChCN-0-60 (d), ChCN↑-1 (e), ChCN↑-60 (f) – water-soluble extracts derived from Gouda cheese (Ch) with: reduced (↓), normative (0), and increased (↑) contents of

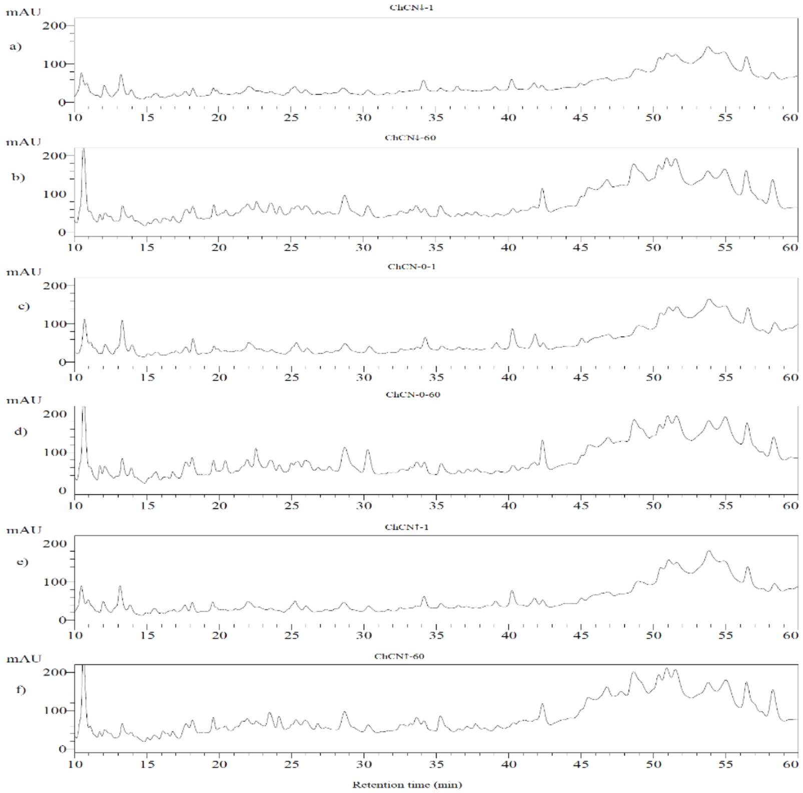

Figure S2. RP-HPLC chromatograms of water-soluble extracts derived from Gouda cheese with modified β-casein content before and after ripening (1st and 60th day, respectively), within the time interval 10-60 min. Abbreviations: ChCN↓-1 (a), ChCN↓-60 (b), ChCN-0-1 (c), ChCN-0-60 (d), ChCN↑-1 (e), ChCN↑-60 (f) – water-soluble extracts derived from Gouda cheese (Ch) with: reduced (↓), normative (0), and increased (↑) contents of

β-casein (CN) after 1 (suffix 1) and 60 (suffix 60) days of ripening, respectively (see Methods).

β-casein (CN) after 1 (suffix 1) and 60 (suffix 60) days of ripening, respectively (see Methods)

Table S2. RP-HPLC-MS/MS identification of peptides with ACE and/or DPP-IV-inhibitory activity in different Gouda cheese variants.

Sequence	t_R[1] (min)	m/z[2]	ChCN ↓-1[3]	ChCN ↓-60	ChCN 0- 1	ChCN -0-60	ChCN ↑-1	ChCN ↑-60	Casein[4]
AA^{dual5}	5.062	161.1	-	+	-	+	-	+ κ	κ^{A}
**PP**^{dual}	32.179	213.1	+	+	+	+	+	+	β^{all}, κ^{A}
**VV**$^{DPP\text{-}IVi6}$	15.946	217.2	+	+	+	+	+	+	α_{S2}^{A}, β^{all}, κA
FG^{ACEi7}	18.761	223.1	-	+	-	+	-	+	α_{S1}^{all}
**HA**$^{DPP\text{-}IVi}$	22.635	227.1	+	+	+	+	+	+	α_{S1}^{all}
HV$^{DPP\text{-}IVi}$	6.113	255.1	-	+	-	+	-	+	α_{S2}^{A}
**NK**^{ACEi}	20.370	261.2	+	+	+	+	+	+	β^{all}
TF^{dual}	14.969	267.1	-	+	-	+	-	+	α_{S2}^{A}
**ME**^{dual}	27.981	279.1	+	+	+	+	+	+	α_{S1}^{all}, α_{S2}^{A}
EH$^{DPP\text{-}IVi}$	15.555	285.1	-	+	-	+	-	+	α_{S2}^{A}
MH$^{DPP\text{-}IVi}$	18.512	287.1	-	+	-	+	-	+	β^{all}
**AIP**^{ACEi}	20.485	300.2	+	+	+	+	+	+	κ^{A}
IAK^{ACEi}	36.431	331.2	-	+	-	+	-	+	κ^{A}
**PPK**^{ACEi}	36.242	341.2	+	+	+	+	+	+	κ^{A}
**LNP**^{ACEi}	36.356	343.2	+	+	+	+	+	+	α_{S2}^{A}
FAL^{ACEi}	21.381	350.2	-	+	-	+	-	+	α_{S2}^{A}
FGK^{ACEi}	21.360	351.2	+	-	+	-	+	-	α_{S1}^{all}
LNF^{ACEi}	29.027	393.2	+	-	+	-	+	-	α_{S2}^{A}
AIPP^{ACEi}	16.044	397.2	+	-	+	-	-	+	κ^{A}
**PQR**^{ACEi}	25.706	400.2	+	+	+	+	+	+	β^{all}
FVAP^{ACEi}	25.270	433.2	+	+	+	+	+	+	$\alpha_{S1}^{B,C,D}$

LPLP^{ACEi}	29.105	439.3	-	+	-	+	-	+	βB
**GTQY**^{ACEi}	23.777	468.2	+	+	+	+	+	+	α_{S1}^{all}
PFPE^{ACEi}	22.262	489.2	-	+	-	+	-	+	α_{S1}^{D}
RYLG^{ACEi}	18.715	508.3	-	+	-	+	-	-	α_{S1}^{all}
LQSW^{ACEi}	34.158	533.3	+	-	+	-	+	+	β^{all}
**WIQP**$^{DPP-IVi}$	22.460	543.3	+	+	+	+	+	+	α_{S2}^{A}
**AVPYP**^{ACEi}	18.869	546.3	+	+	+	+	+	+	β^{all}
LPLPL$^{DPP-IVi}$	36.251	552.4	+	-	+	-	+	-	β^{B}
TKVIP^{ACEi}	22.863	557.4	+	-	+	-	+	-	α_{S2}^{A}
**LFRQ**^{ACEi}	21.897	563.3	+	+	+	+	+	+	α_{S1}^{all}
HLPLP^{ACEi}	26.979	576.3	+	-	+	-	+	-	β^{B}
FFVAP^{ACEi}	21.947	580.3	+	-	+	-	+	-	$\alpha_{S1}^{B,C,D}$
FPIIV^{ACEi}	20.820	588.4	-	+	-	+	-	+	β^{all}
**NLHLP**^{ACEi}	23.601	593.3	+	+	+	+	+	+	β^{all}
VPITPT$^{DPP-IVi}$	24.328	627.4	+	+	+	+	+	+	α_{S2}^{A}
**AMKPW**^{ACEi}	27.366	632.3	+	+	+	+	+	+	α_{S2}^{A}
**IPIQY**$^{DPP-IVi}$	29.073	633.3	+	+	+	+	+	+	κ^{A}
AYFYP^{ACEi}	24.033	660.3	-	+	-	+	-	+	α_{S1}^{all}
**RYLGY**^{ACEi}	28.930	671.3	+	+	+	+	+	+	α_{S1}^{all}
KDERF^{ACEi}	28.575	694.4	-	-	+	-	+	-	κ^{A}
ARHPHP^{ACEi}	52.565	714.4	+	-	+	-	+	-	κ^{A}
LKKISQ^{ACEi}	29.401	716.5	-	-	+	-	+	-	α_{S2}^{A}
GPFPIIV^{ACEi}	31.149	742.4	-	+	-	+	-	+	β^{all}
DKIHPF^{ACEi}	22.606	756.4	-	+	-	+	-	+	β^{all}
LAYFYP^{ACEi}	31.494	773.4	-	+	-	+	-	+	α_{S1}^{all}
LPQNIPP$^{DPP-IVi}$	25.077	778.4	-	+	-	+	-	+	β^{all}
**AYFYPE**^{ACEi}	17.108	789.3	+	+	+	+	+	+	α_{S1}^{all}

**LHLPLPL**^{ACEi}	18.087	802.5	+	+	+	+	+	+	β^{B}
NLHLPLP^{ACEi}	19.443	803.5	+	-	+	-	+	-	β^{B}
**FSDKIAK**^{ACEi}	21.871	808.5	+	+	+	+	+	+	κ^{A}
**AVPYPQR**^{ACEi}	24.422	830.4	+	+	+	+	+	+	β^{all}
**LLYQQPV**^{ACEi}	26.657	860.5	+	+	+	+	+	+	$\beta^{A1, A2, A3, C, E, F}$
DAYPSGAW^{ACEi}	29.459	866.3	-	+	-	+	-	+	α_{S1}^{all}
**SKVLPVPE**^{ACEi}	25.869	868.5	+	+	+	+	+	+	$\beta^{A1, A2, A3, C, E, F}$
AYFYPEL^{ACEi}	18.075	902.4	-	+	-	+	-	+	α_{S1}^{all}
**ENLHLPLP**^{ACEi}	20.145	932.5	+	+	+	+	+	+	β^{B}
FPQYLQY^{ACEi}	22.559	958.5	+	-	+	-	+	-	α_{S2}^{A}
IPPLTQTPV$^{DPP-IVi}$	20.587	965.6	+	-	+	-	+	-	β^{all}
LNVPGEIVE^{ACEi}	20.910	969.5	+	-	+	-	+	-	β^{all}
AYFYPELF^{ACEi}	29.571	1049.5	+	-	+	-	+	-	α_{S1}^{all}
RPKHPIKHQ^{ACEi}	16.101	1140.7	-	+	-	+	-	+	α_{S1}^{all}
YPQRDMPIQ^{ACEi}	32.747	1147.5	-	+	-	+	-	+	β^{all}

[1]t_R – retention time (min), [2]all precursor ions of identified peptide are $(M+H)^+$; [3]ChCN↓-1, ChCN↓-60, ChCN-0-1, ChCN-0-60, ChCN↑-1, ChCN↑-60 – water-soluble extracts derived from Gouda cheese with: reduced (↓), normative (0), and increased (↑) content of β-casein after 1st (suffix 1) and 60th (suffix 60) day of ripening, respectively,[4]letters assigned to the casein sources refer to their specific genetic variant, whereas term "all" means the presence of a peptide in all genetic variants of specific casein that had been *in silico* analyzed, [5]dual - ACE and DPP-IV inhibitor; [6]DPP-IVi - DPP-IV inhibitor; [7]ACEi - ACE inhibitor; **bold** - peptides identified in all cheese variants.

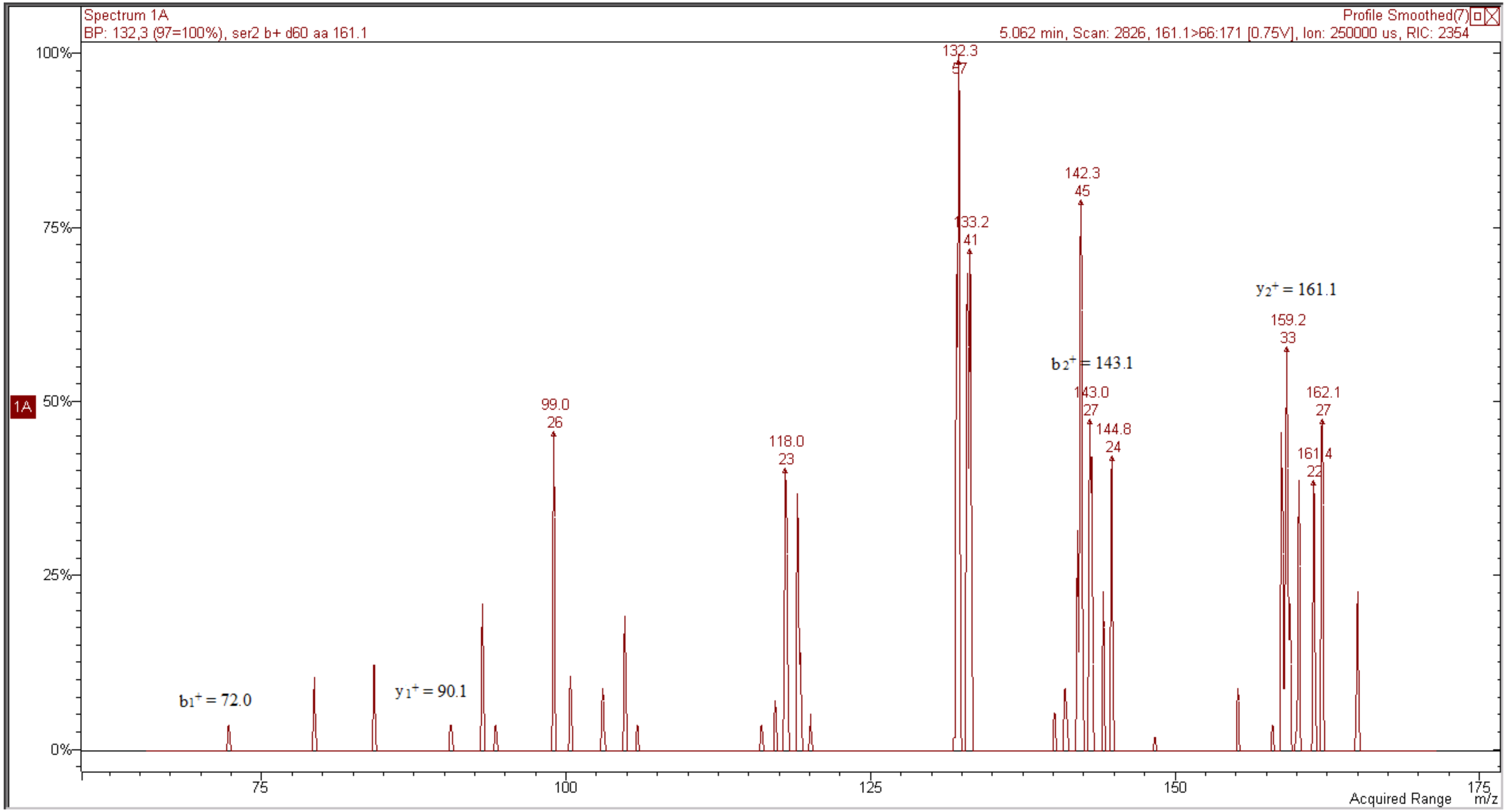

Figure S3. MS/MS spectrum of AA peptide.

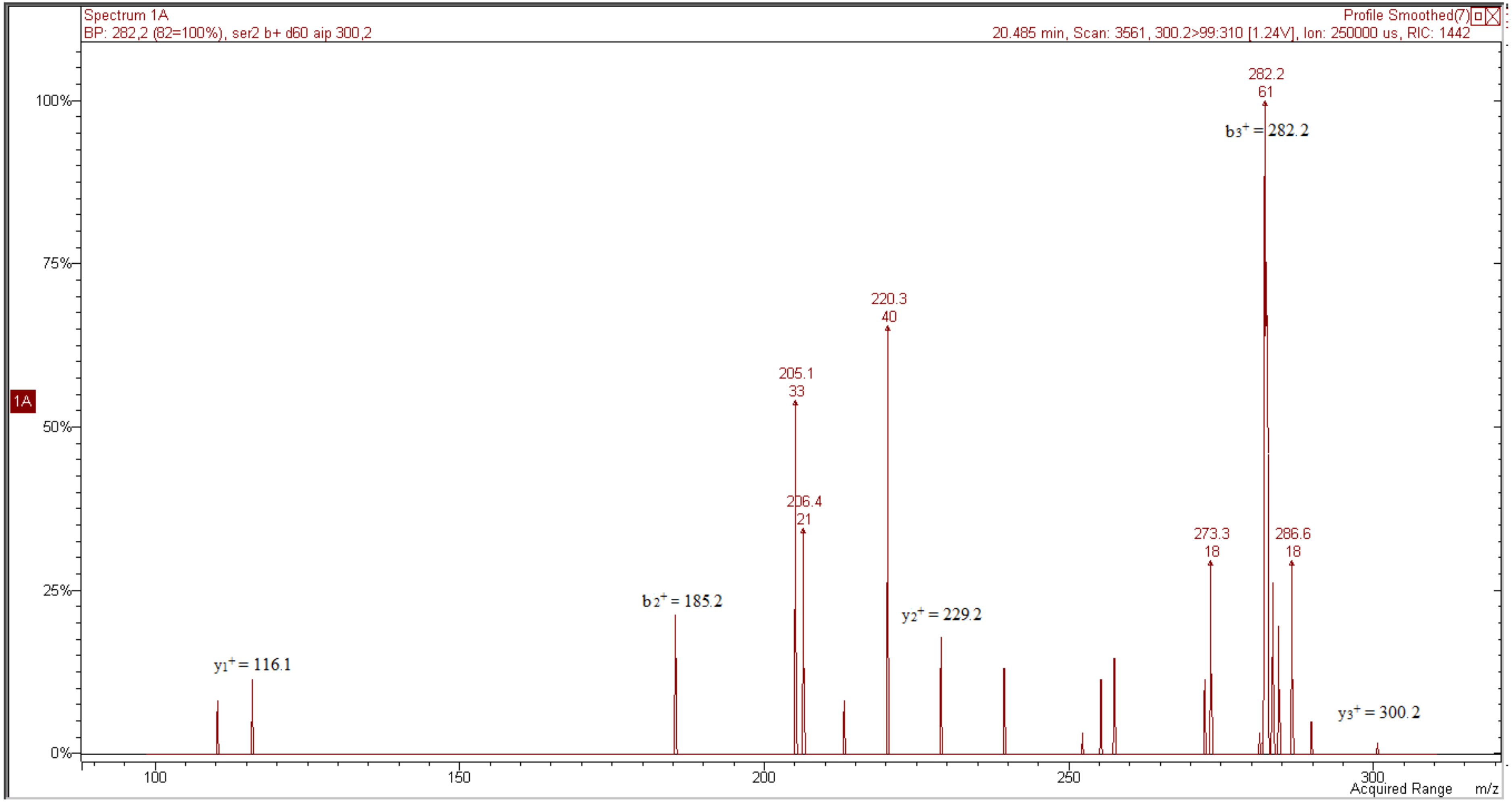

Figure S4. MS/MS spectrum of AIP peptide.

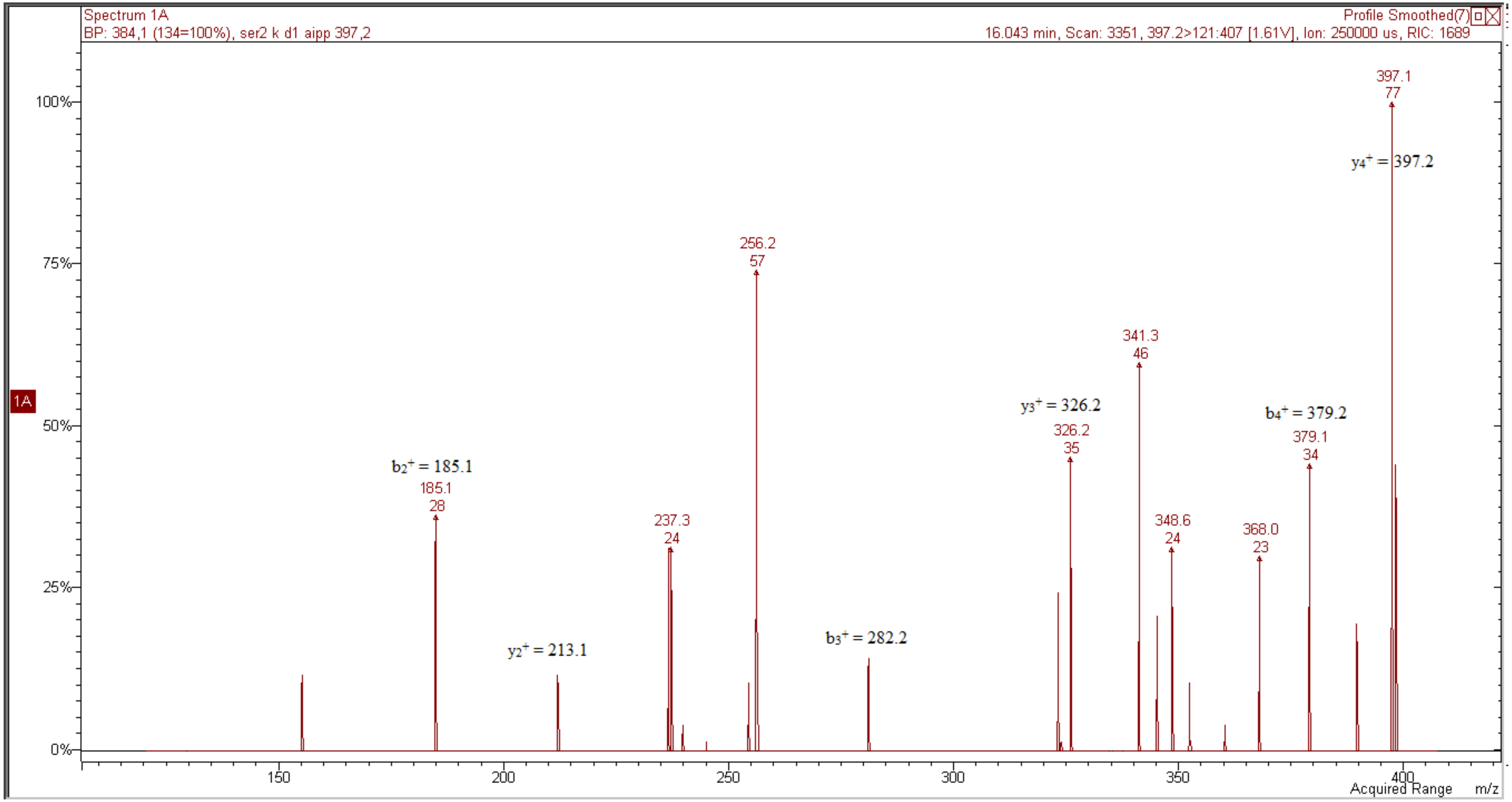

Figure S5. MS/MS spectrum of AIPP peptide.

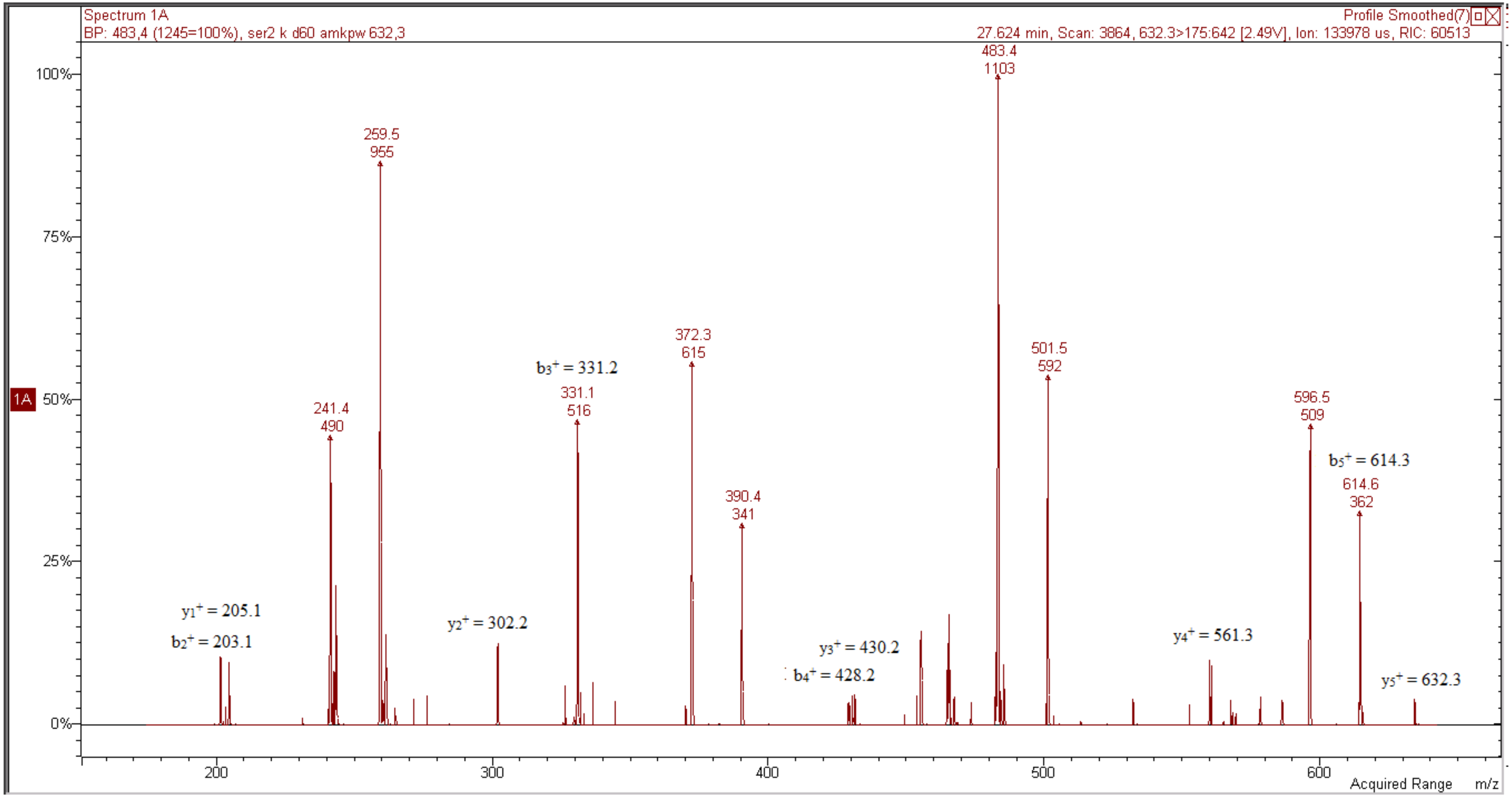

Figure S6. MS/MS spectrum of AMPKW peptide.

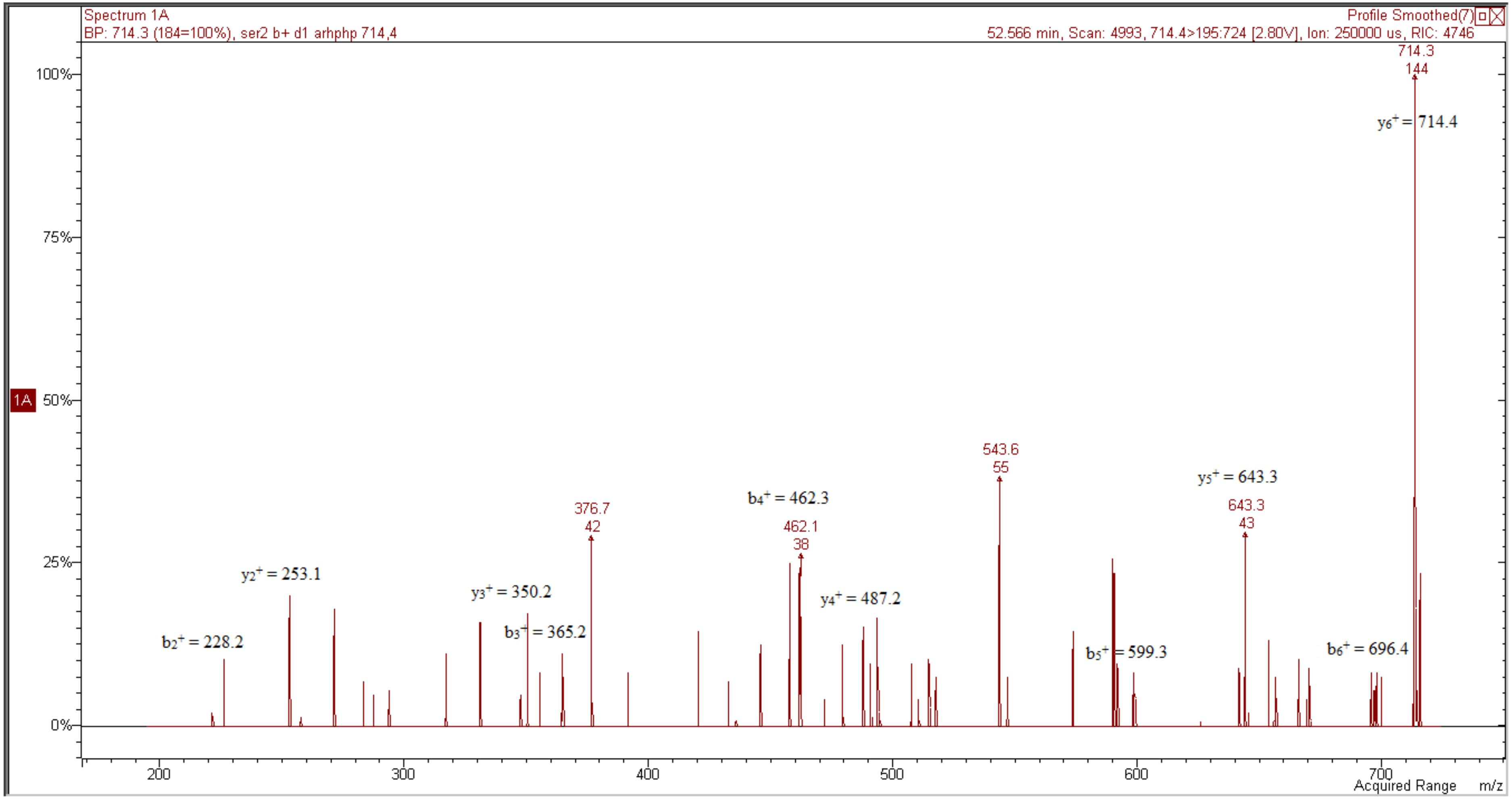

Figure S7. MS/MS spectrum of ARHPHP peptide.

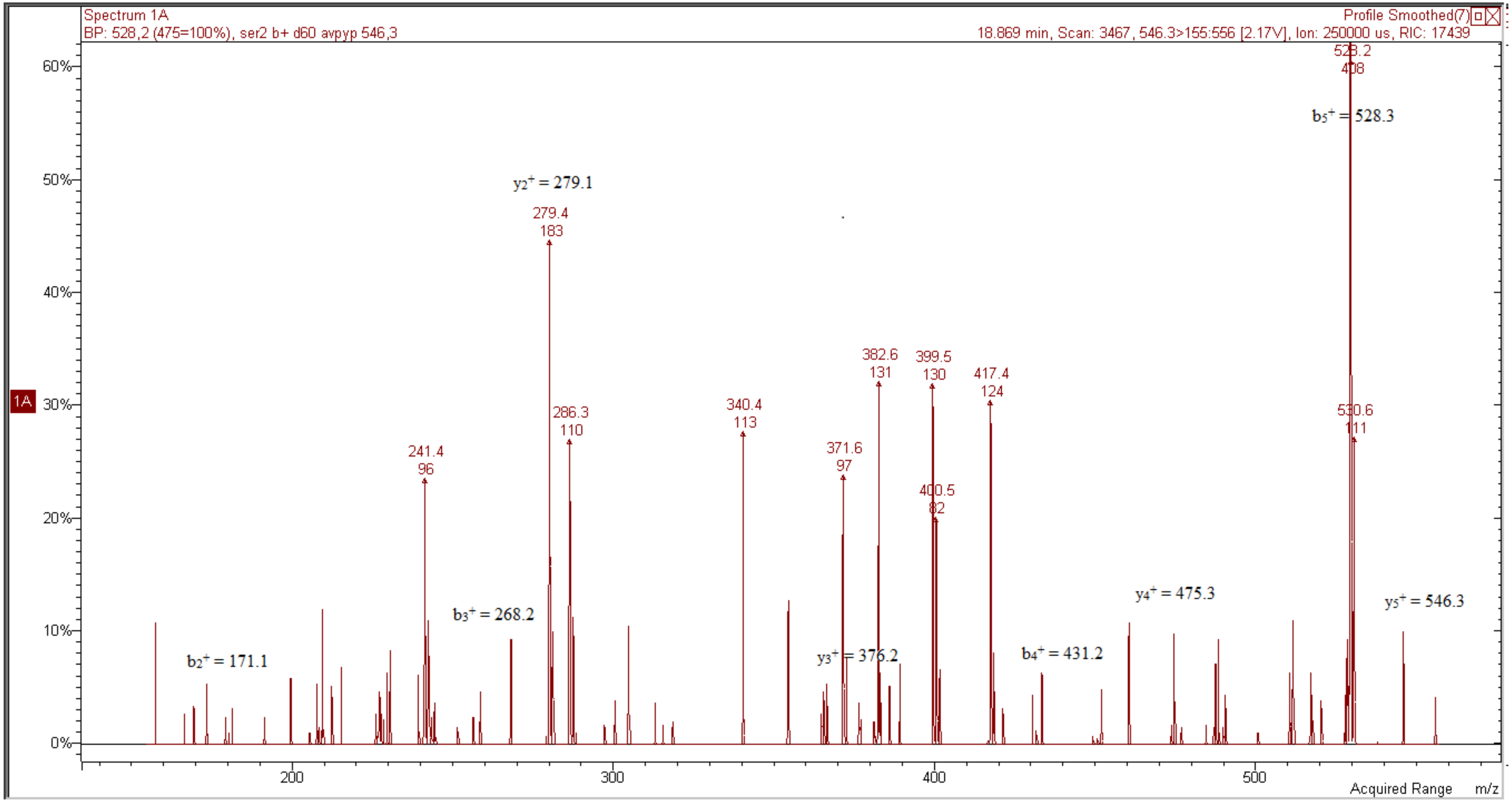

Figure S8. MS/MS spectrum of AVPYP peptide.

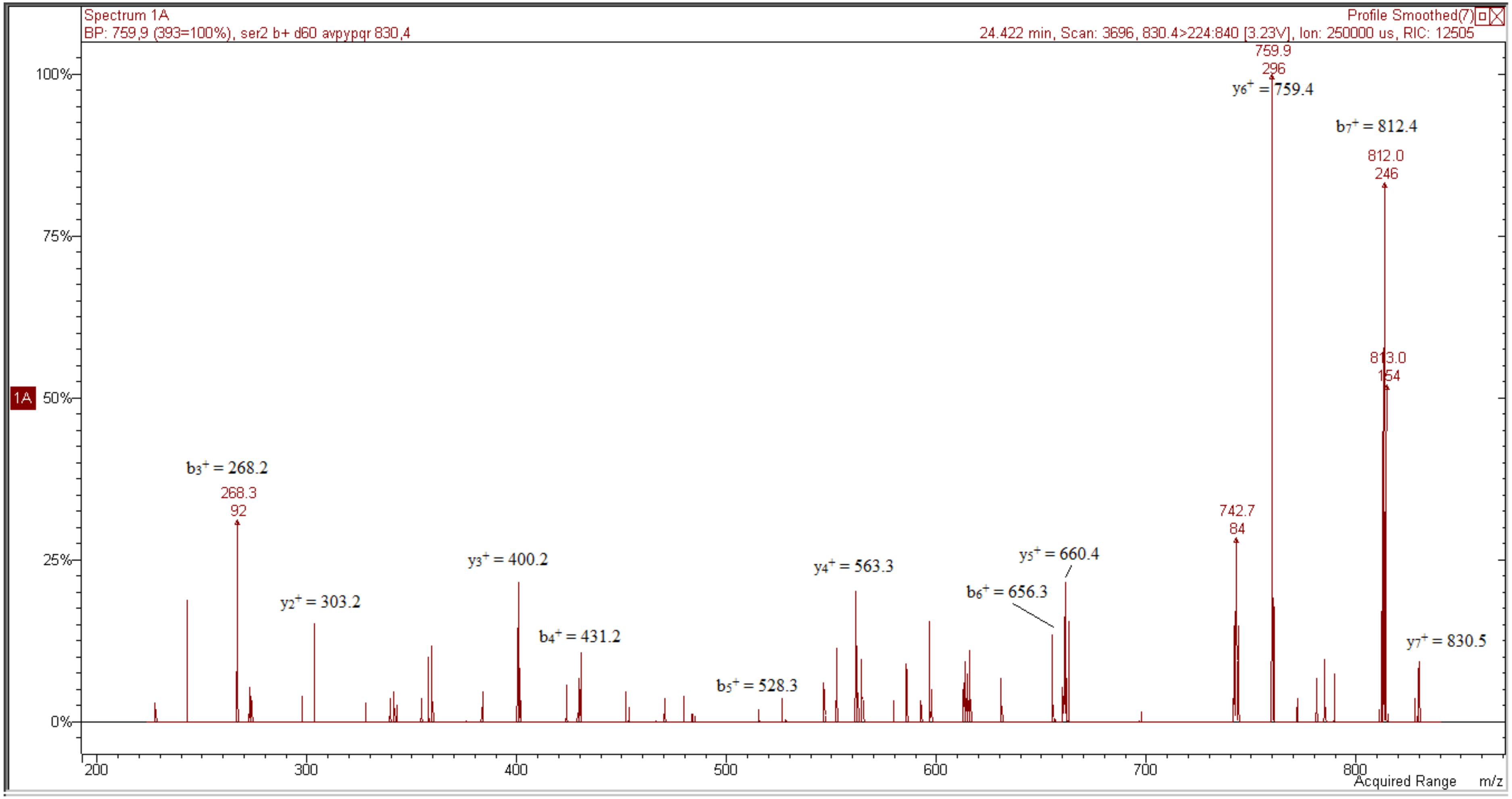

Figure S9. MS/MS spectrum of AVPYPQR peptide.

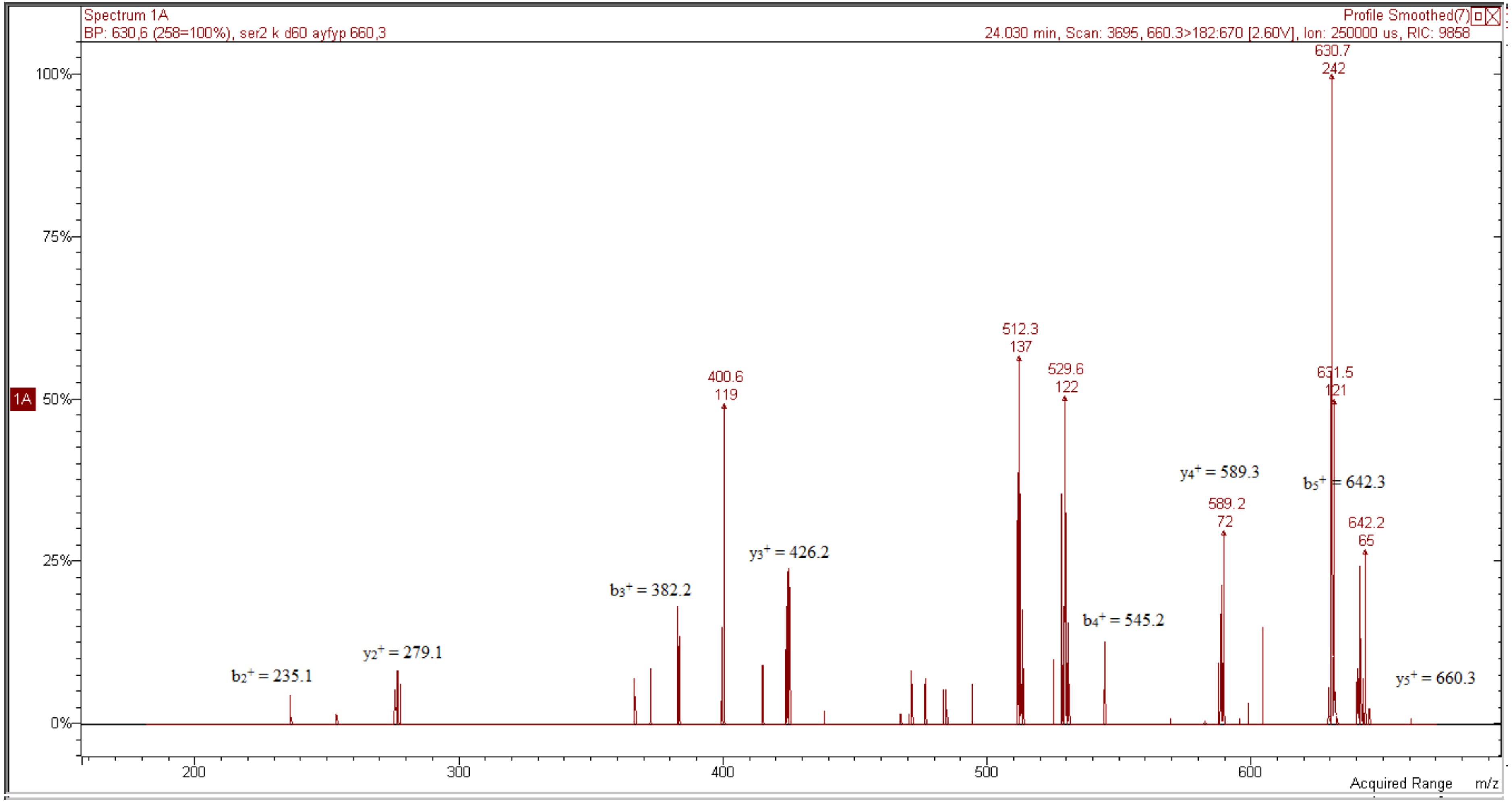

Figure S10. MS/MS spectrum of AYFYP peptide.

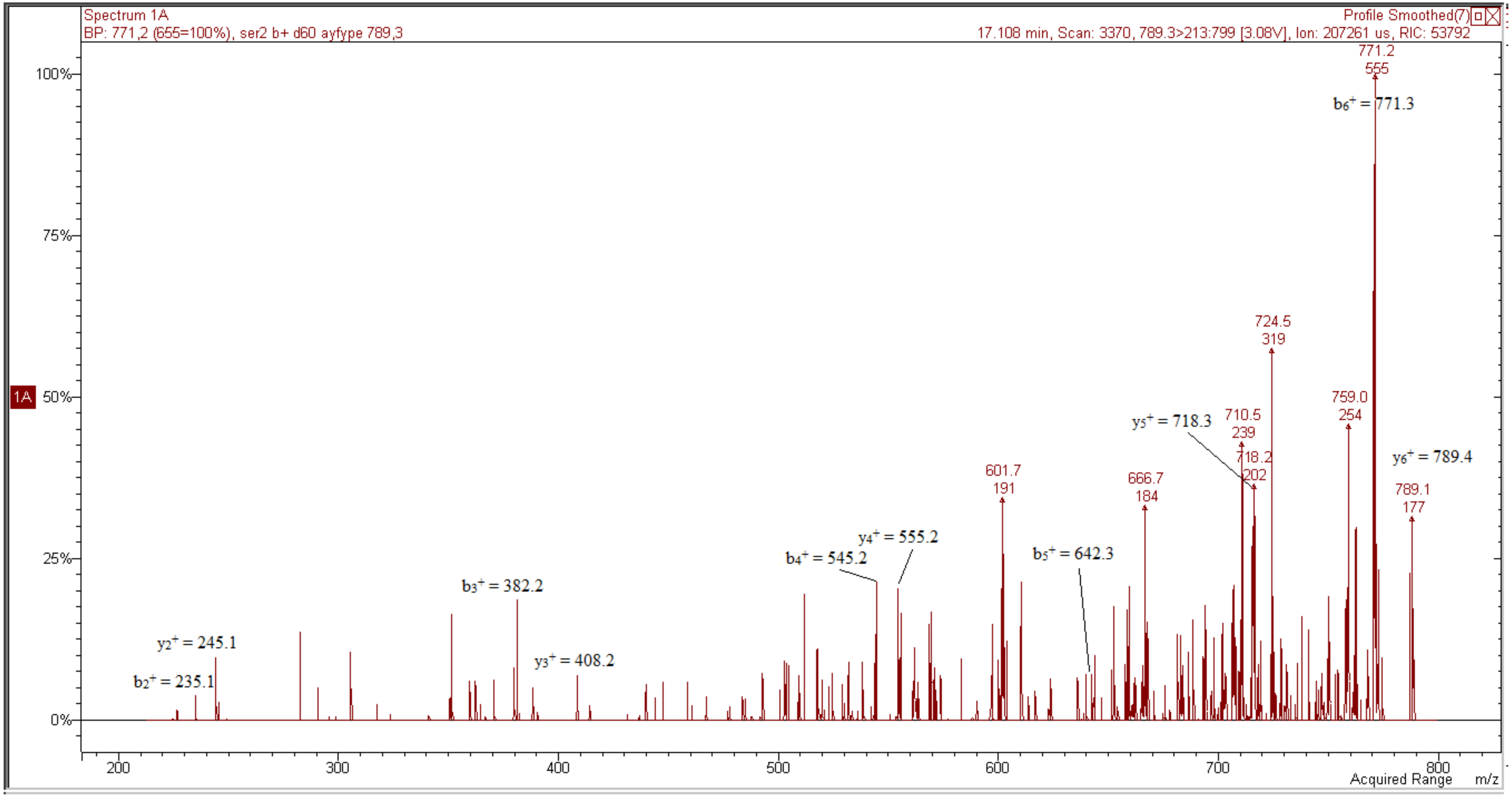

Figure S11. MS/MS spectrum of AYFYPE peptide.

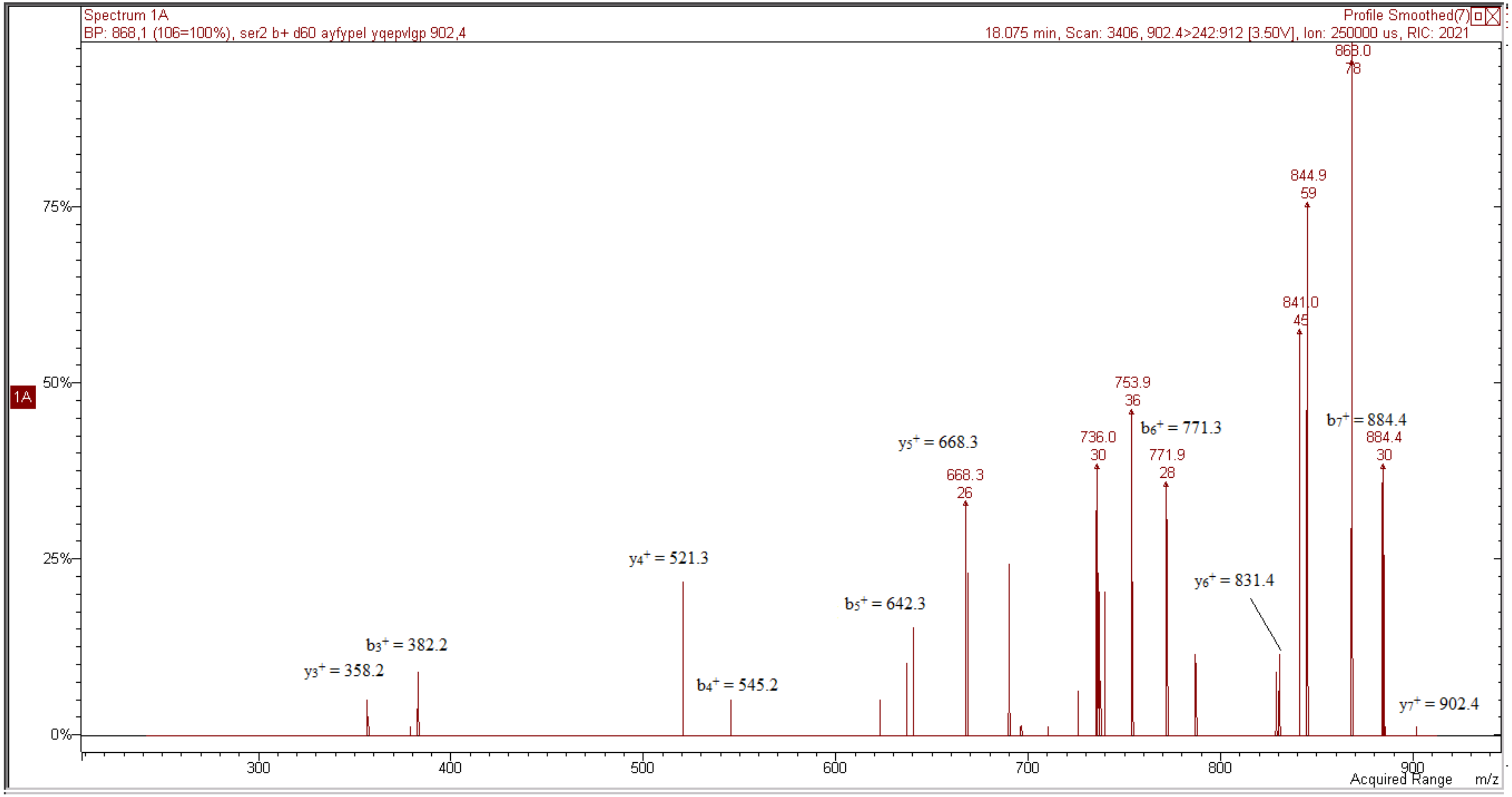

Figure S12. MS/MS spectrum of AYFYPEL peptide.

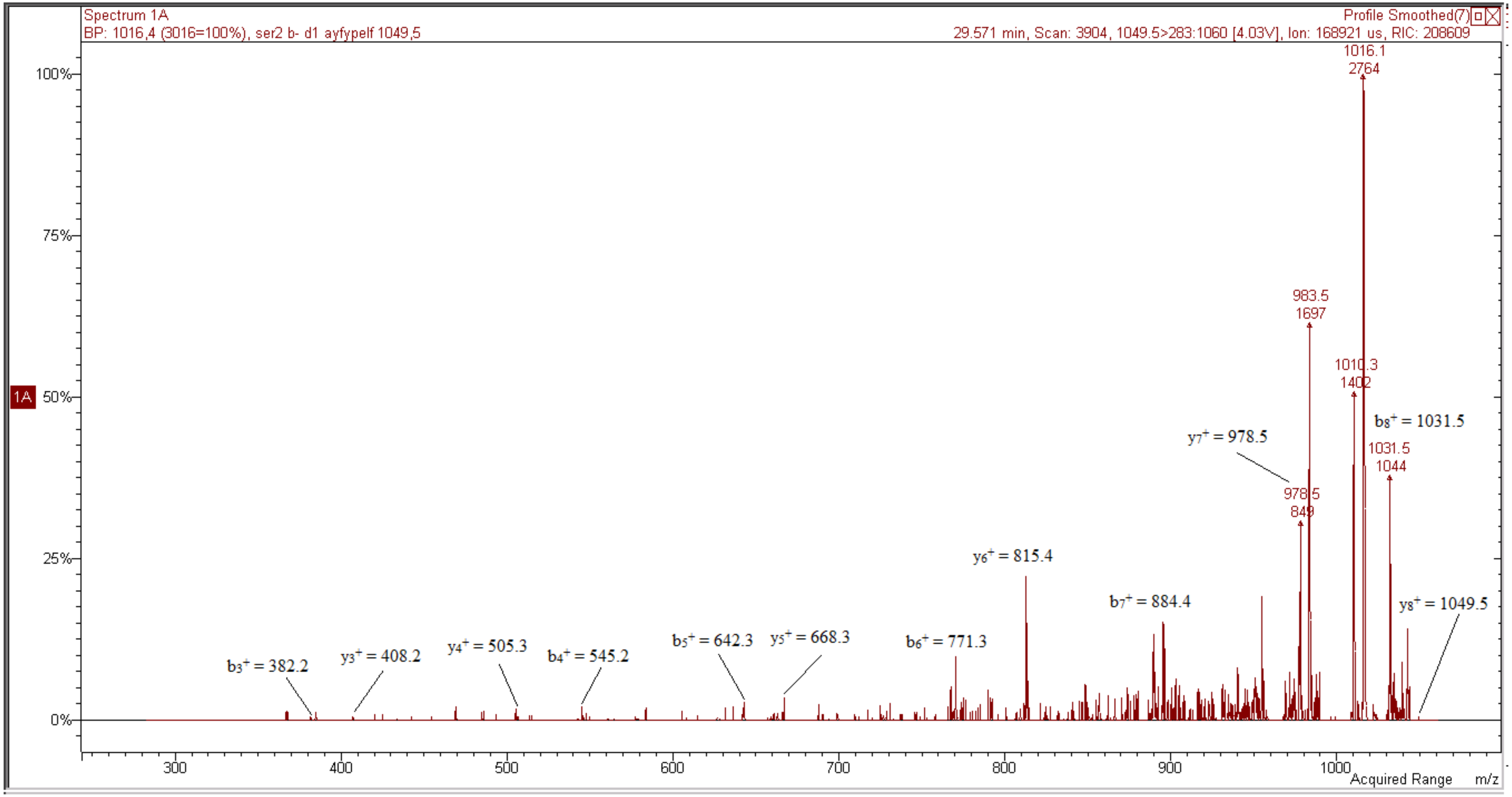

Figure S13. MS/MS spectrum of AYFYPELF peptide.

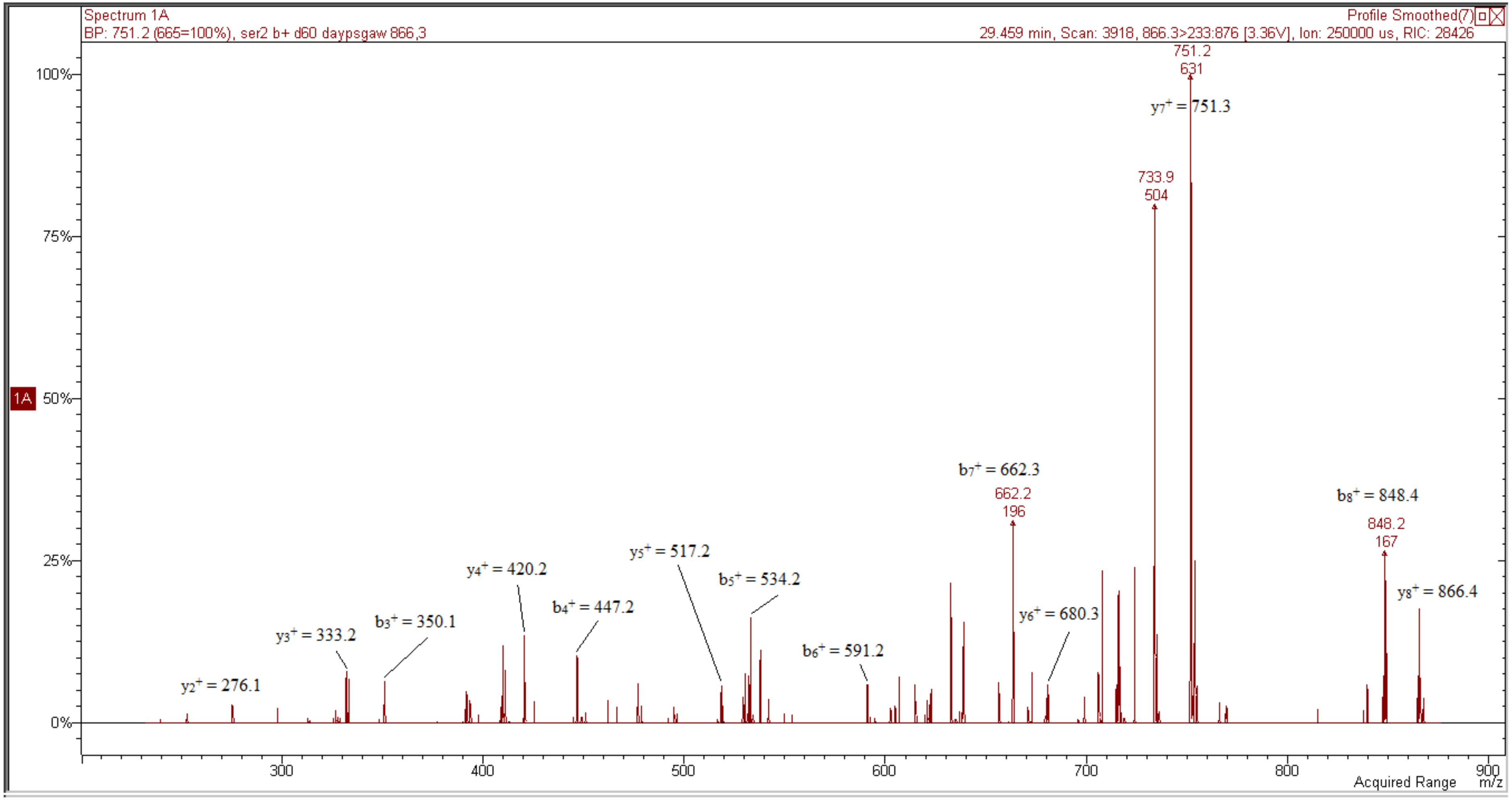

Figure S14. MS/MS spectrum of DAYPSGAW peptide.

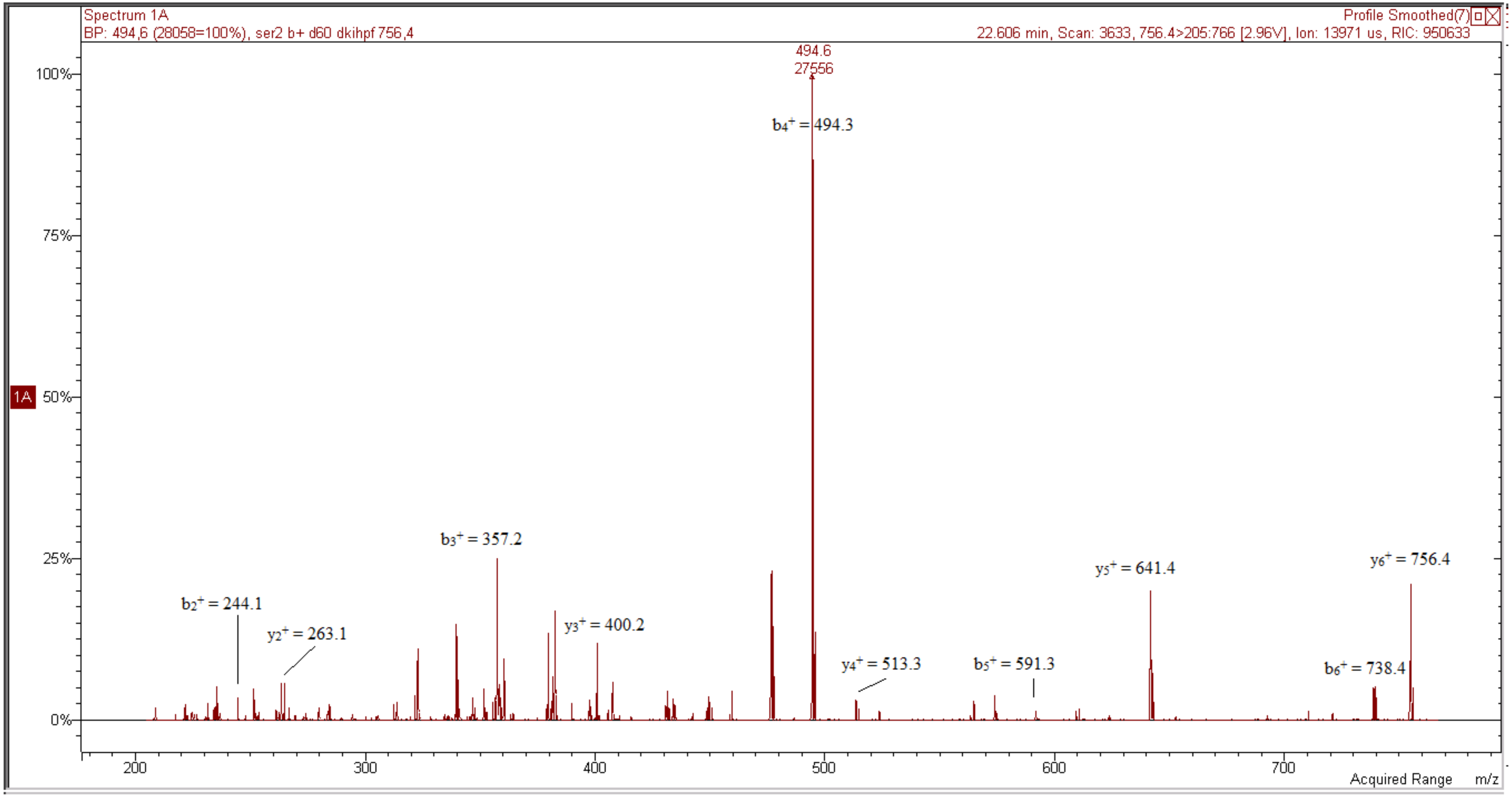

Figure S15. MS/MS spectrum of DKIHPF peptide.

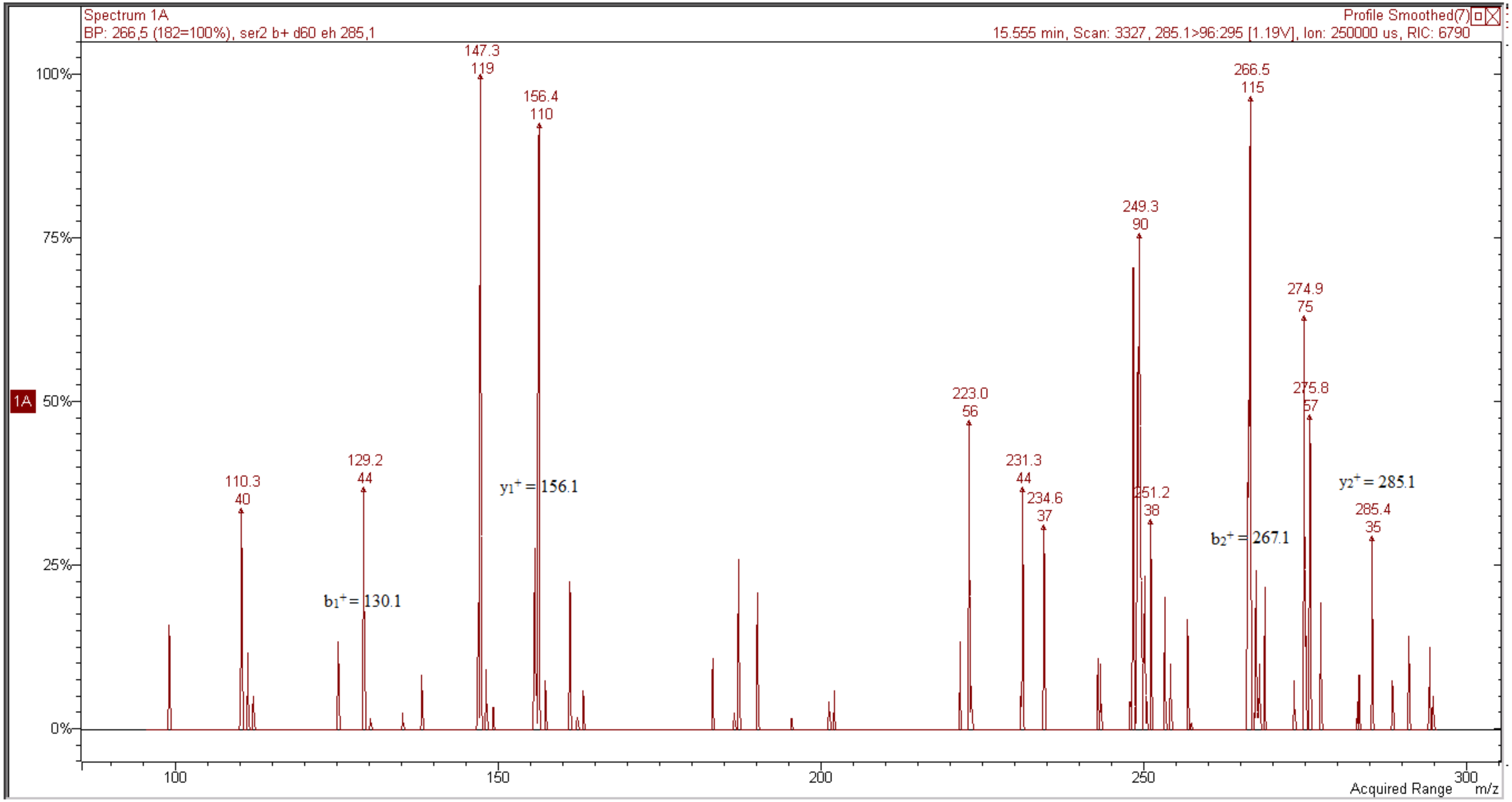

Figure S16. MS/MS spectrum of EH peptide.

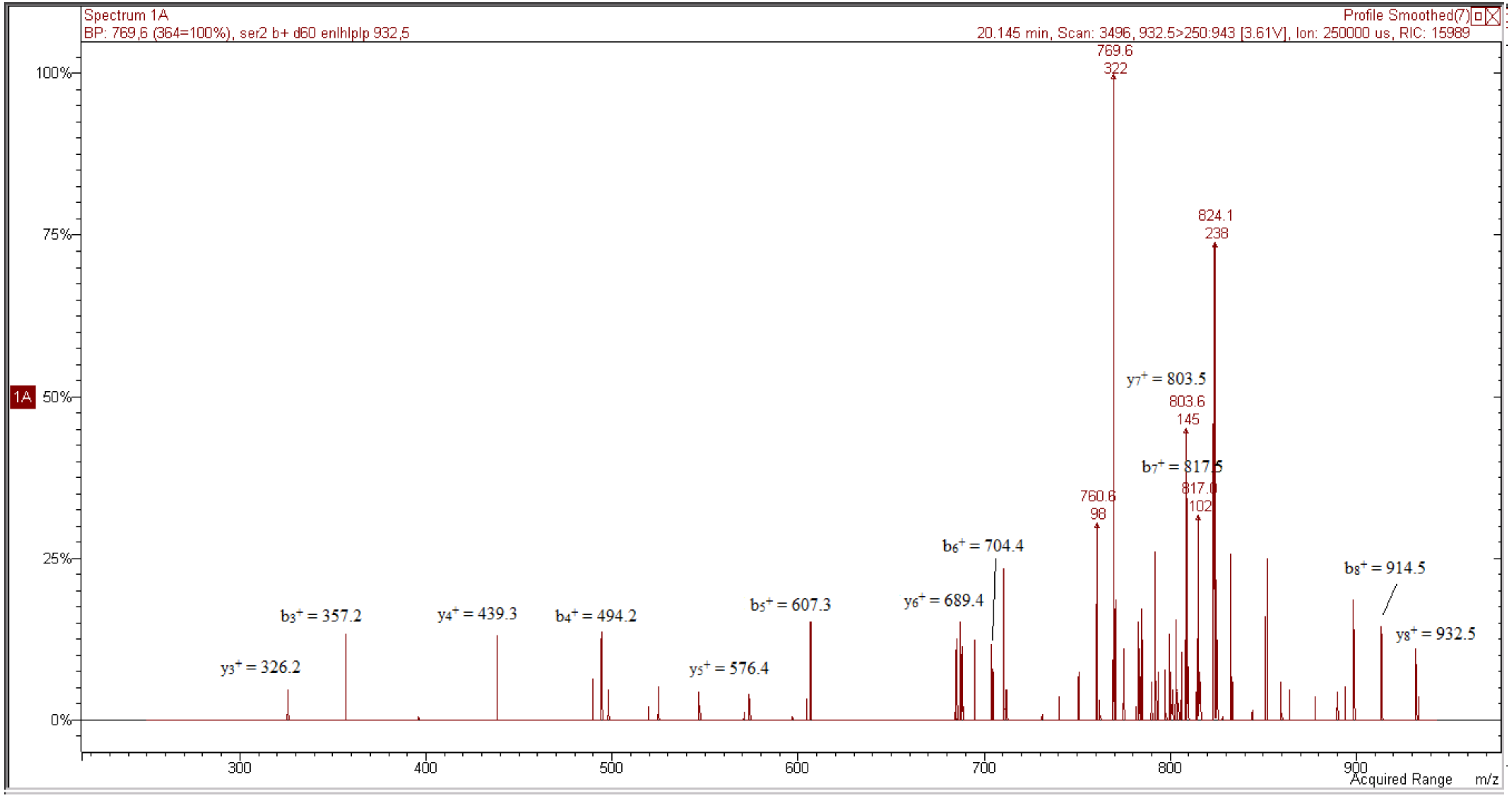

Figure S17. MS/MS spectrum of ENLHLPLP peptide.

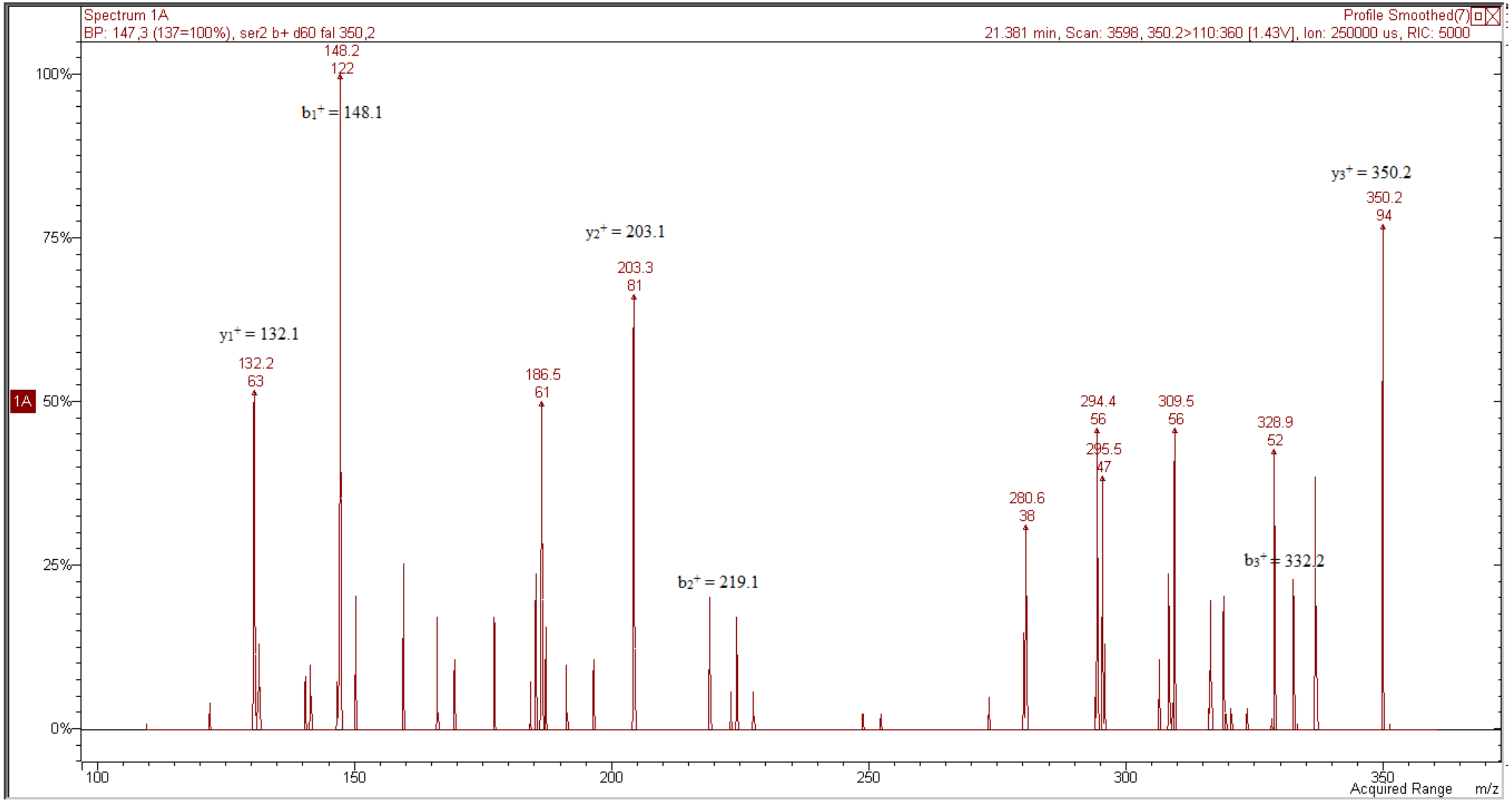

Figure S18. MS/MS spectrum of FAL peptide.

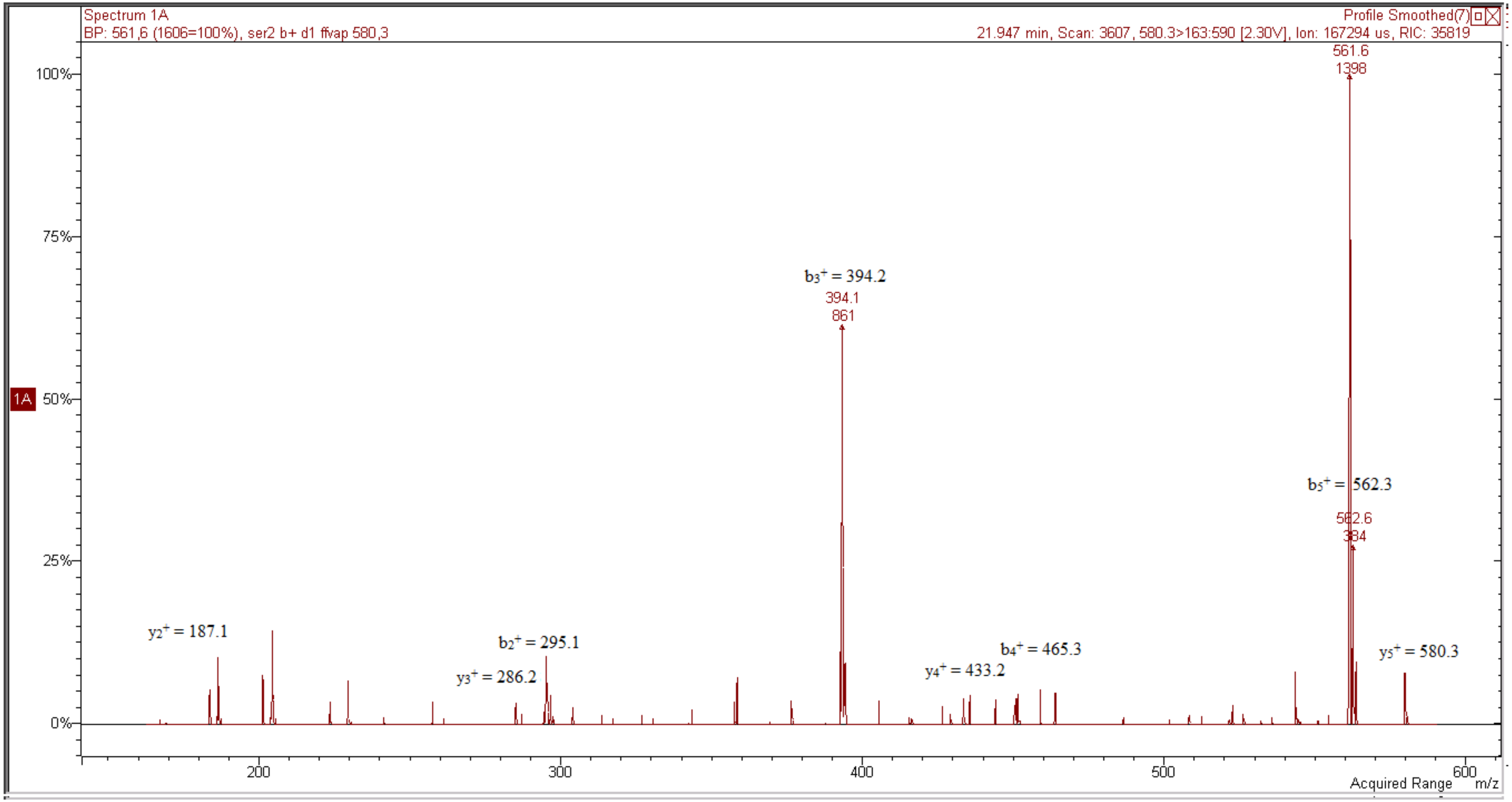

Figure S19. MS/MS spectrum of FFVAP peptide.

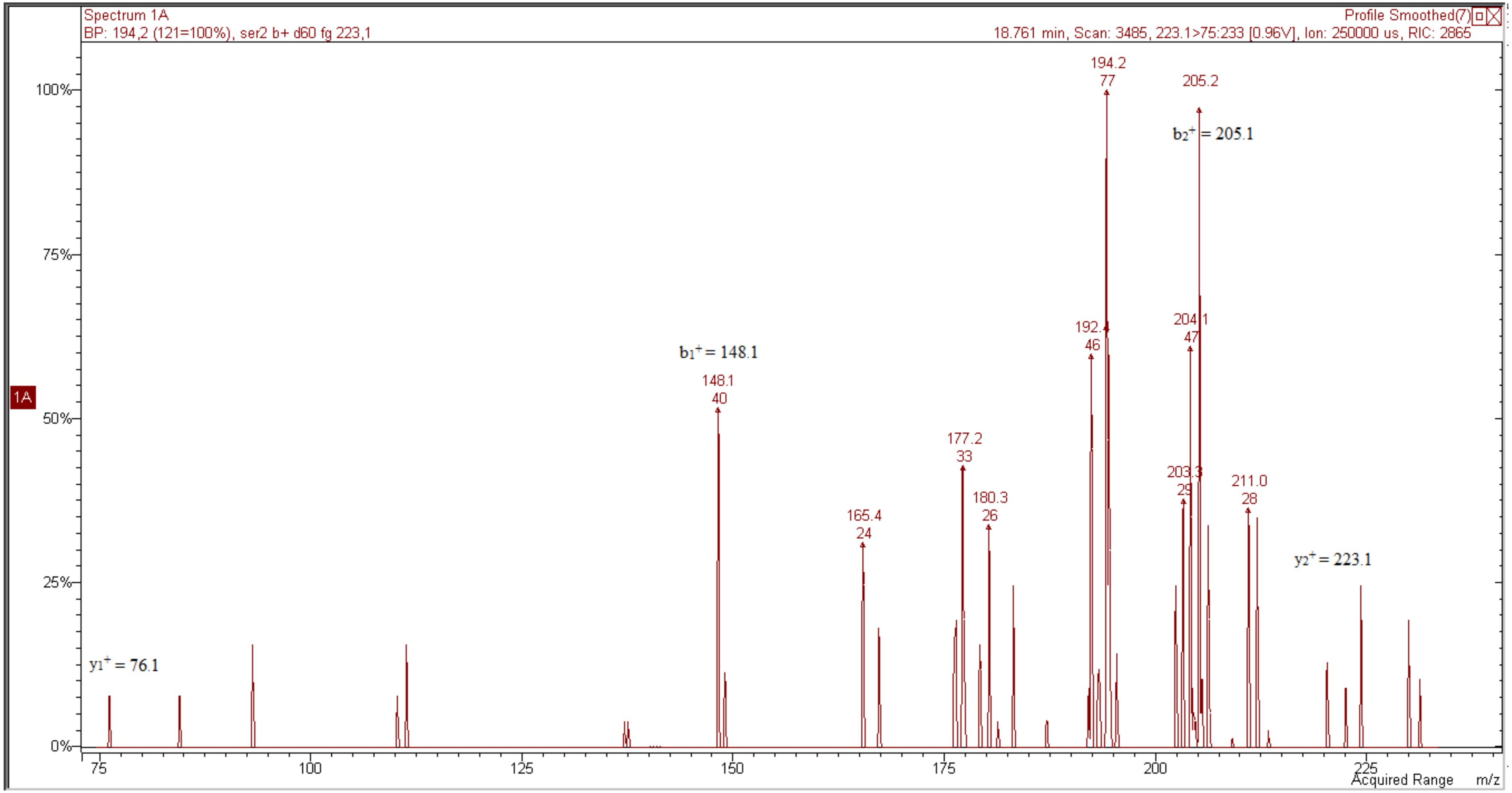

Figure S20. MS/MS spectrum of FG peptide.

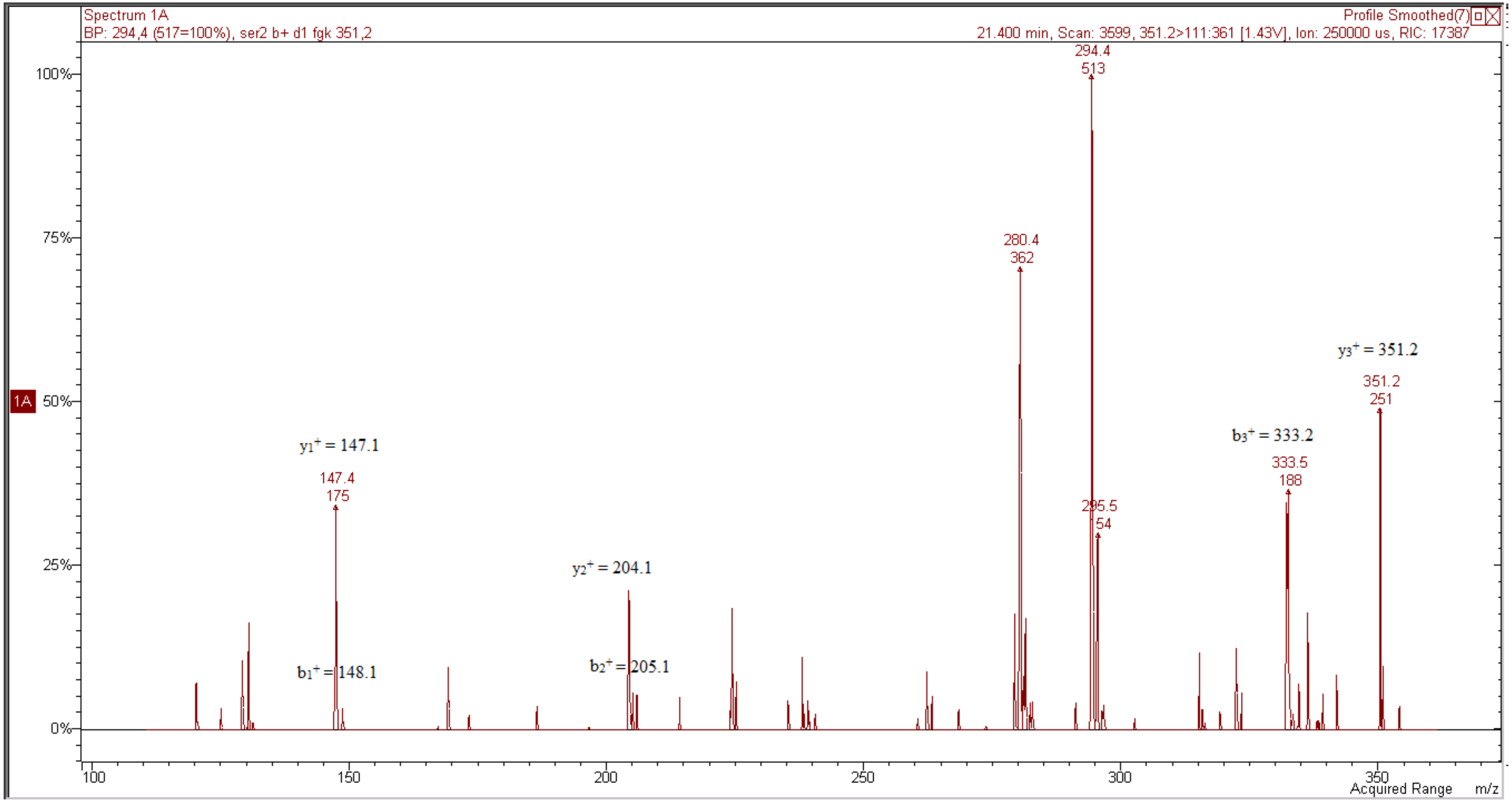

Figure S21. MS/MS spectrum of FGK peptide.

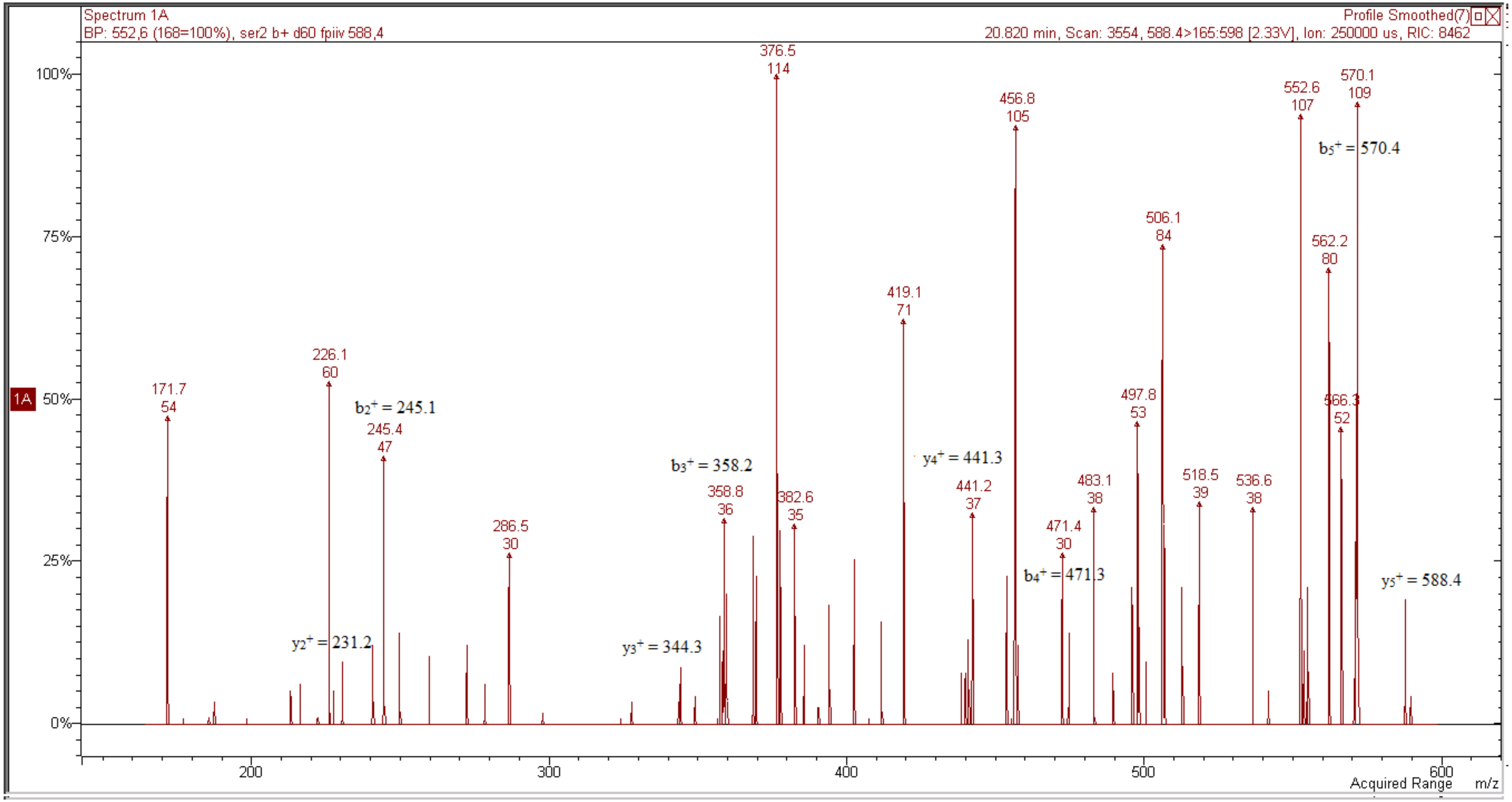

Figure S22. MS/MS spectrum of FPIIV peptide.

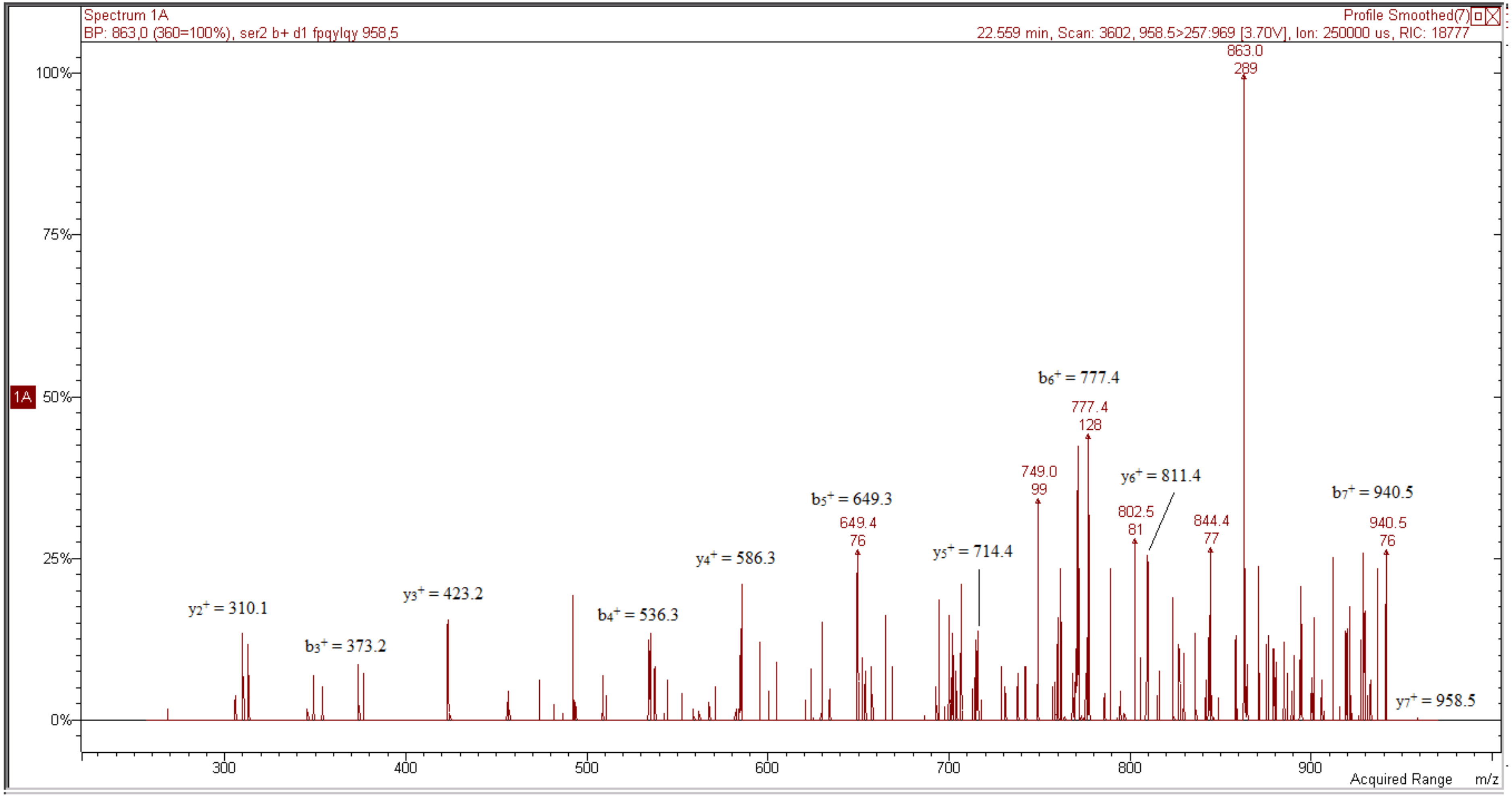

Figure S23. MS/MS spectrum of FPQYLQY peptide.

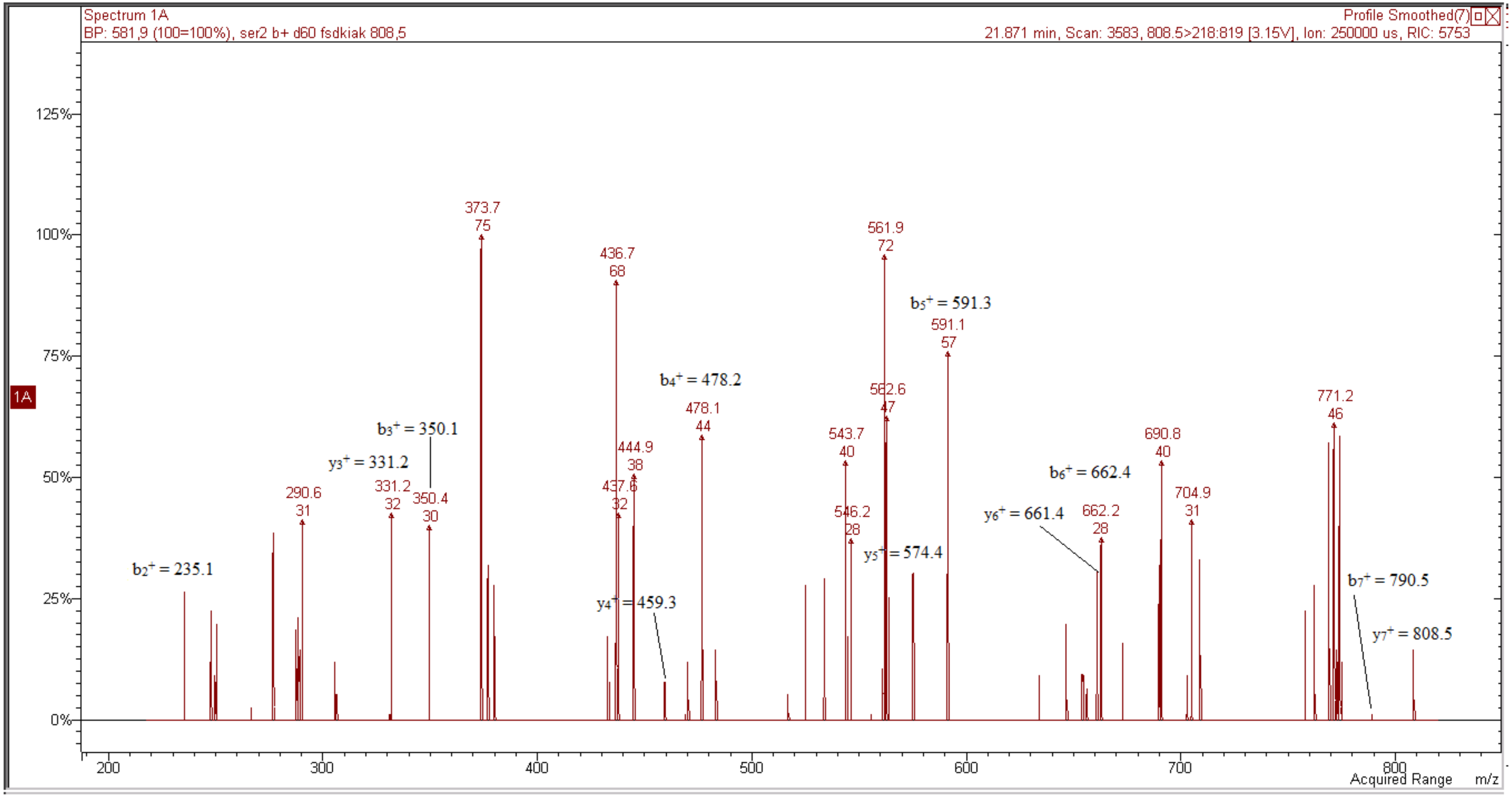

Figure S24. MS/MS spectrum of FSDKIAK peptide.

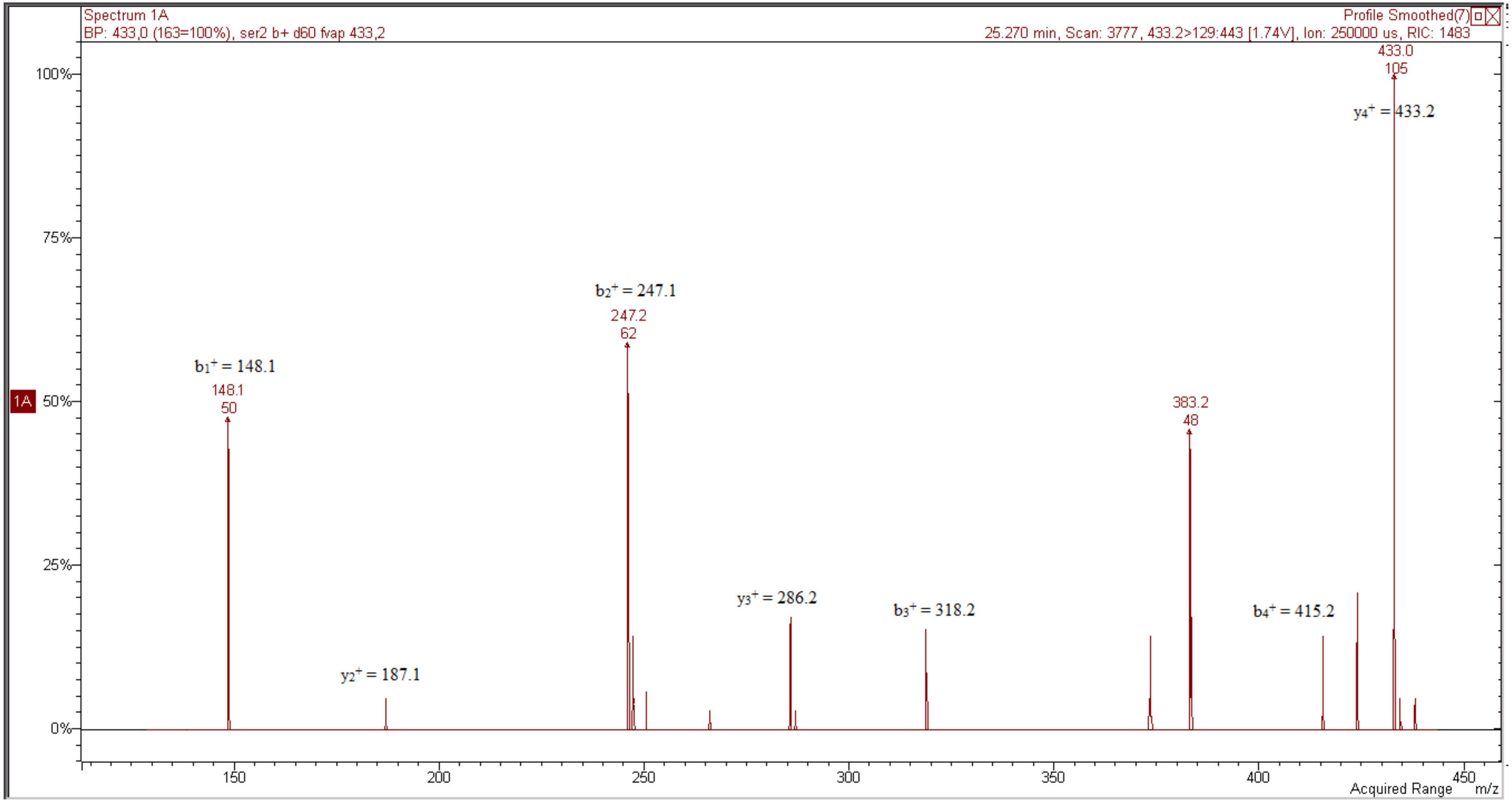

Figure S25. MS/MS spectrum of FVAP peptide.

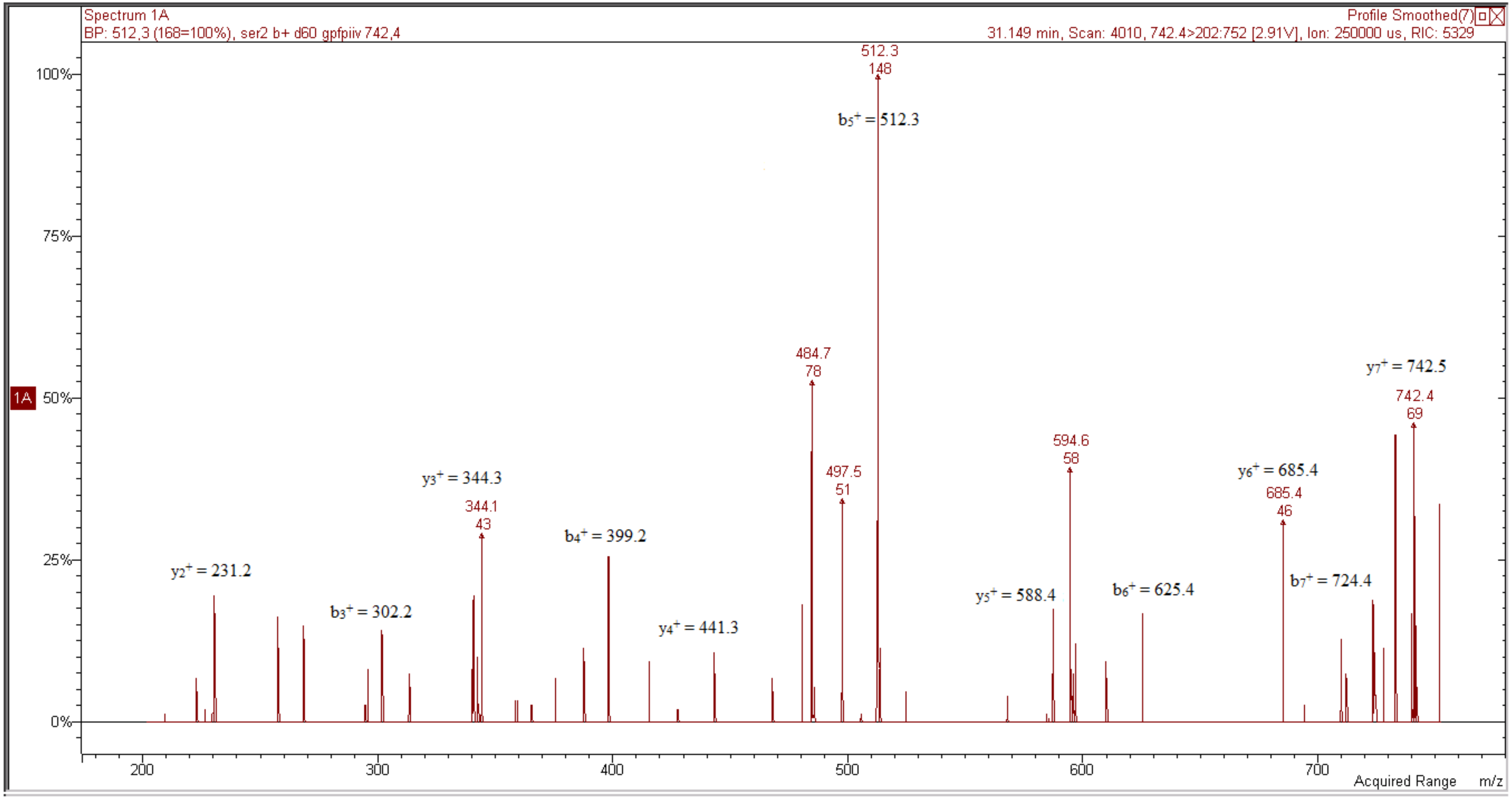

Figure S26. MS/MS spectrum of GPFPIIV peptide.

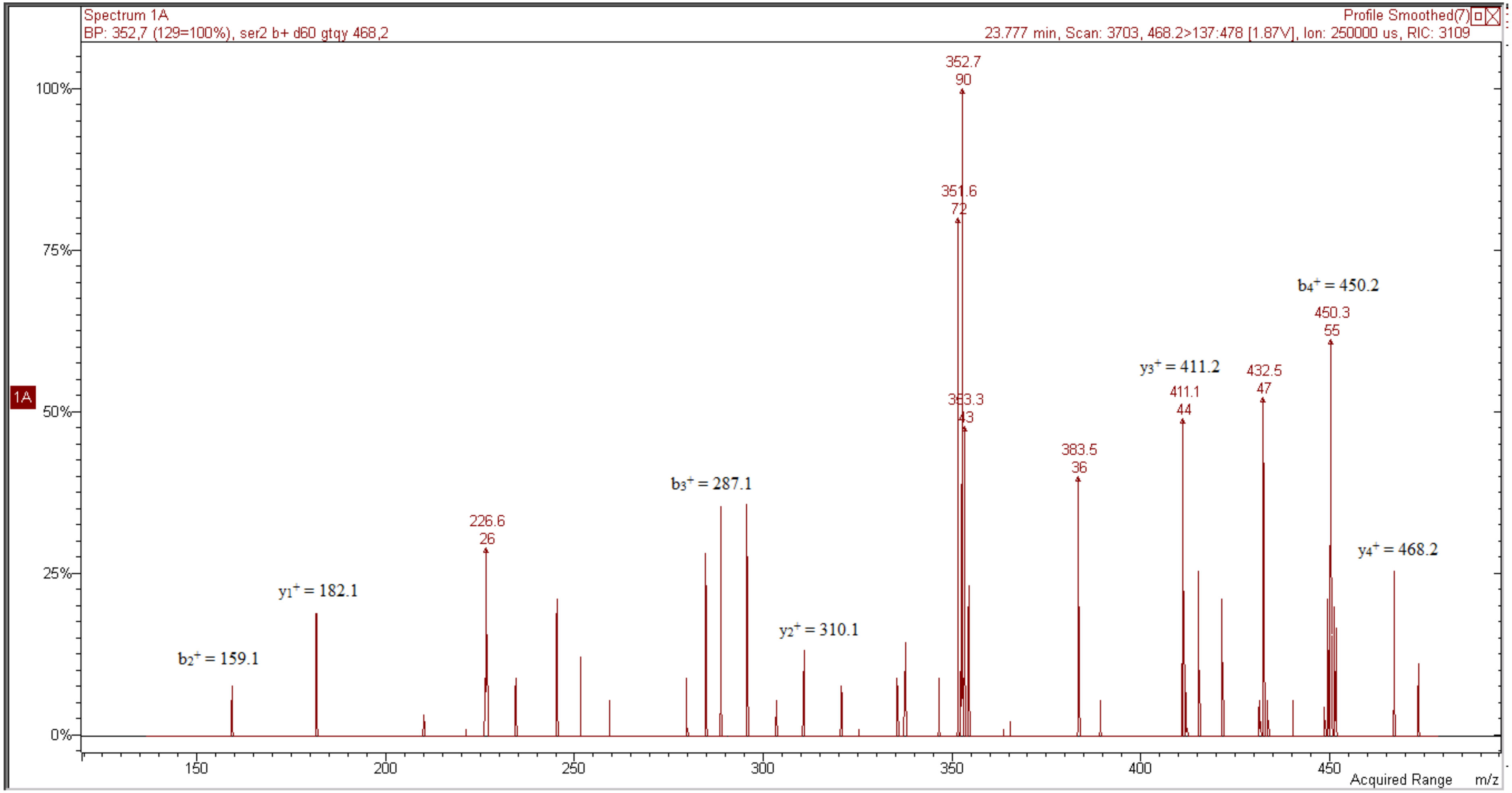

Figure S27. MS/MS spectrum of GTQY peptide.

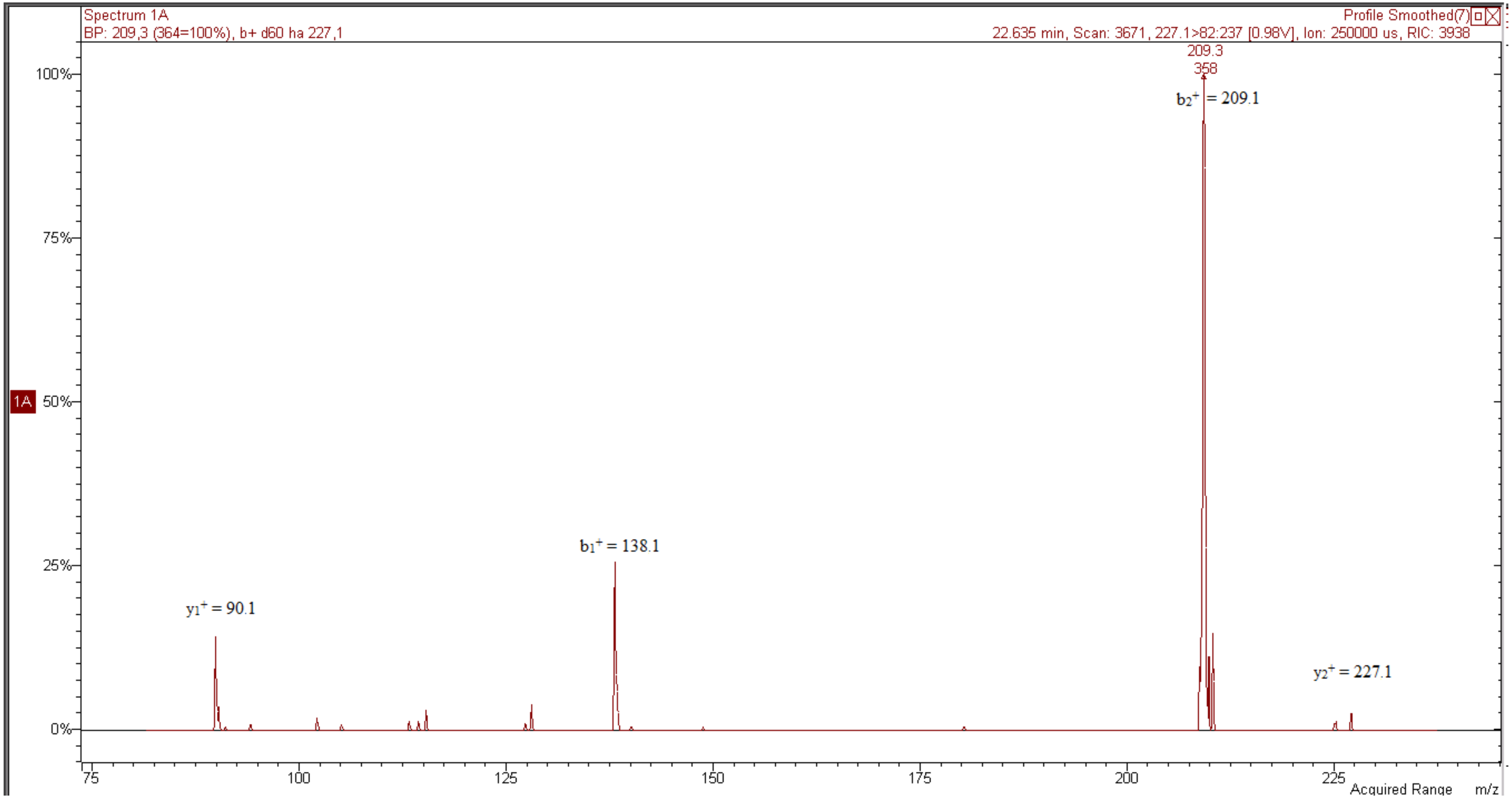

Figure S28. MS/MS spectrum of HA peptide.

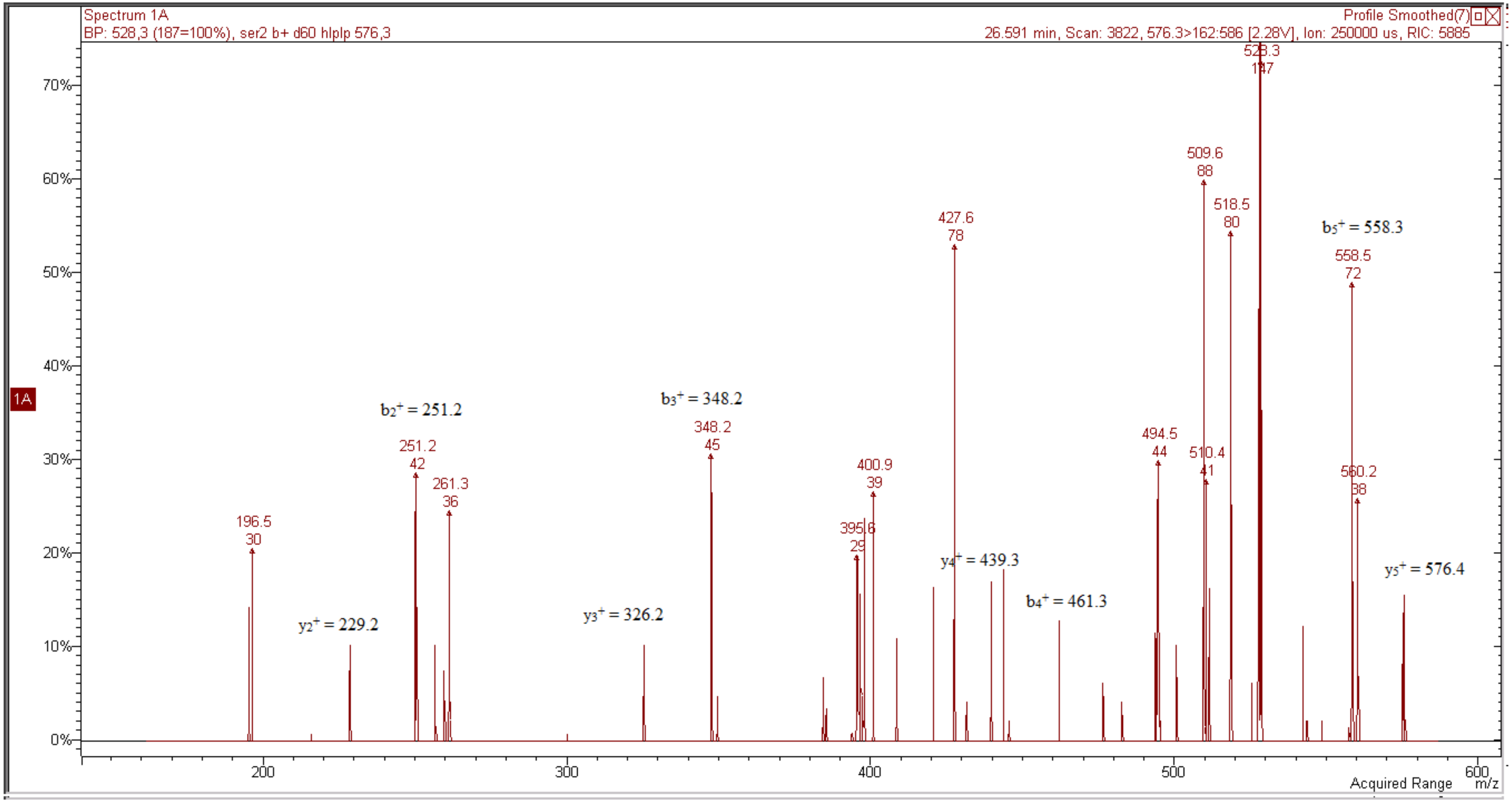

Figure S29. MS/MS spectrum of HLPLP peptide.

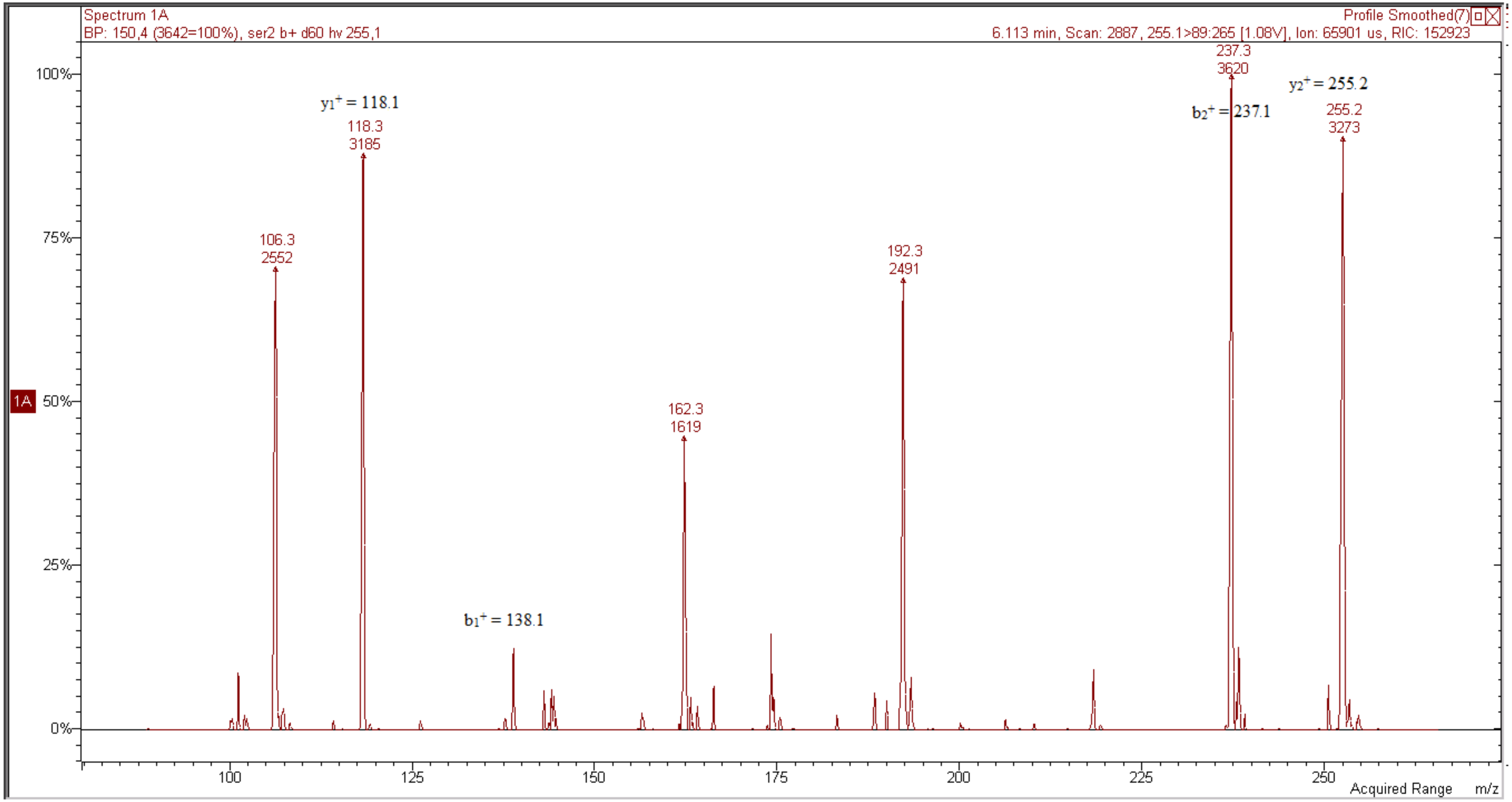

Figure S30. MS/MS spectrum of HV peptide.

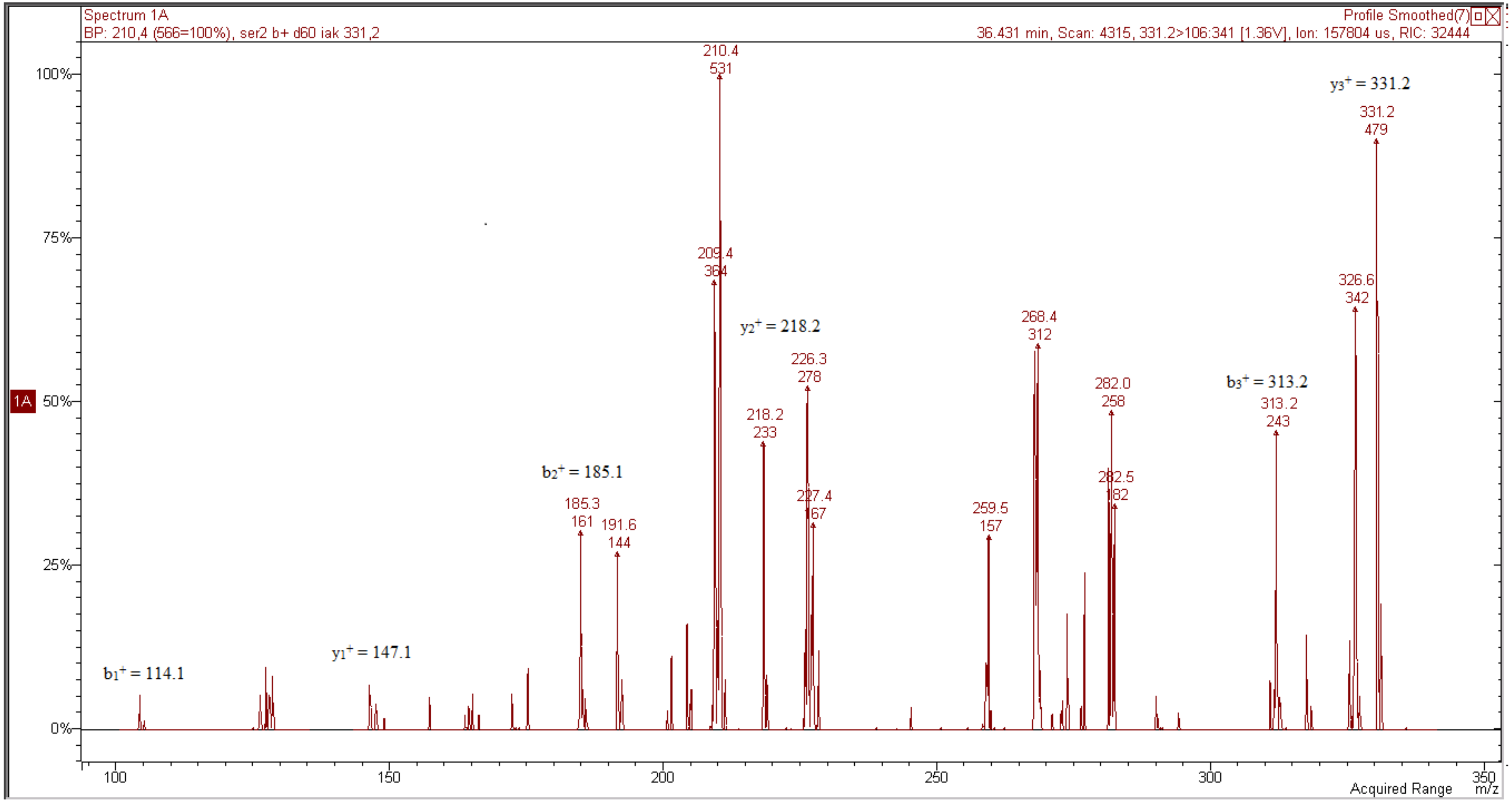

Figure S31. MS/MS spectrum of IAK peptide.

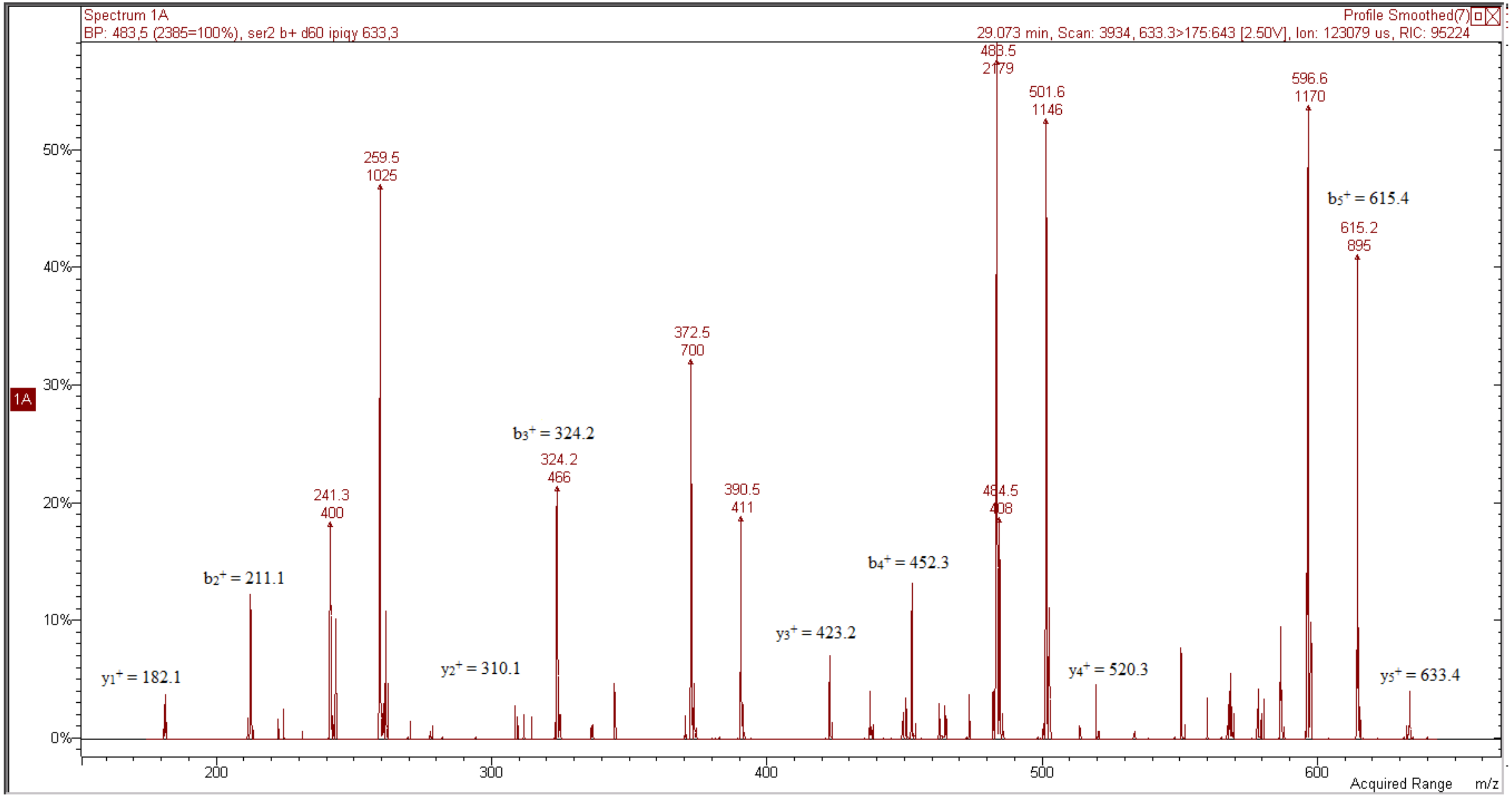

Figure S32. MS/MS spectrum of IPIQY peptide.

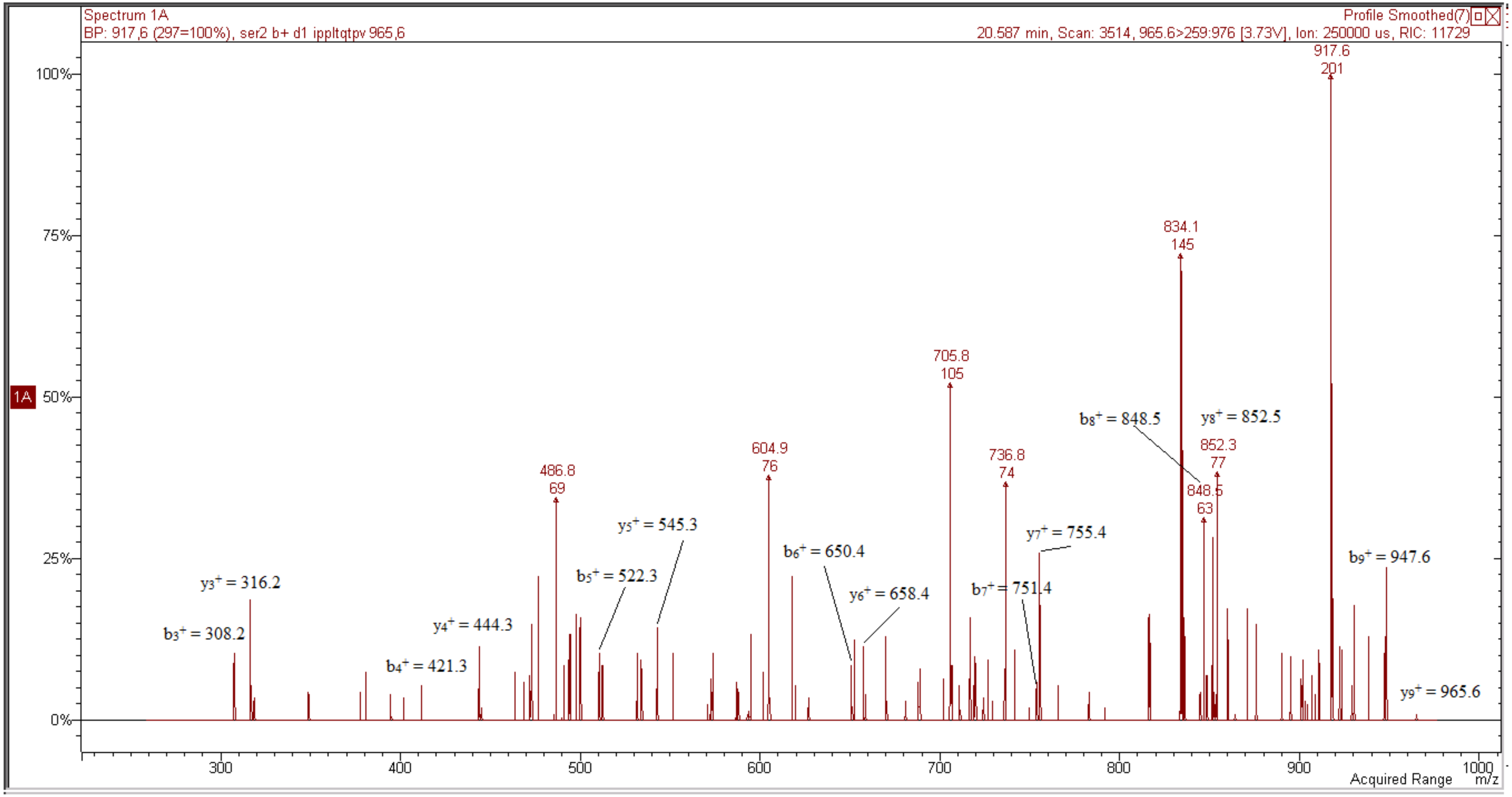

Figure S33. MS/MS spectrum of IPPLTQTPV peptide.

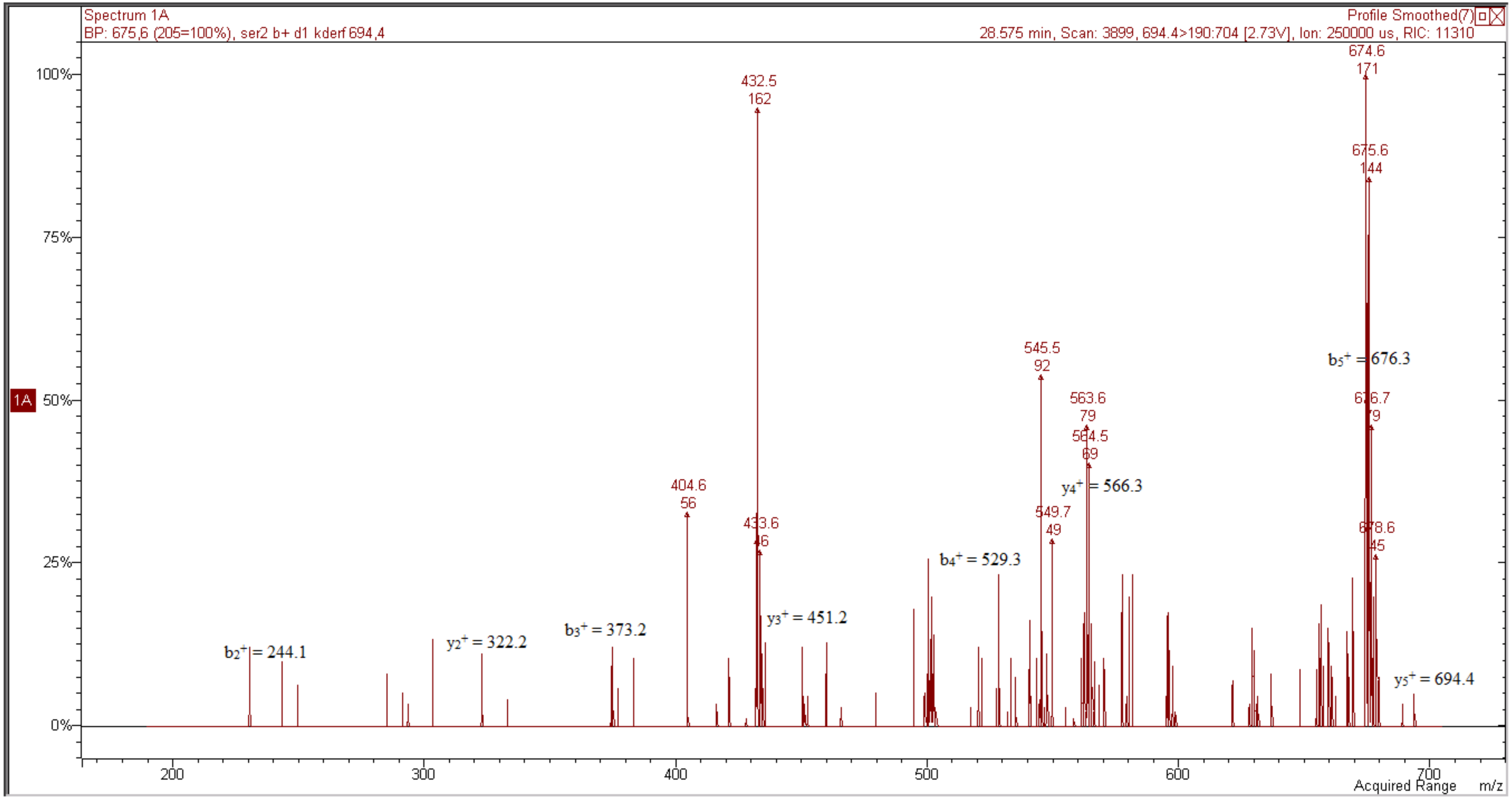

Figure S34. MS/MS spectrum of KDERF peptide.

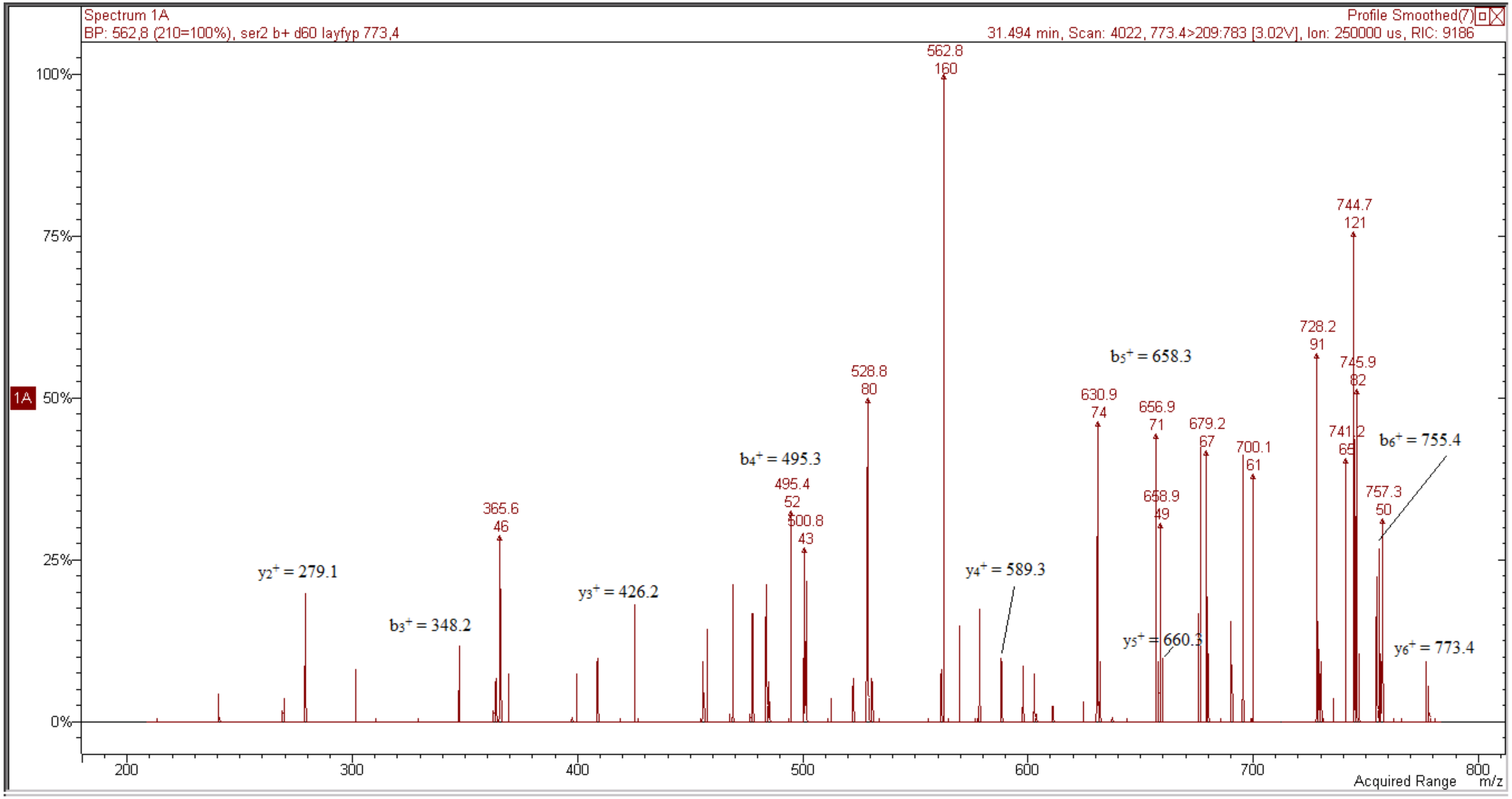

Figure S35. MS/MS spectrum of LAYFYP peptide.

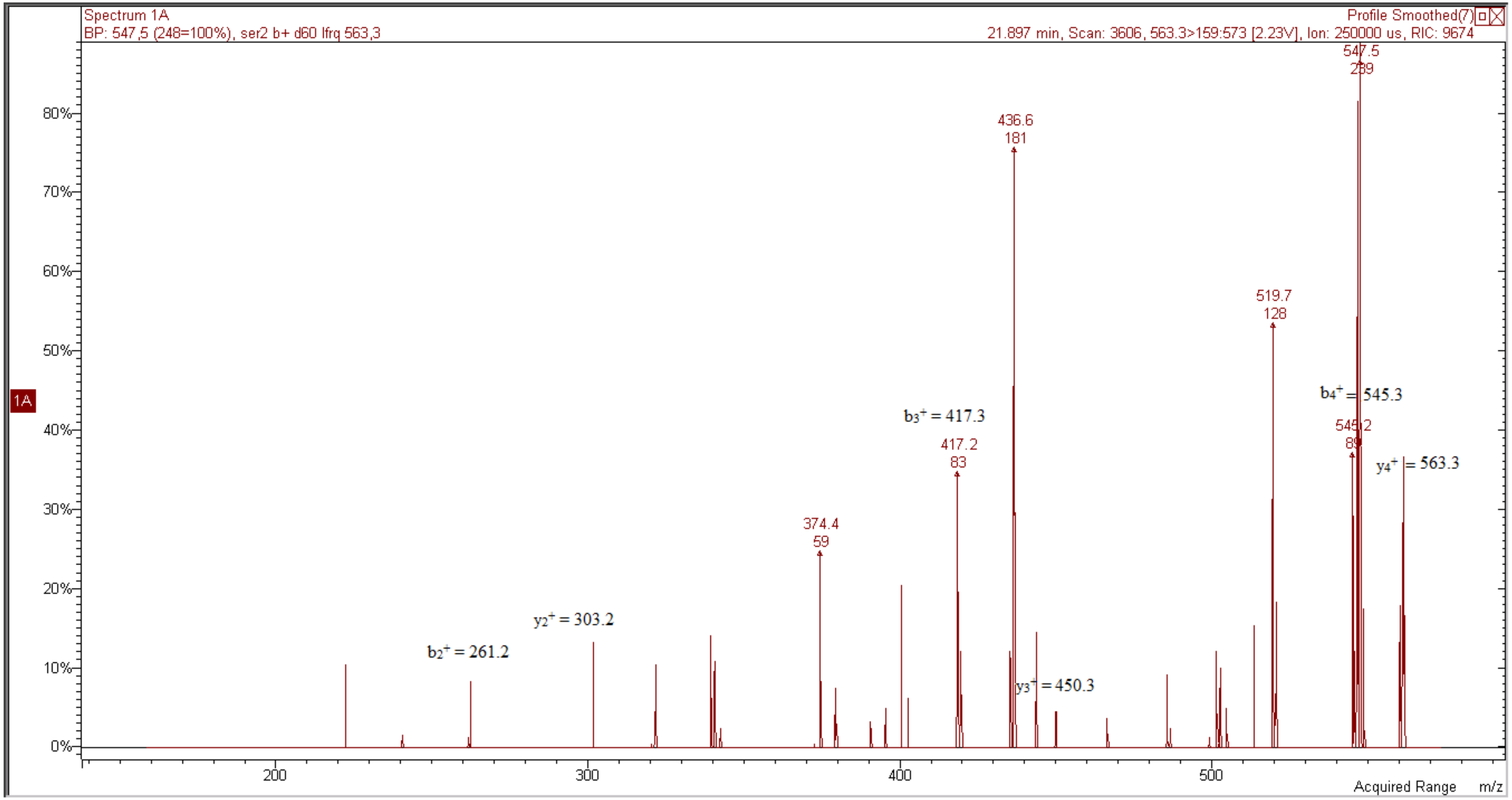

Figure S36. MS/MS spectrum of LFRQ peptide.

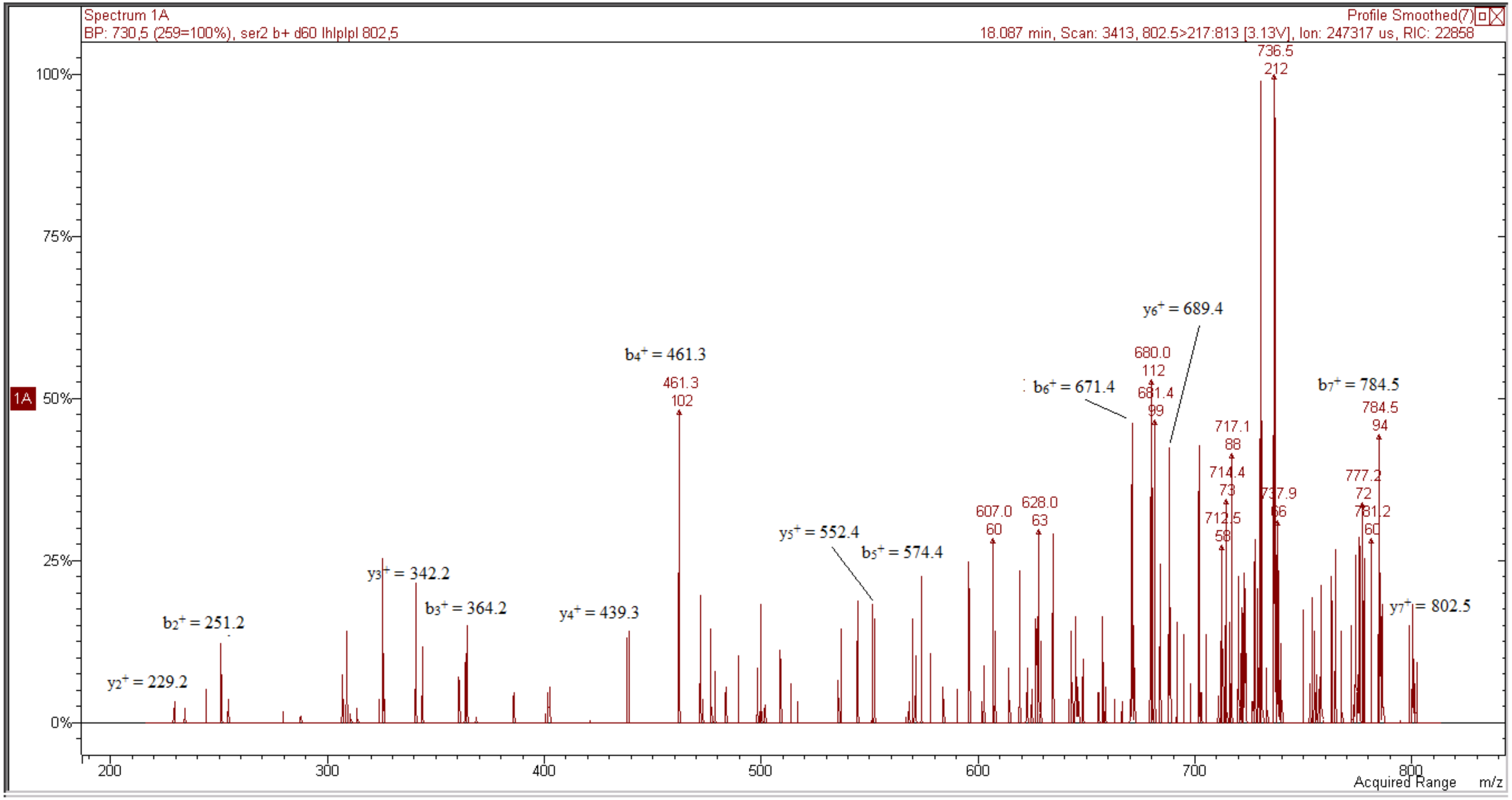

Figure S37. MS/MS spectrum of LHLPLP peptide.

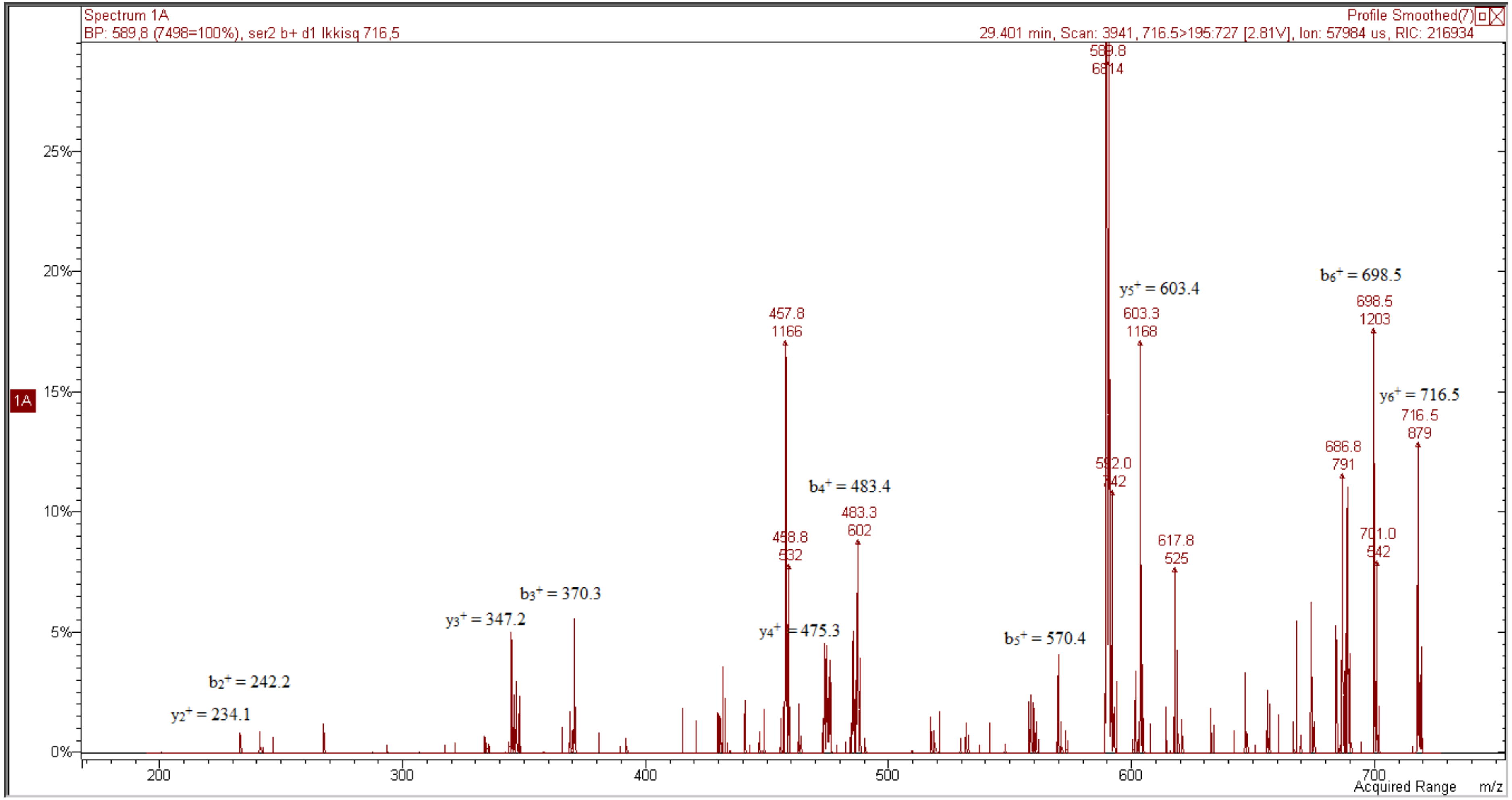

Figure S35. MS/MS spectrum of LKKISQ peptide.

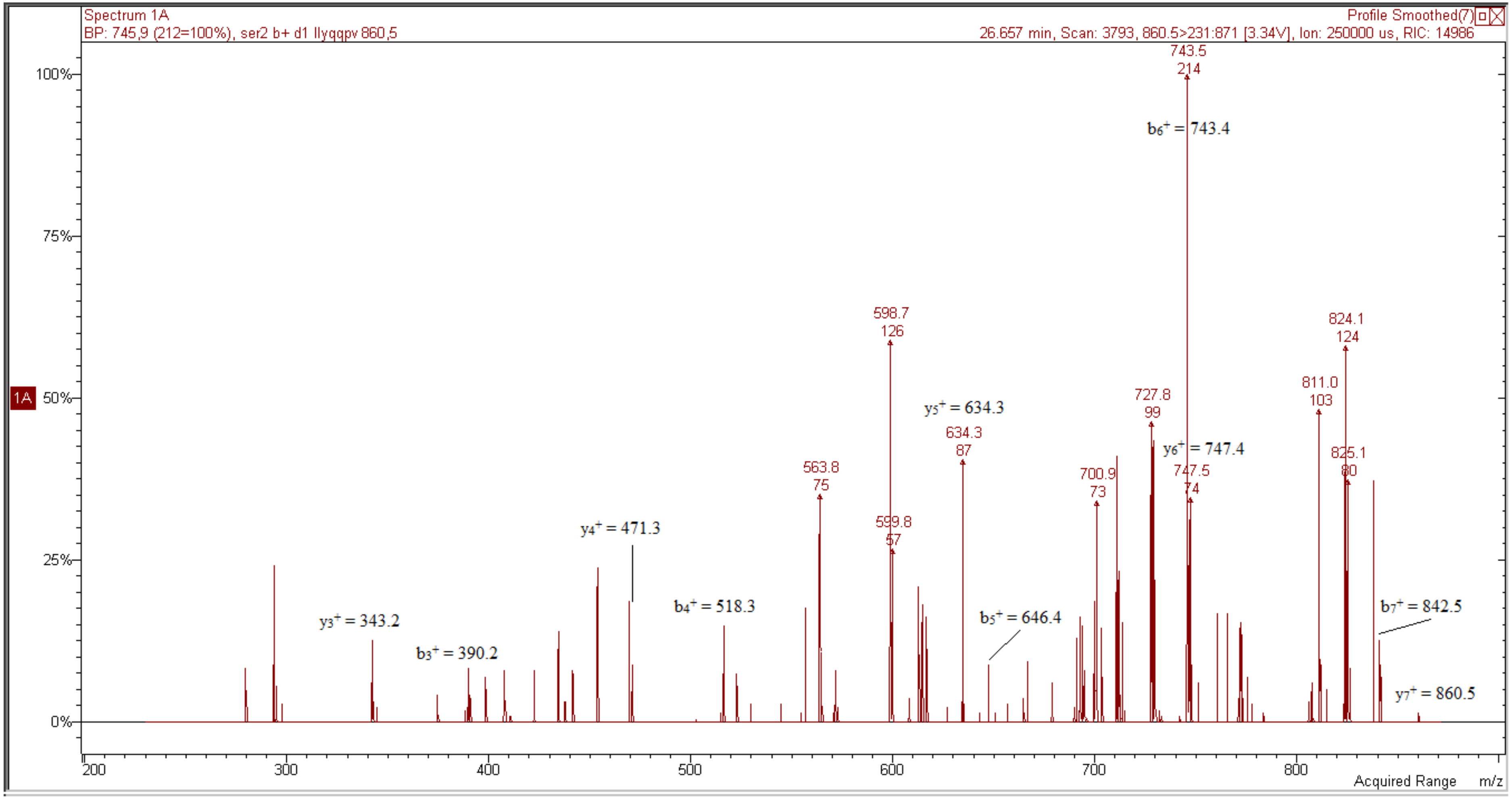

Figure S39. MS/MS spectrum of LLYQQPV peptide.

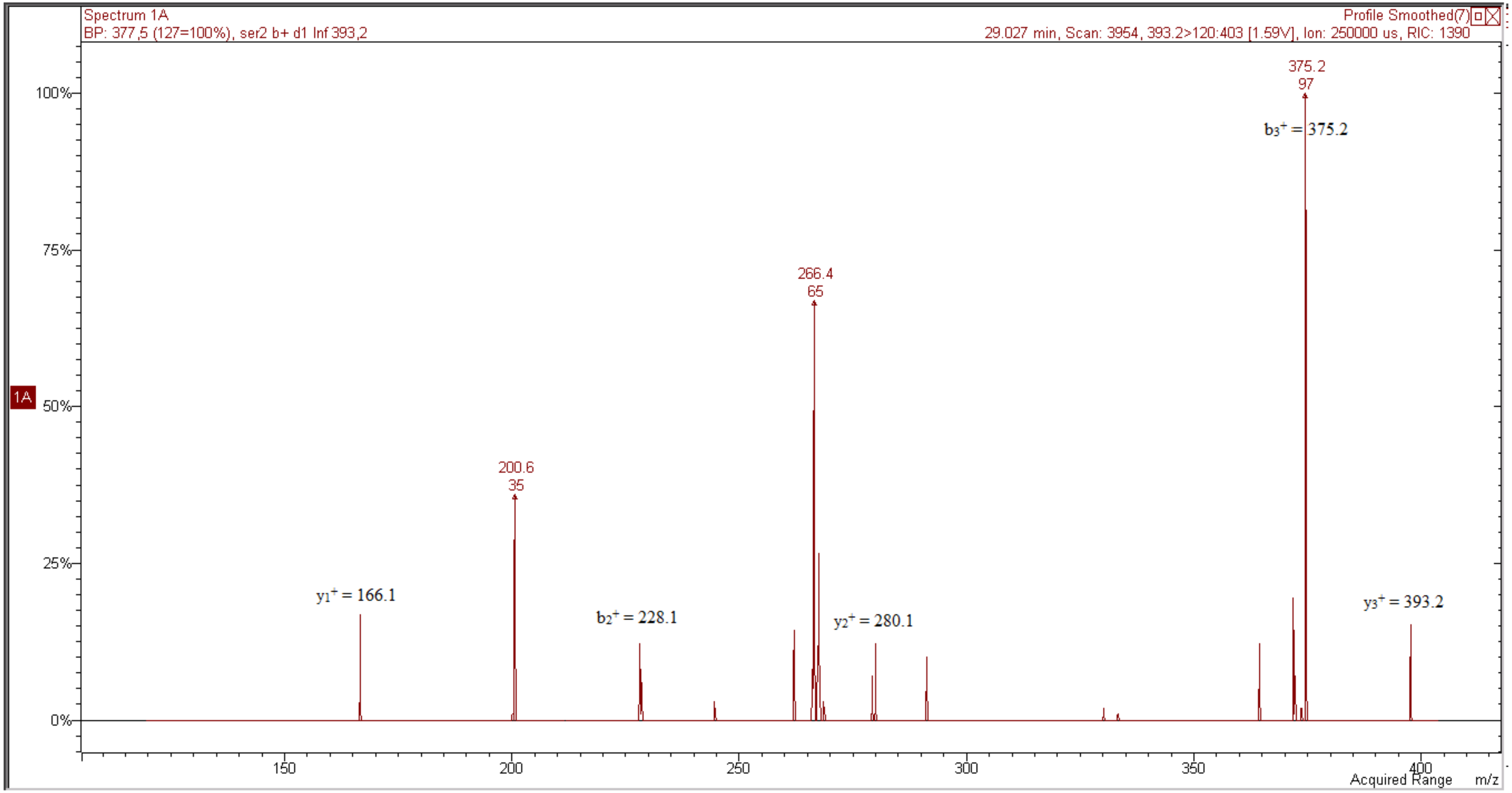

Figure S40. MS/MS spectrum of LNF peptide.

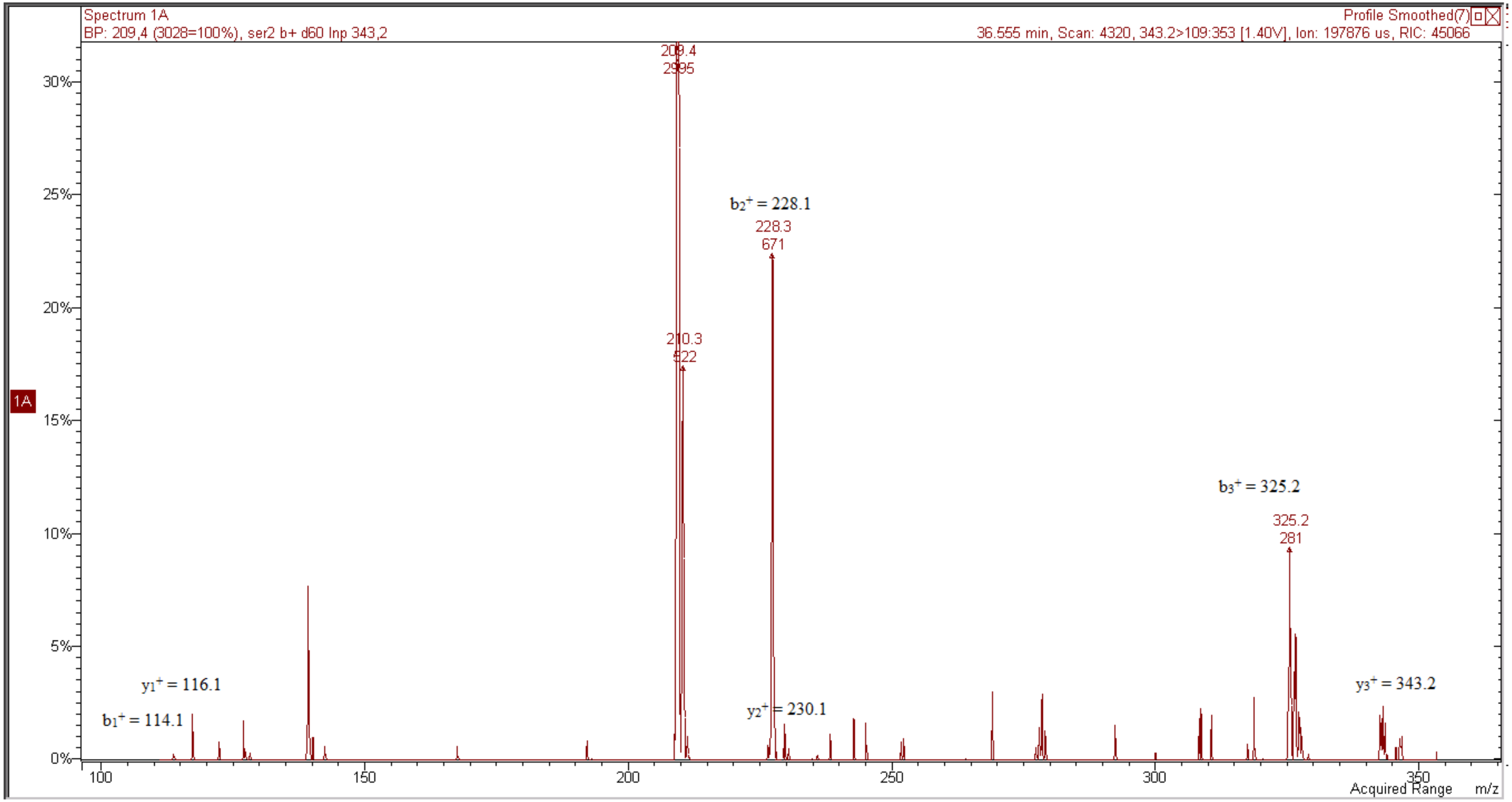

Figure S41. MS/MS spectrum of LNP peptide.

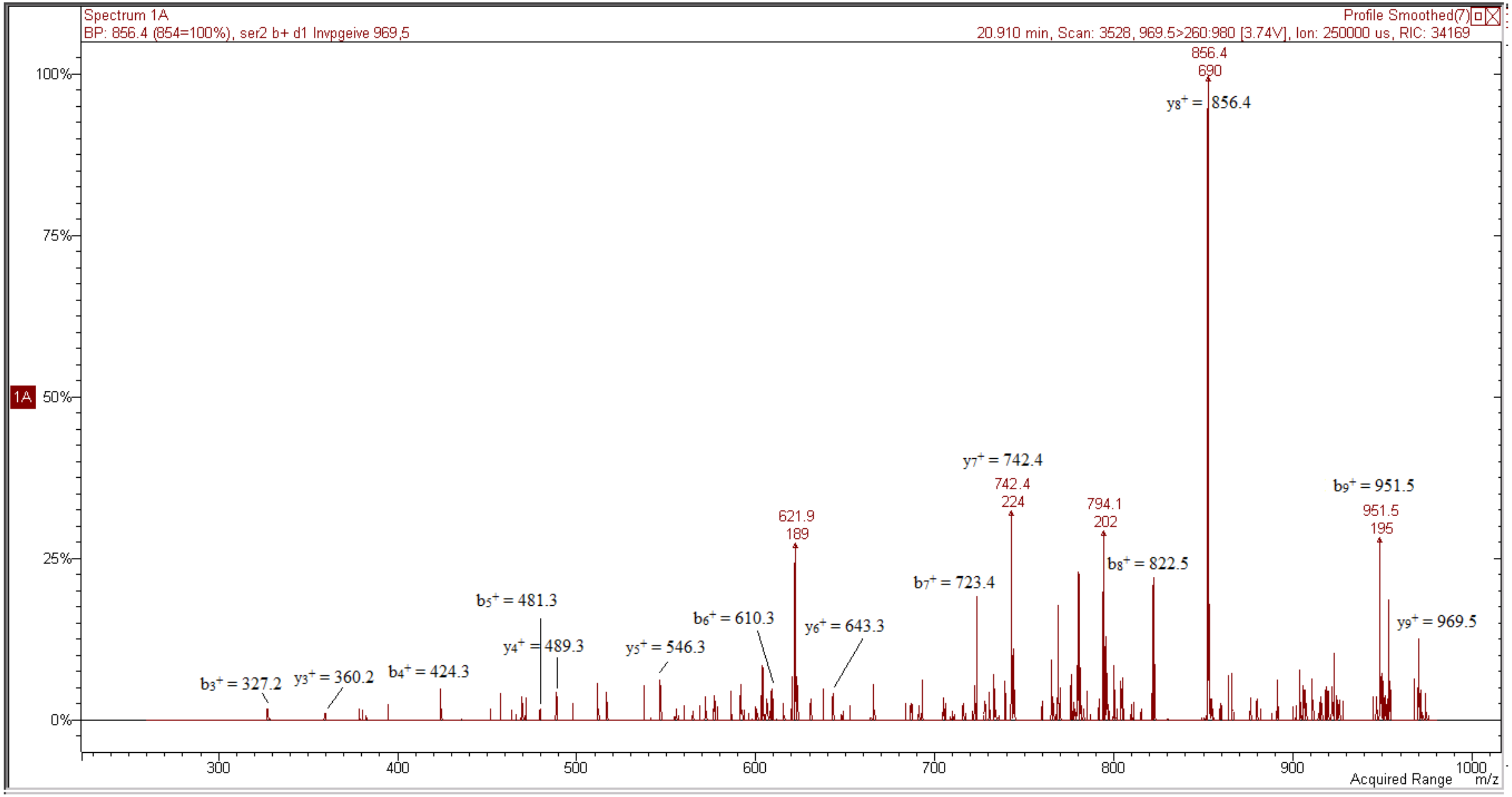

Figure S42. MS/MS spectrum of LNVPGEIVE peptide.

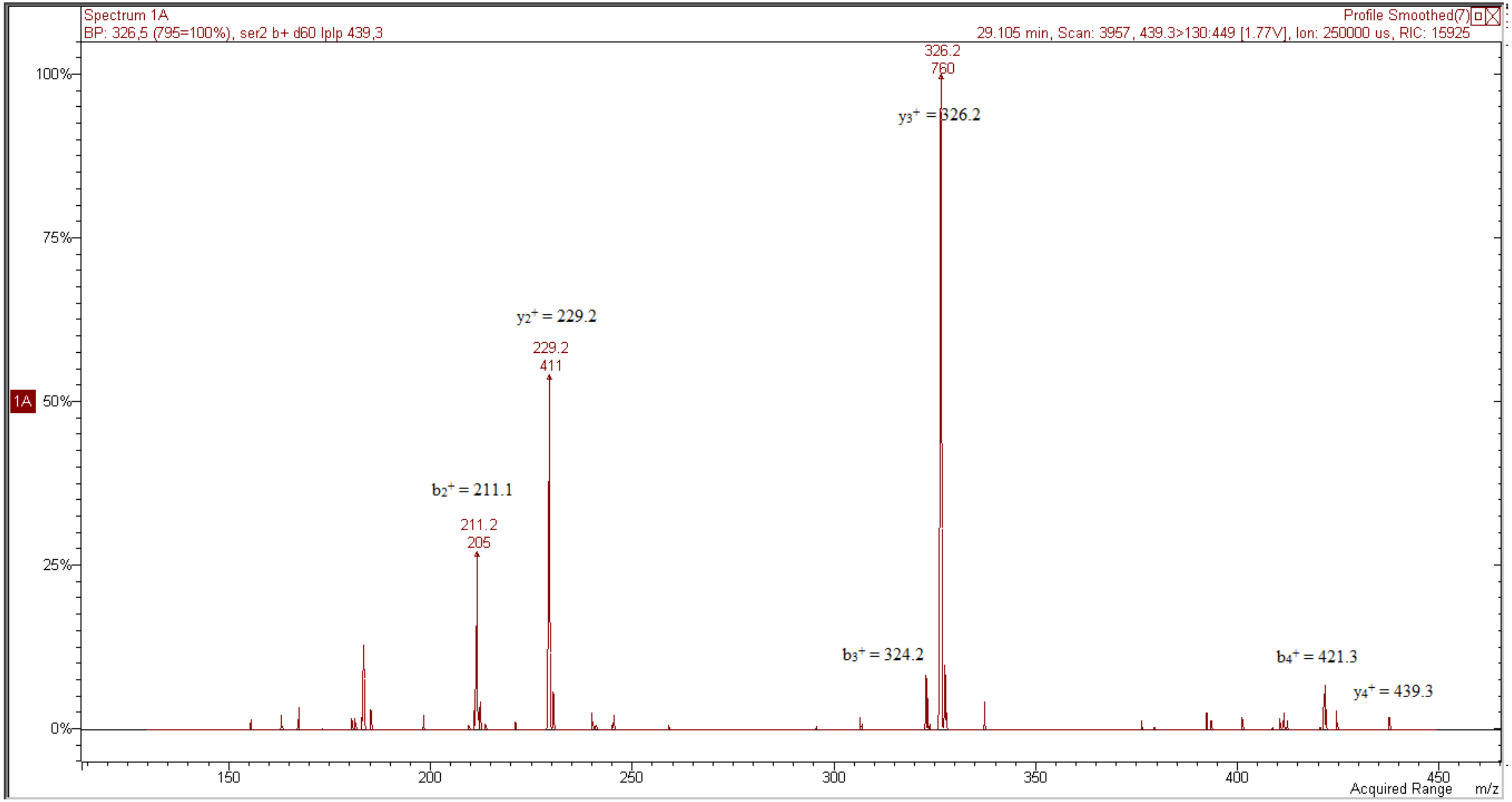

Figure S43. MS/MS spectrum of LPLP peptide.

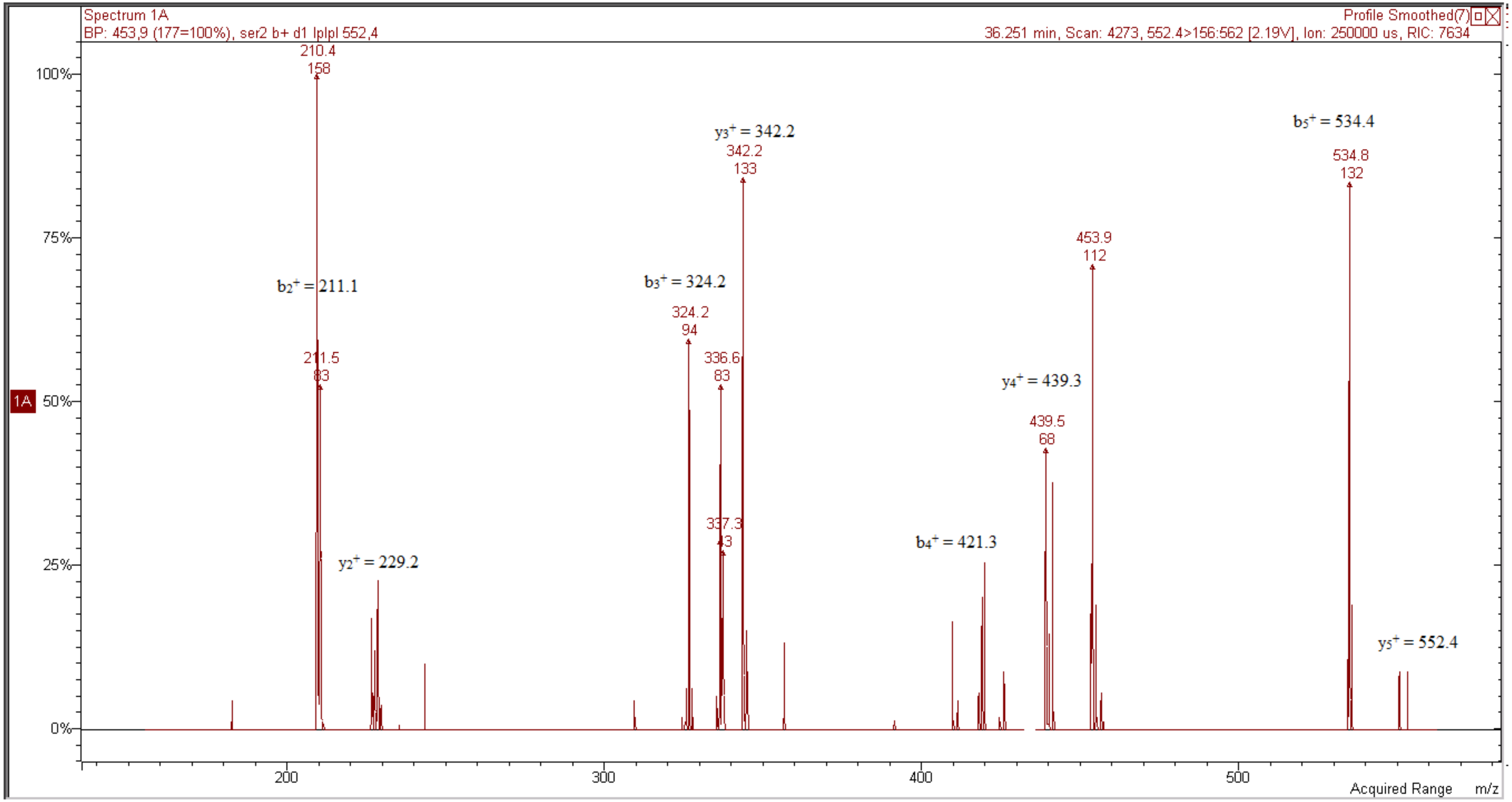

Figure S44. MS/MS spectrum of LPLPL peptide.

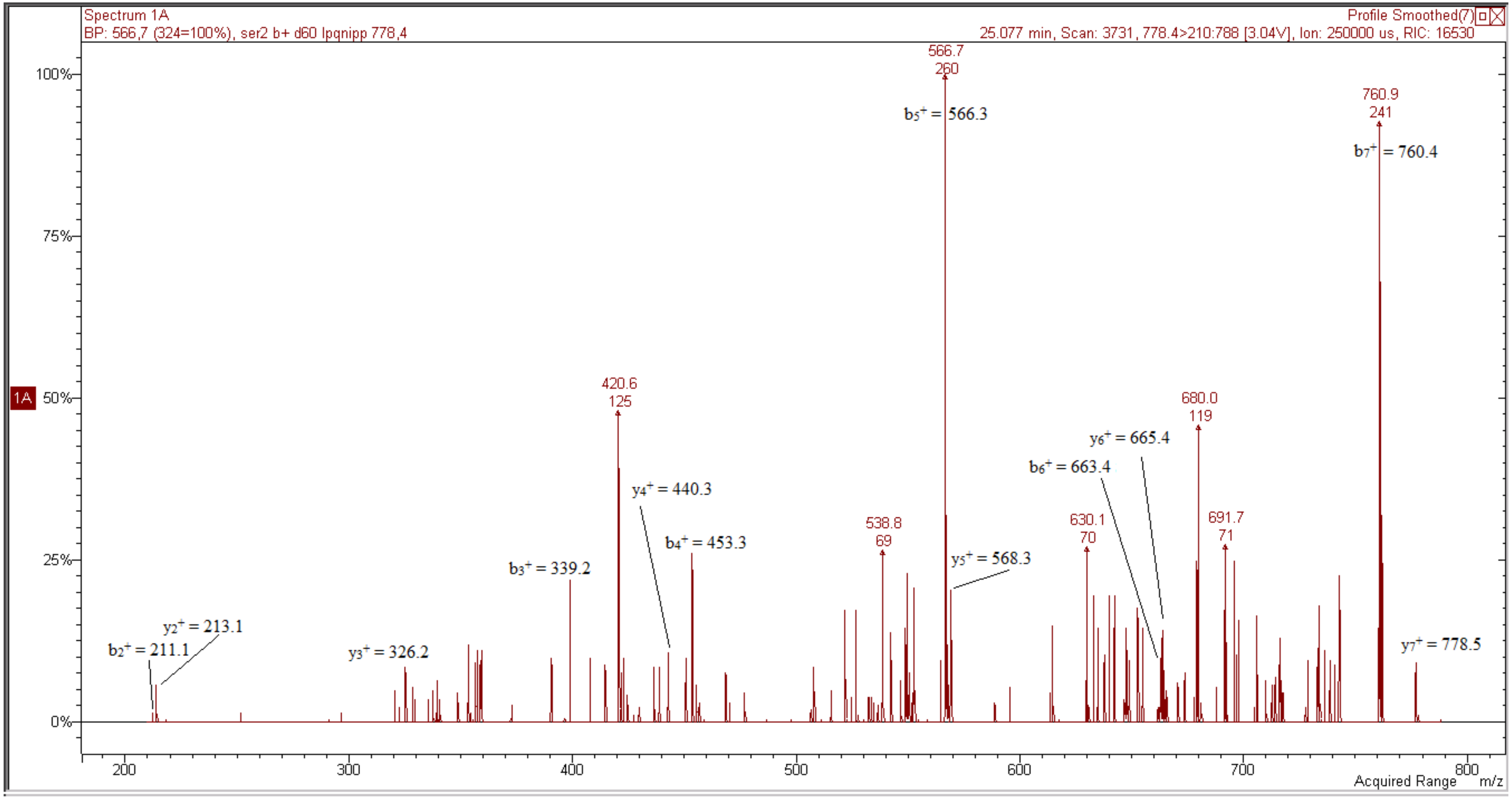

Figure S45. MS/MS spectrum of LPQNIPP peptide.

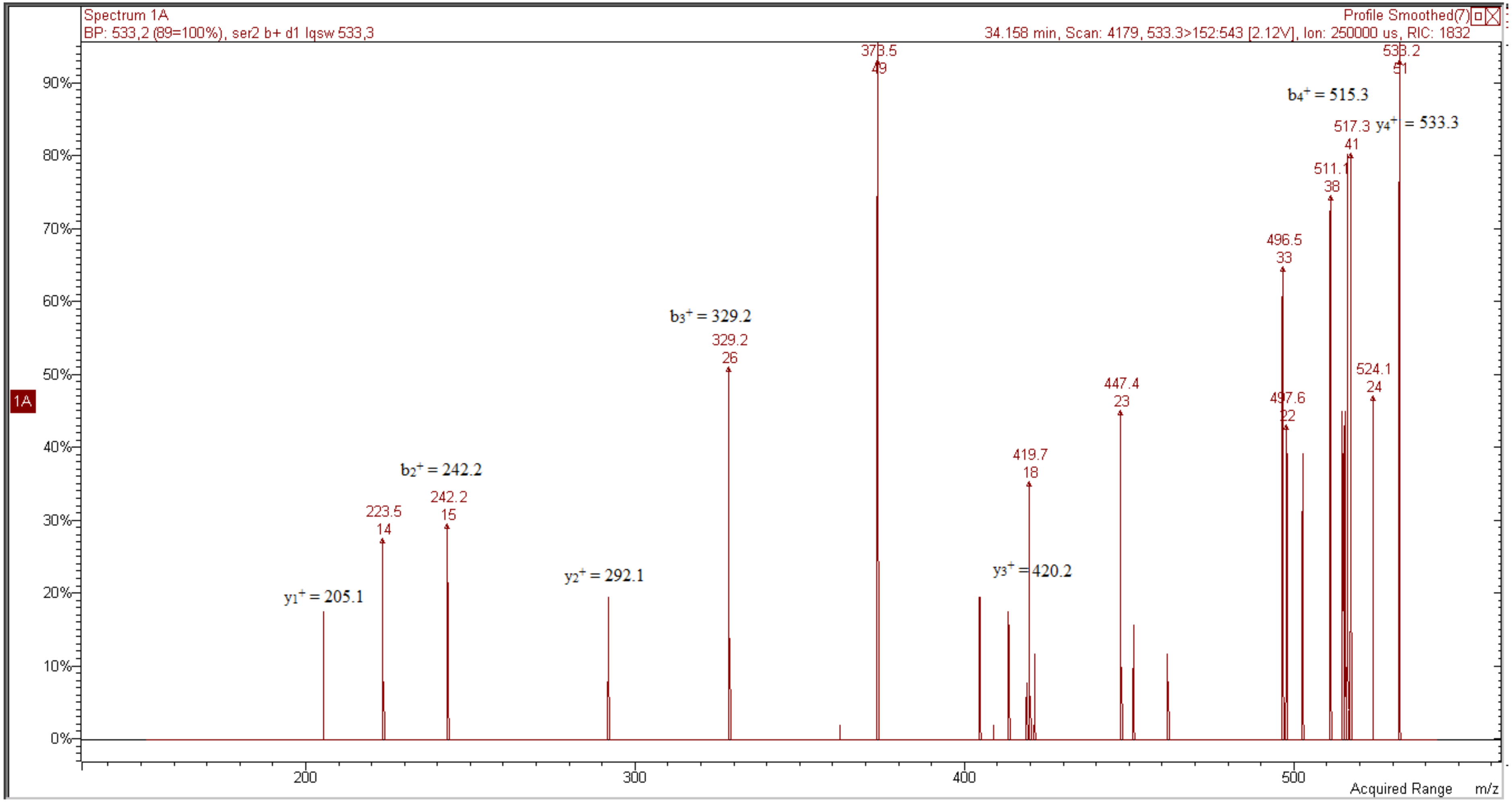

Figure S46. MS/MS spectrum of LQSW peptide.

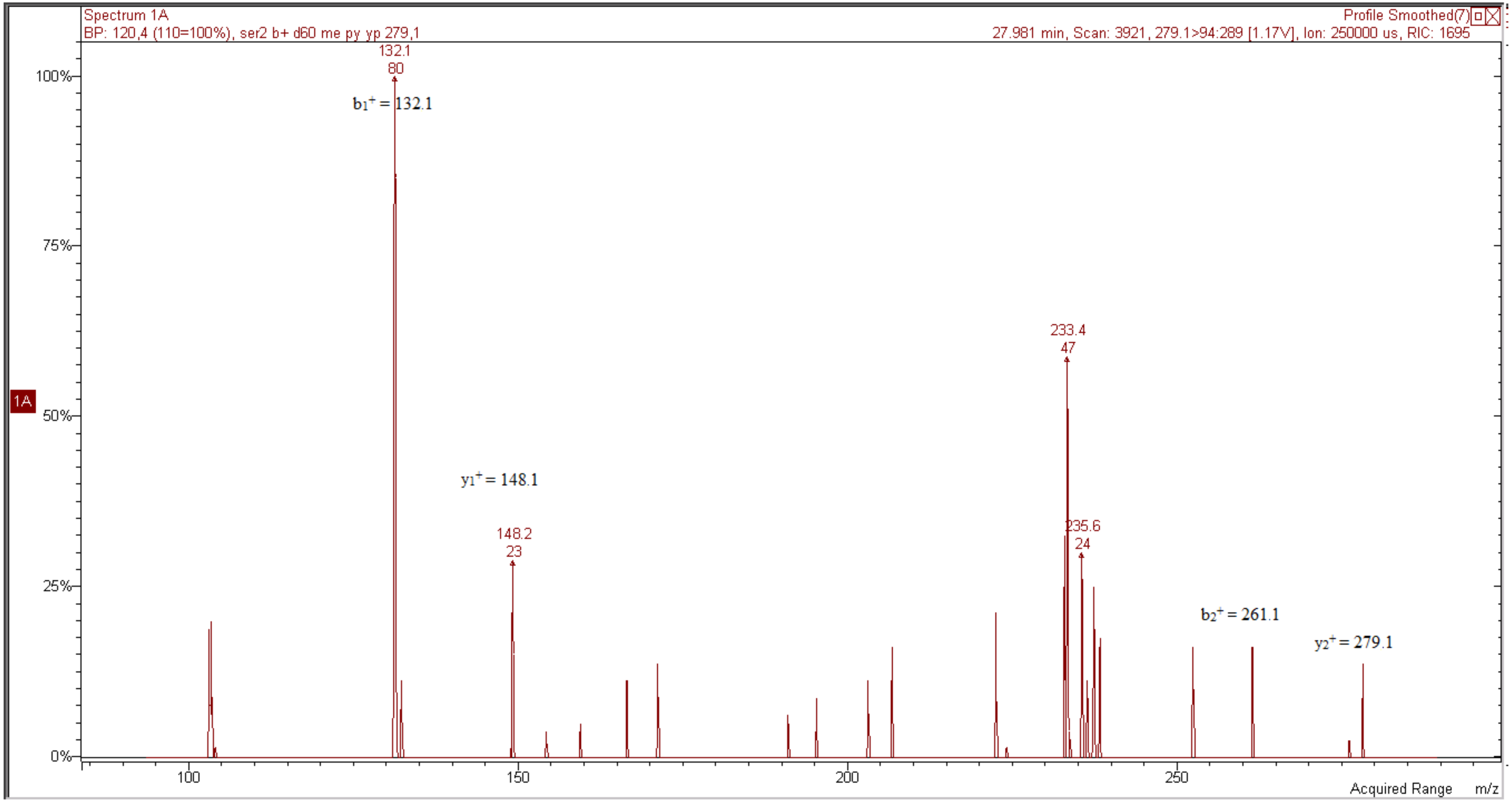

Figure S47. MS/MS spectrum of ME peptide.

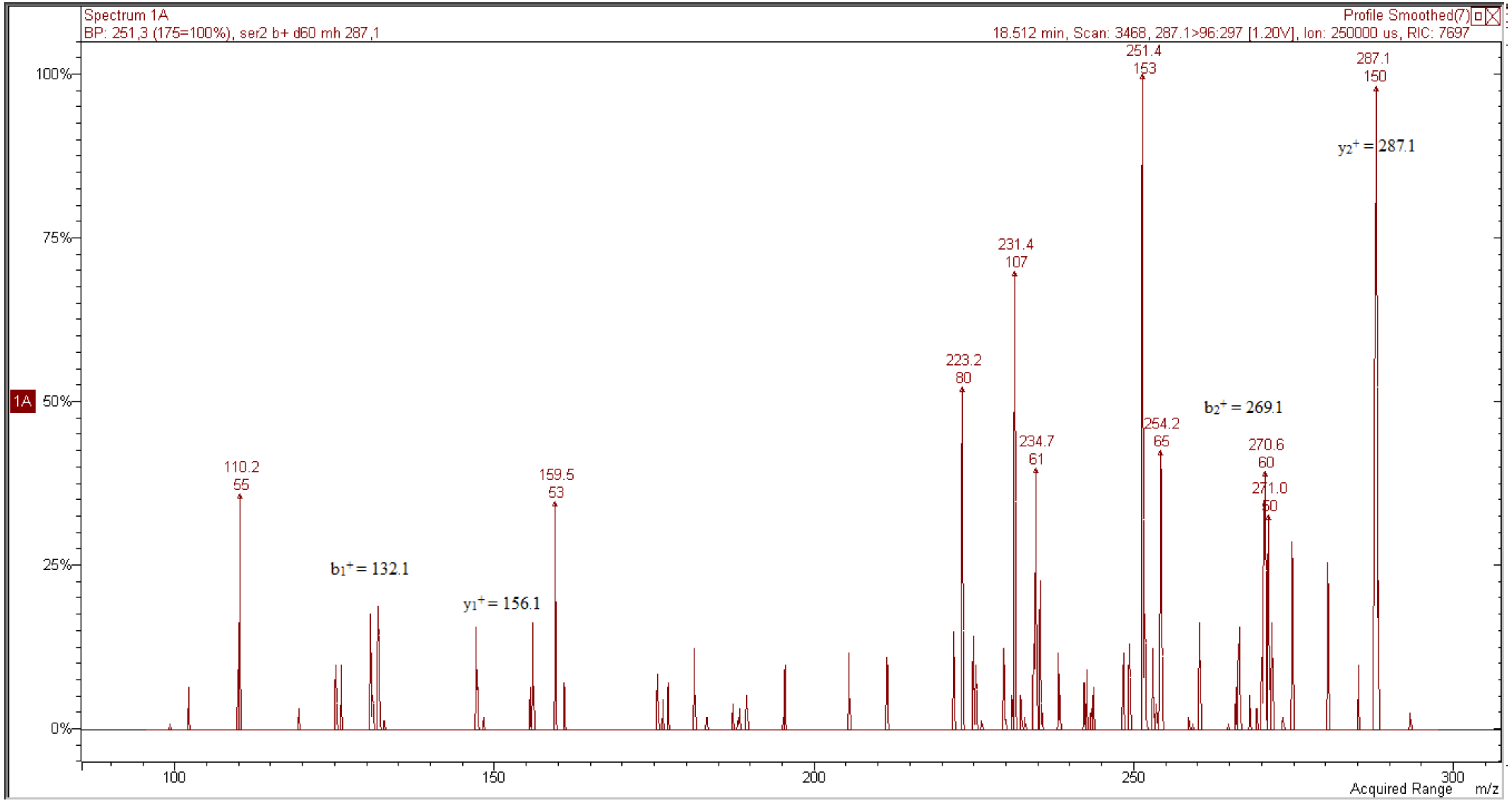

Figure S48. MS/MS spectrum of MH peptide.

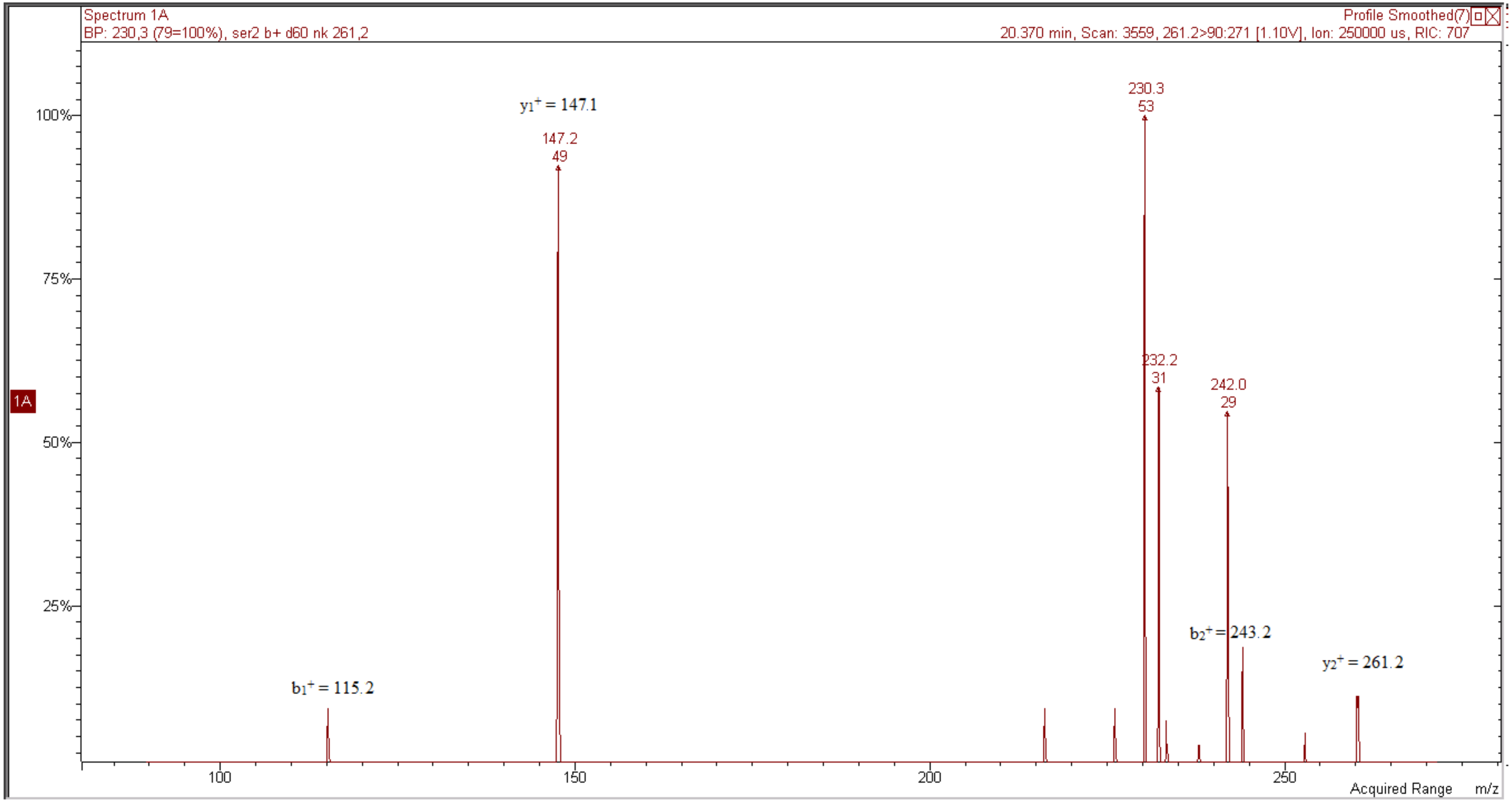

Figure S48. MS/MS spectrum of NK peptide.

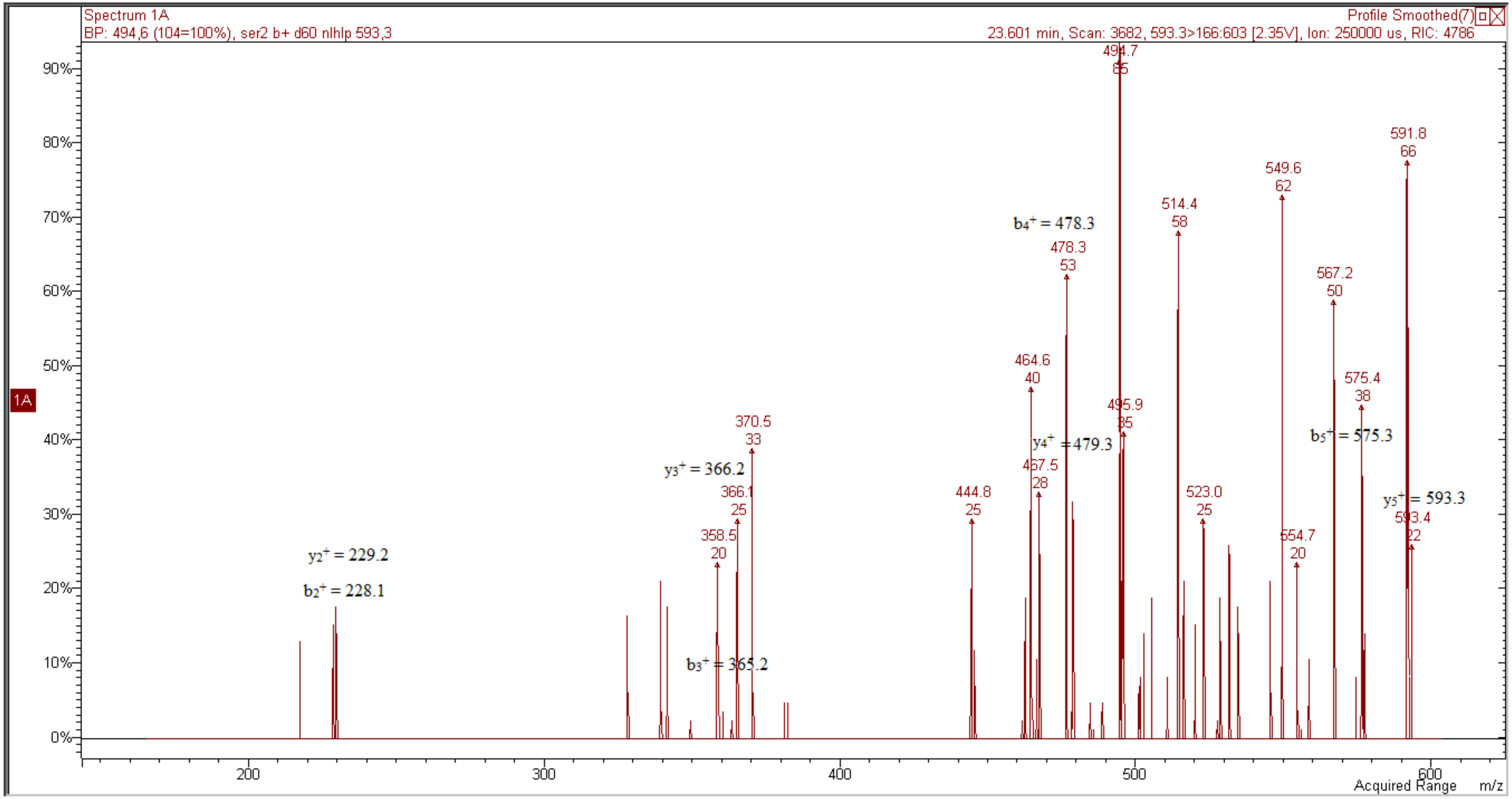

Figure S50. MS/MS spectrum of NLHLP peptide.

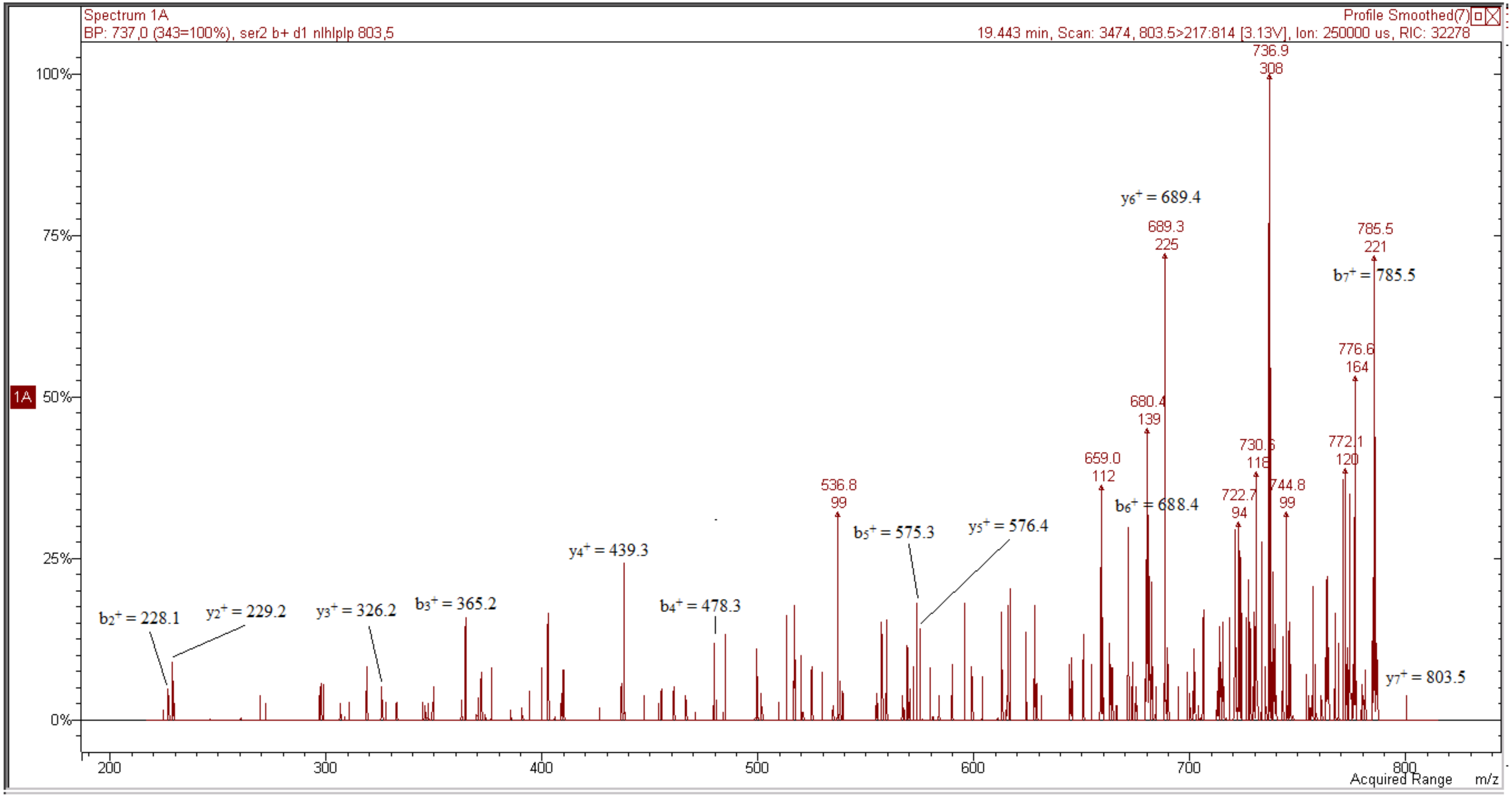

Figure S51. MS/MS spectrum of NLHLPLP peptide.

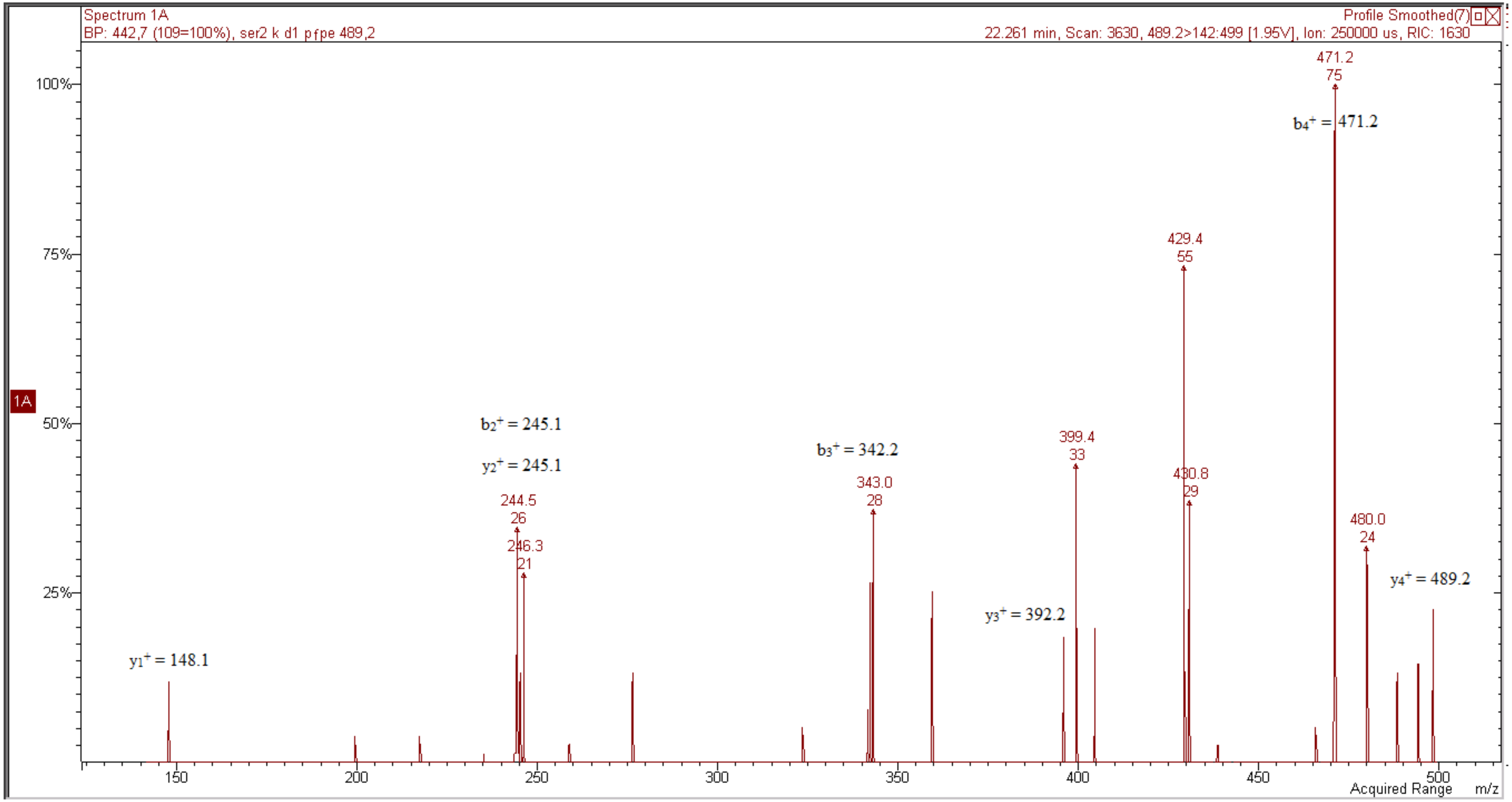

Figure S52. MS/MS spectrum of PFPE peptide.

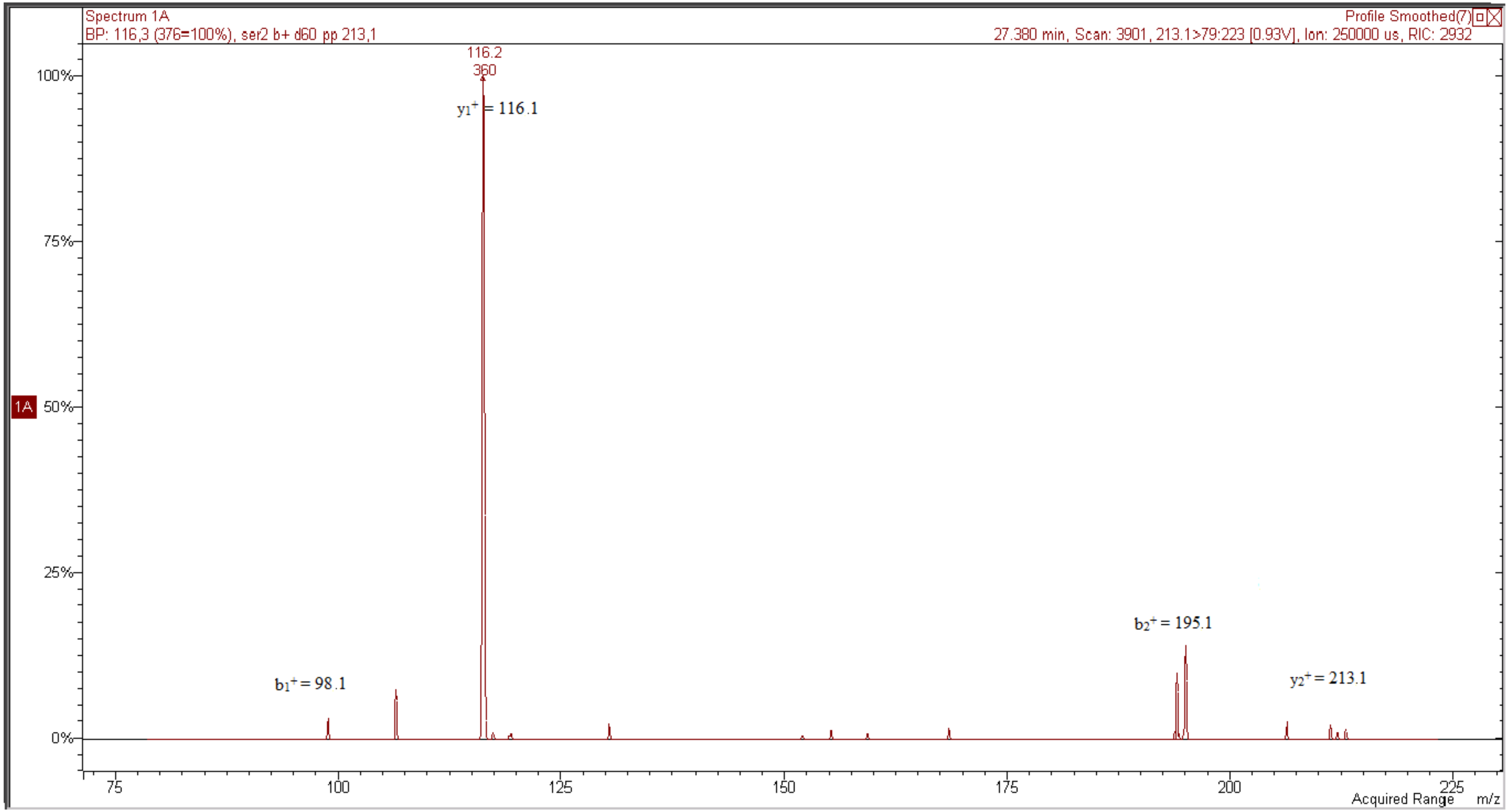

Figure S53. MS/MS spectrum of PP peptide.

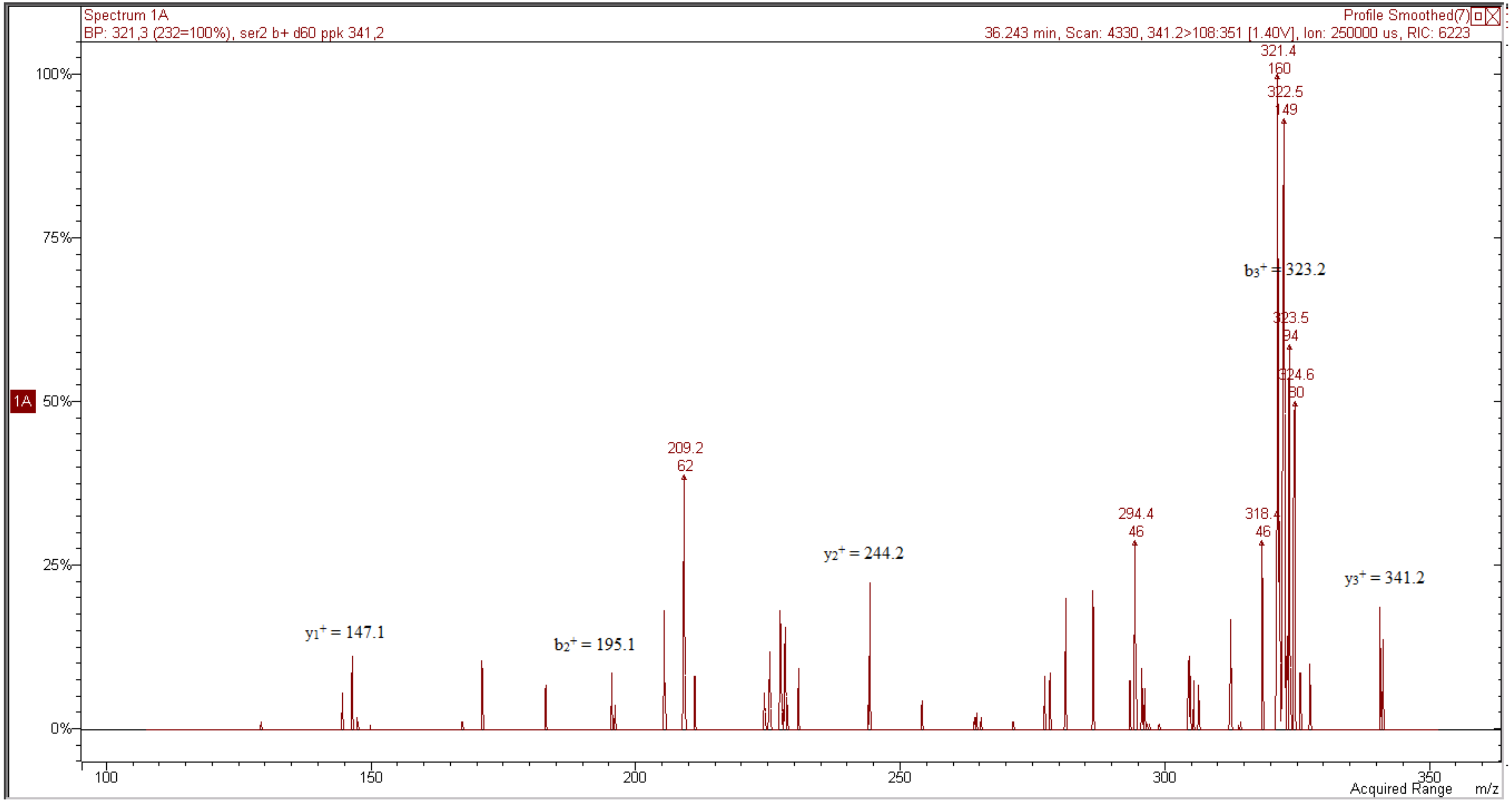

Figure S54. MS/MS spectrum of PPK peptide.

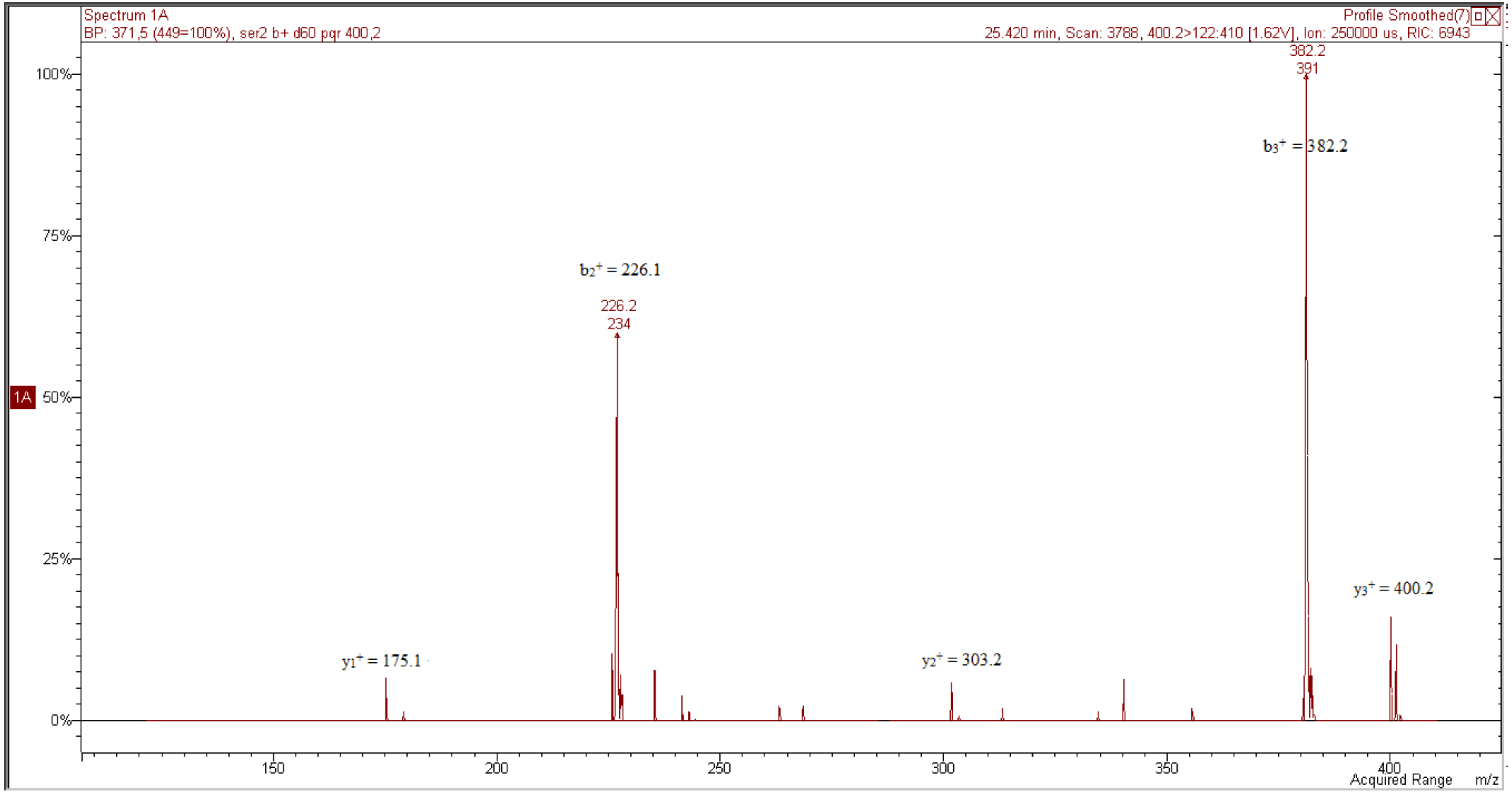

Figure S55. MS/MS spectrum of PQR peptide.

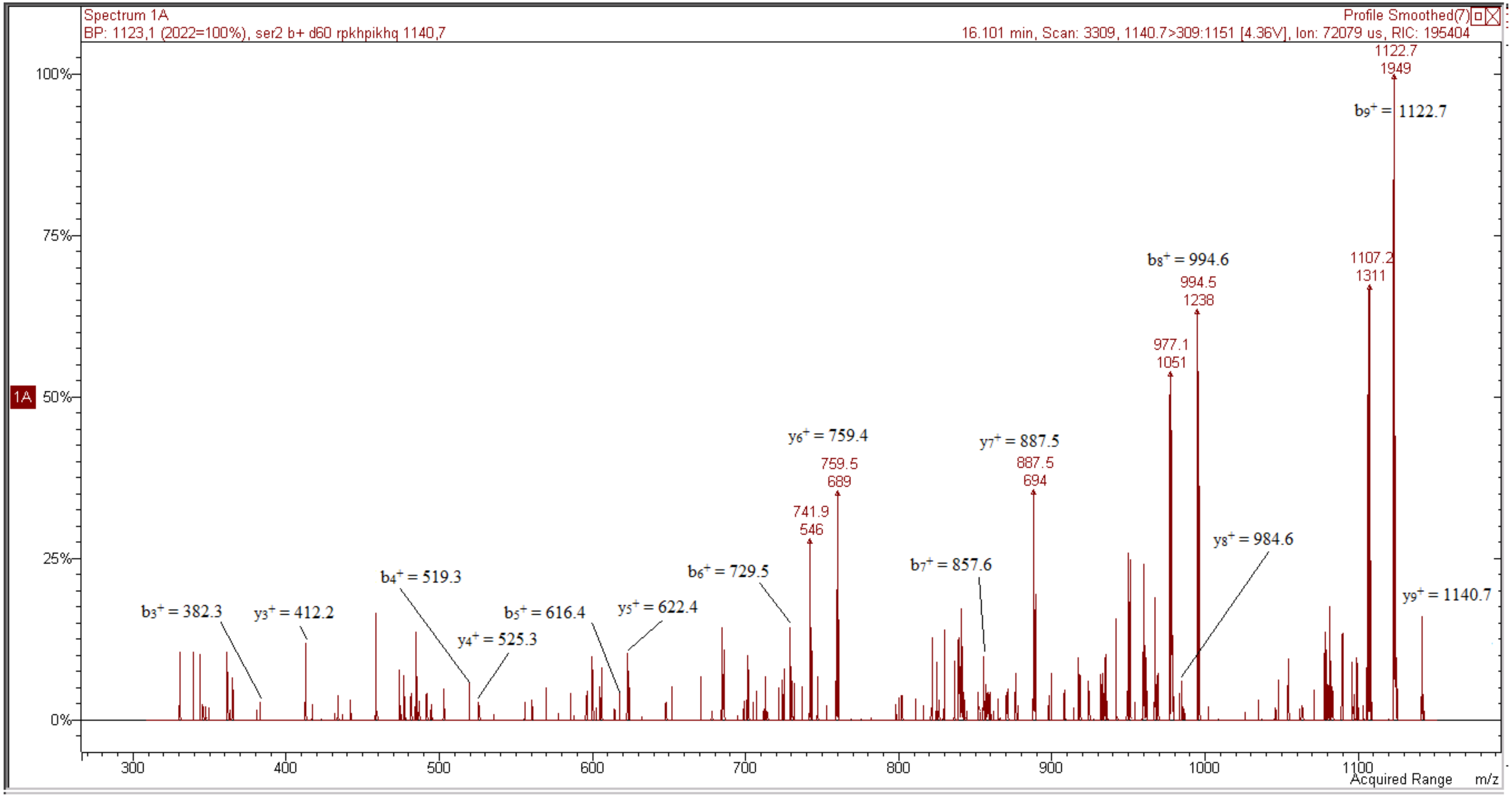

Figure S56. MS/MS spectrum of RPKHPIKHQ peptide.

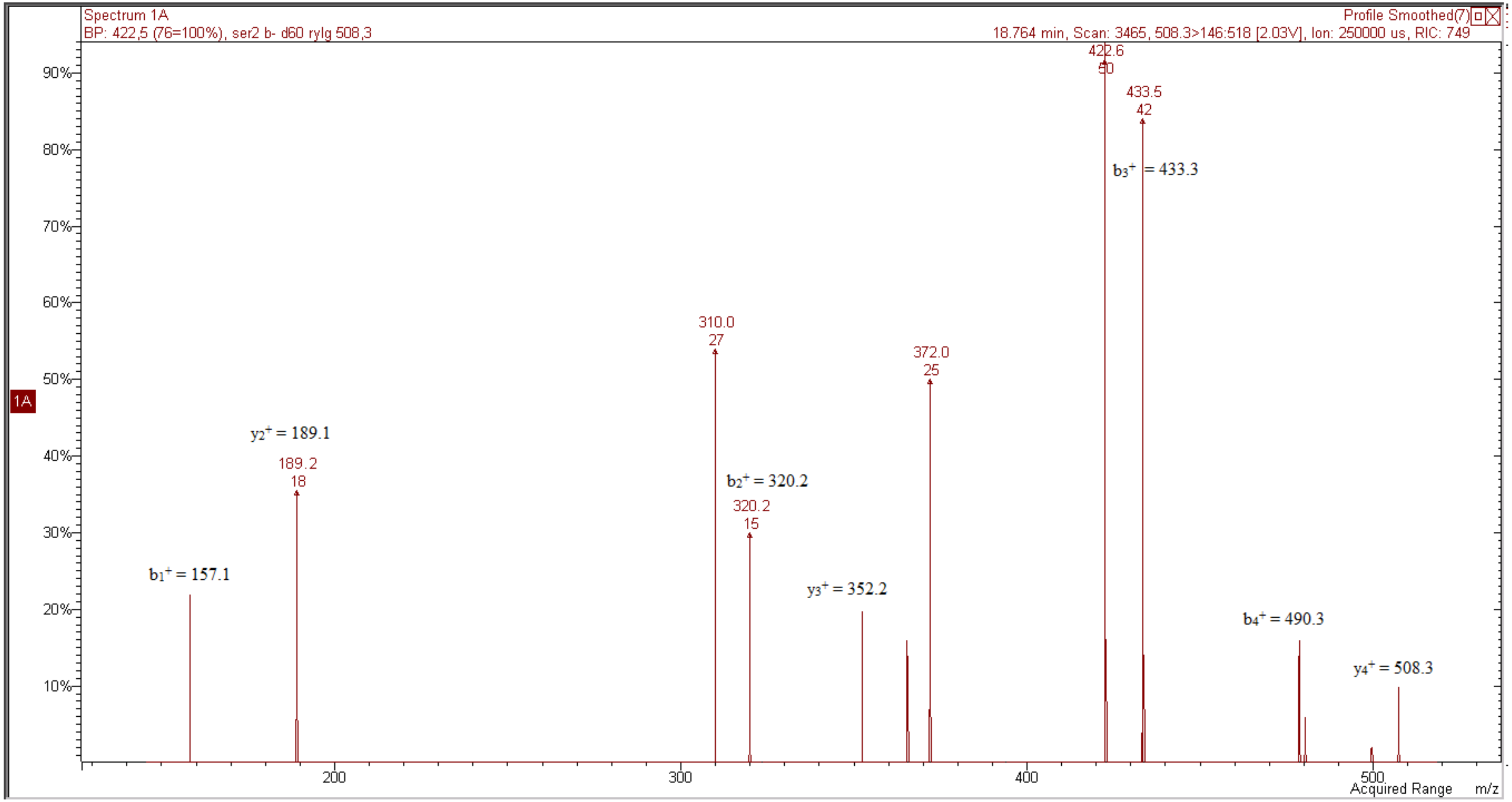

Figure S57. MS/MS spectrum of RYLG peptide.

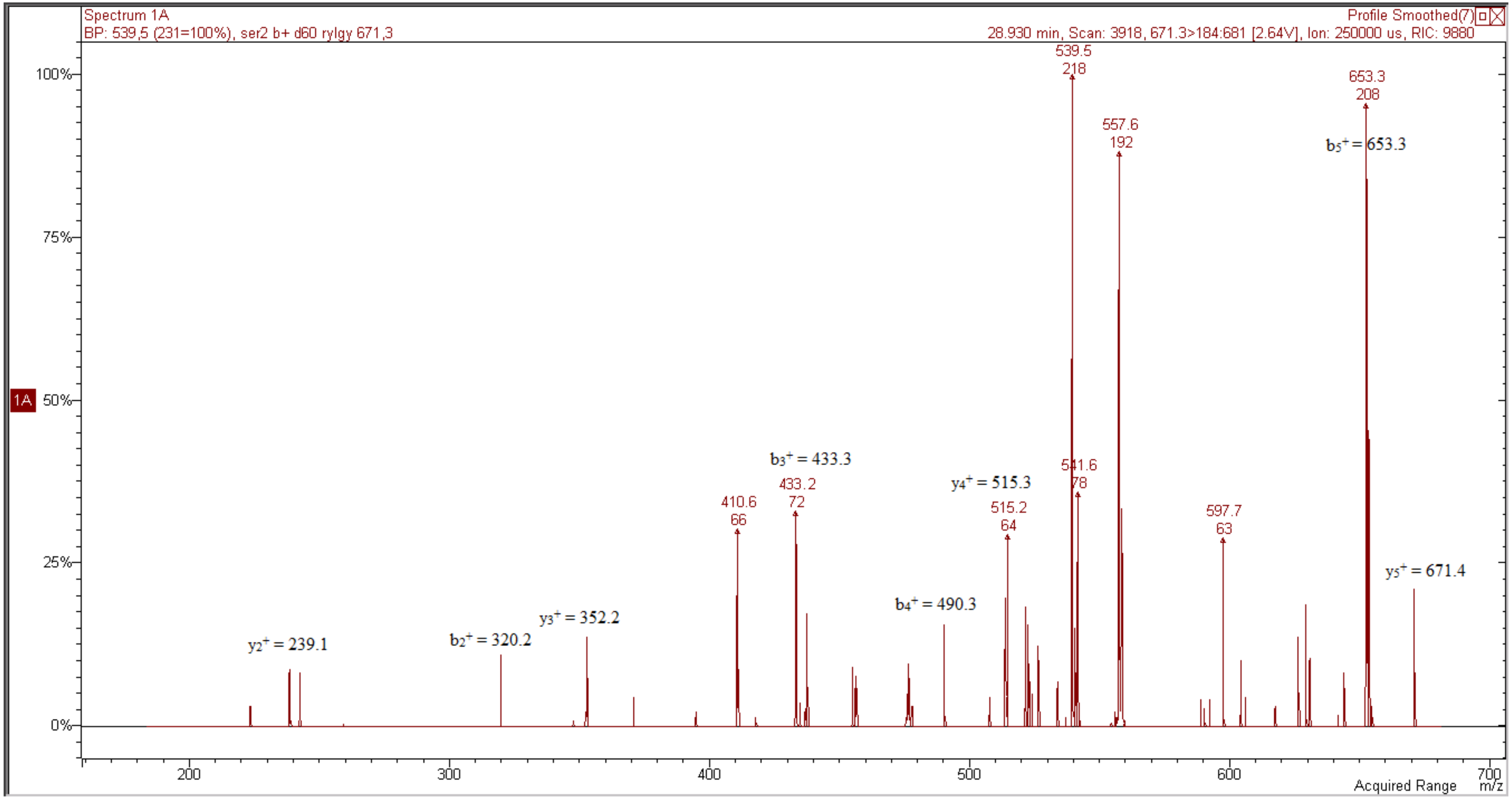

Figure S58. MS/MS spectrum of RYLGY peptide.

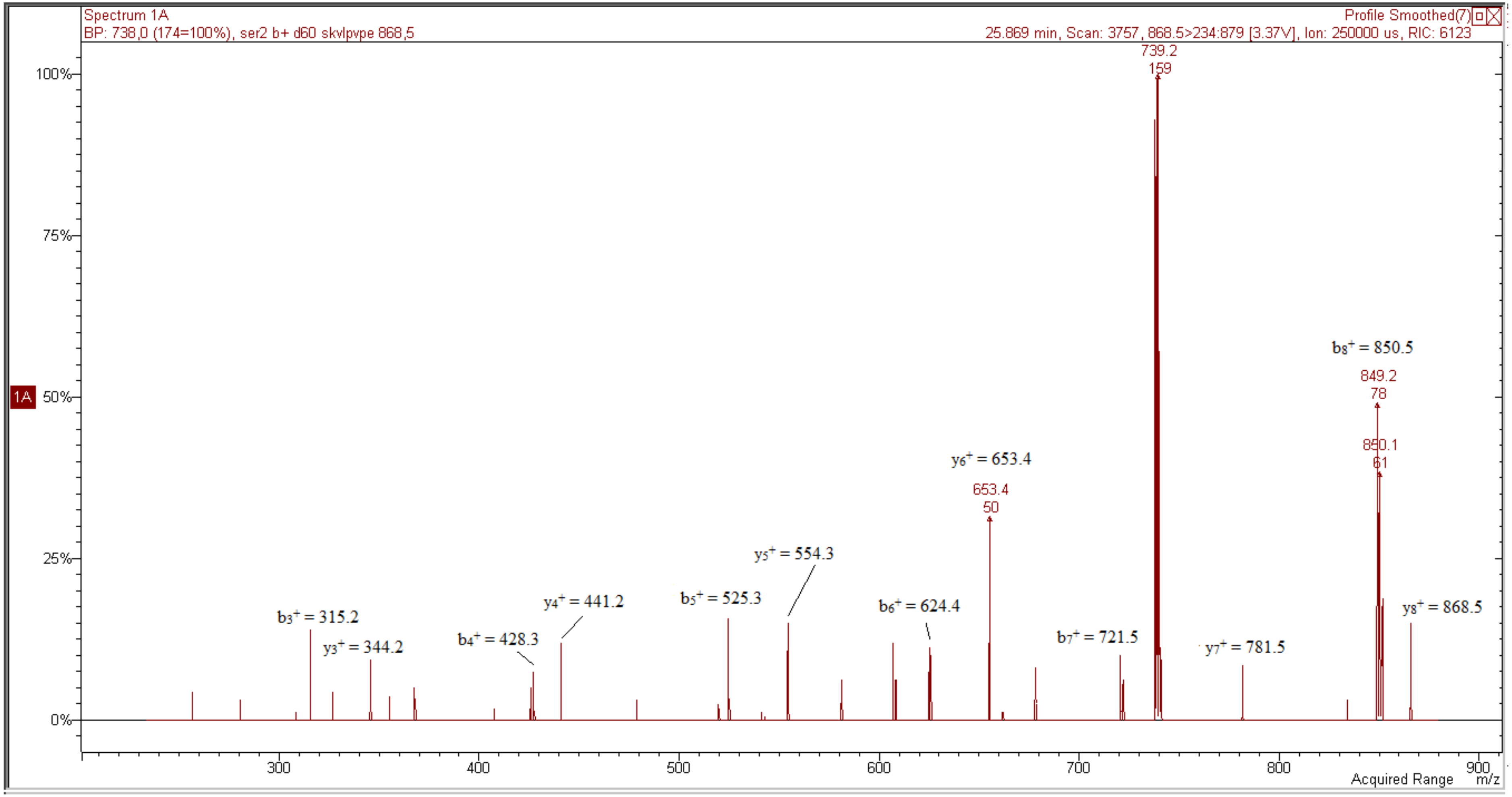

Figure S59. MS/MS spectrum of SKVLPVPE peptide.

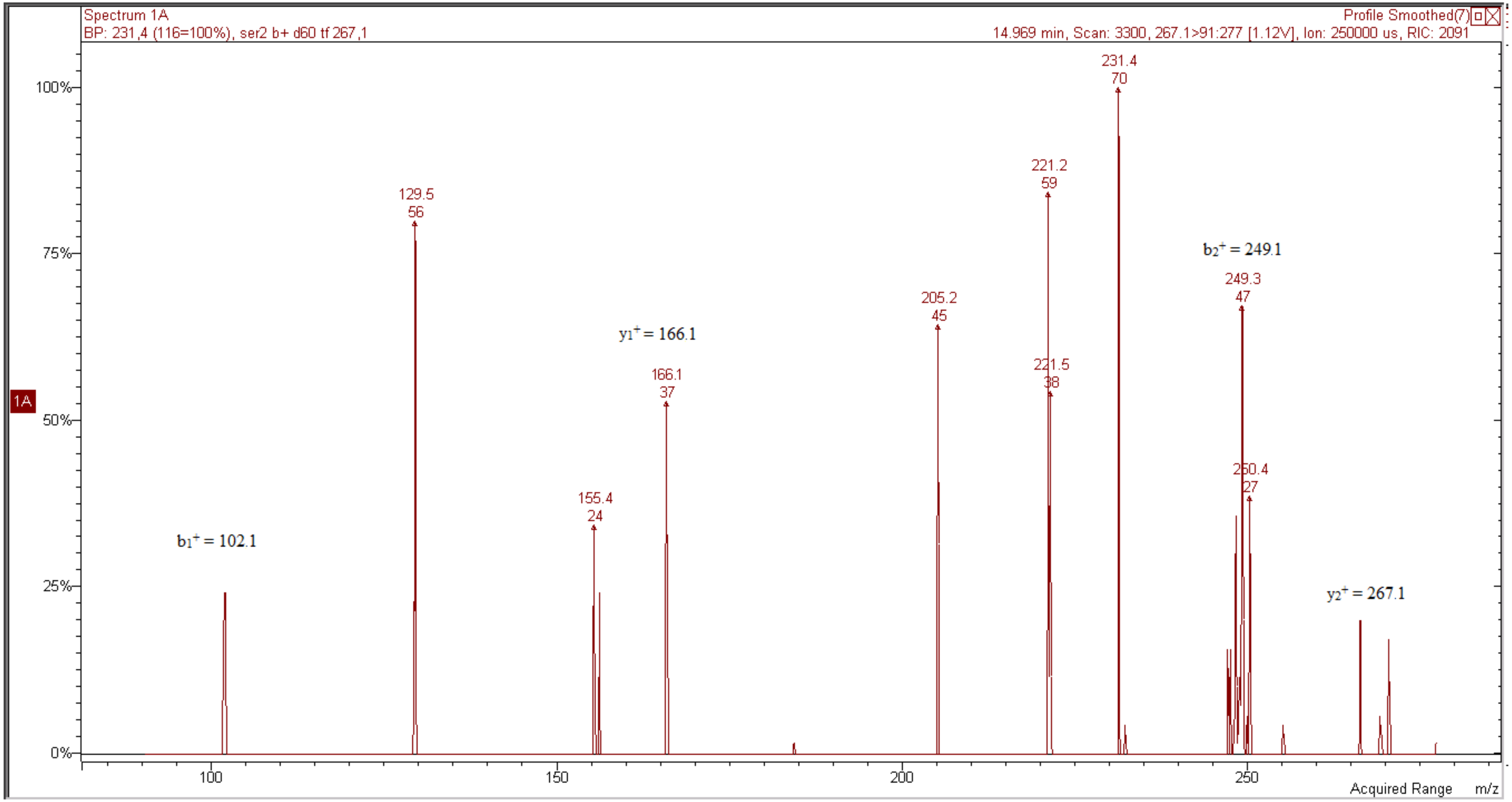

Figure S60. MS/MS spectrum of TF peptide.

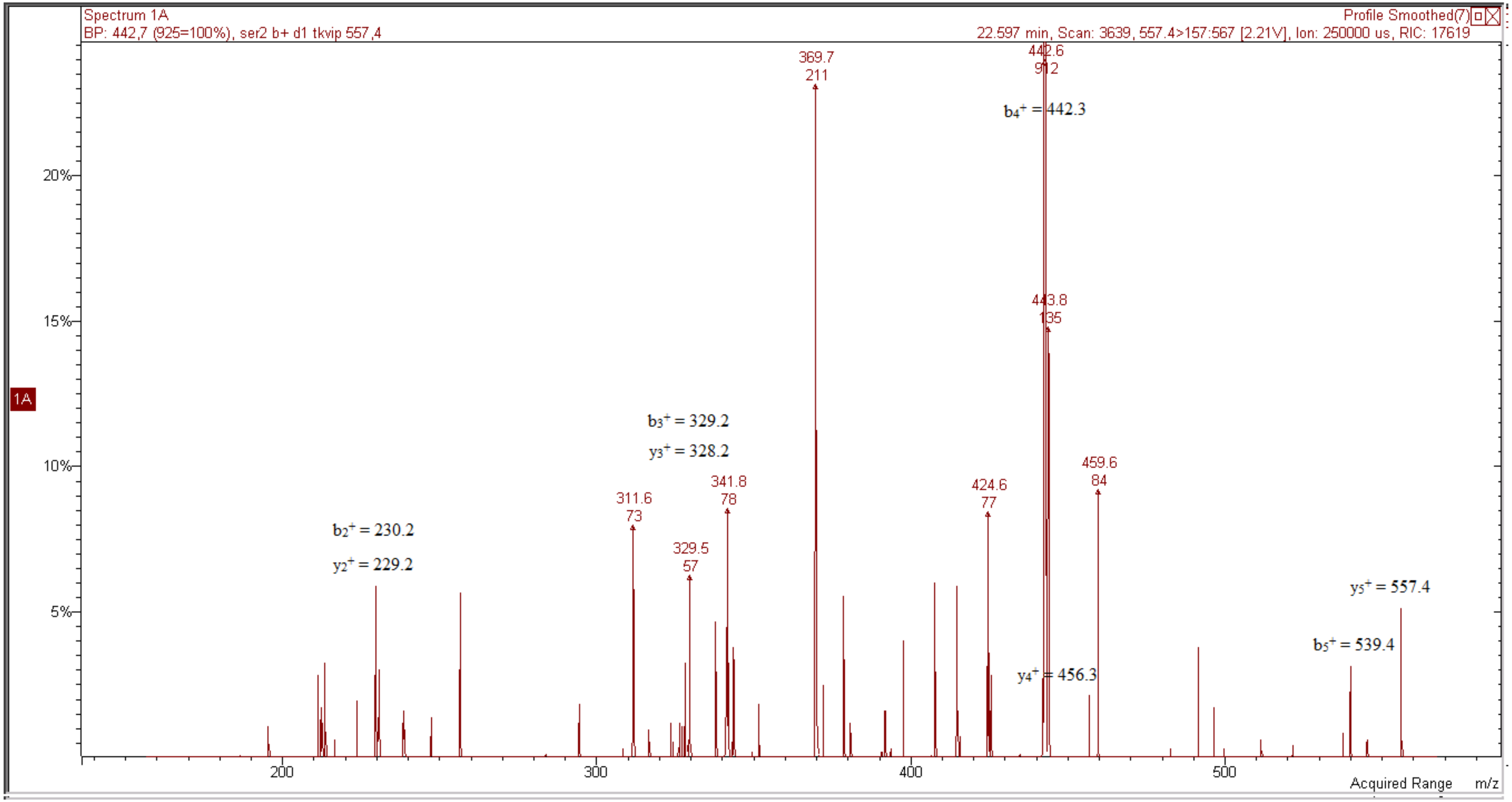

Figure S61. MS/MS spectrum of TKVIP peptide.

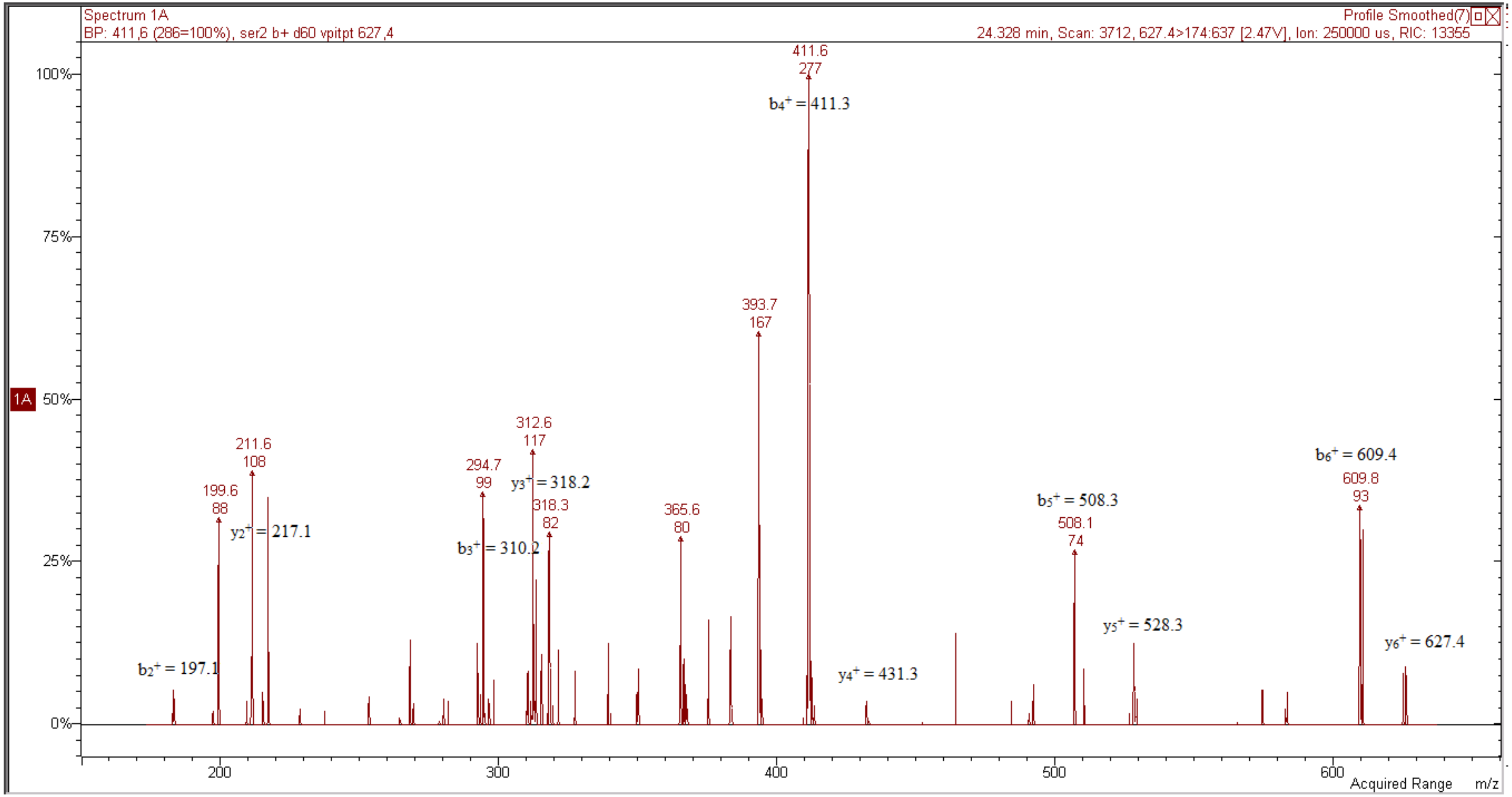

Figure S62. MS/MS spectrum of VPITPT peptide.

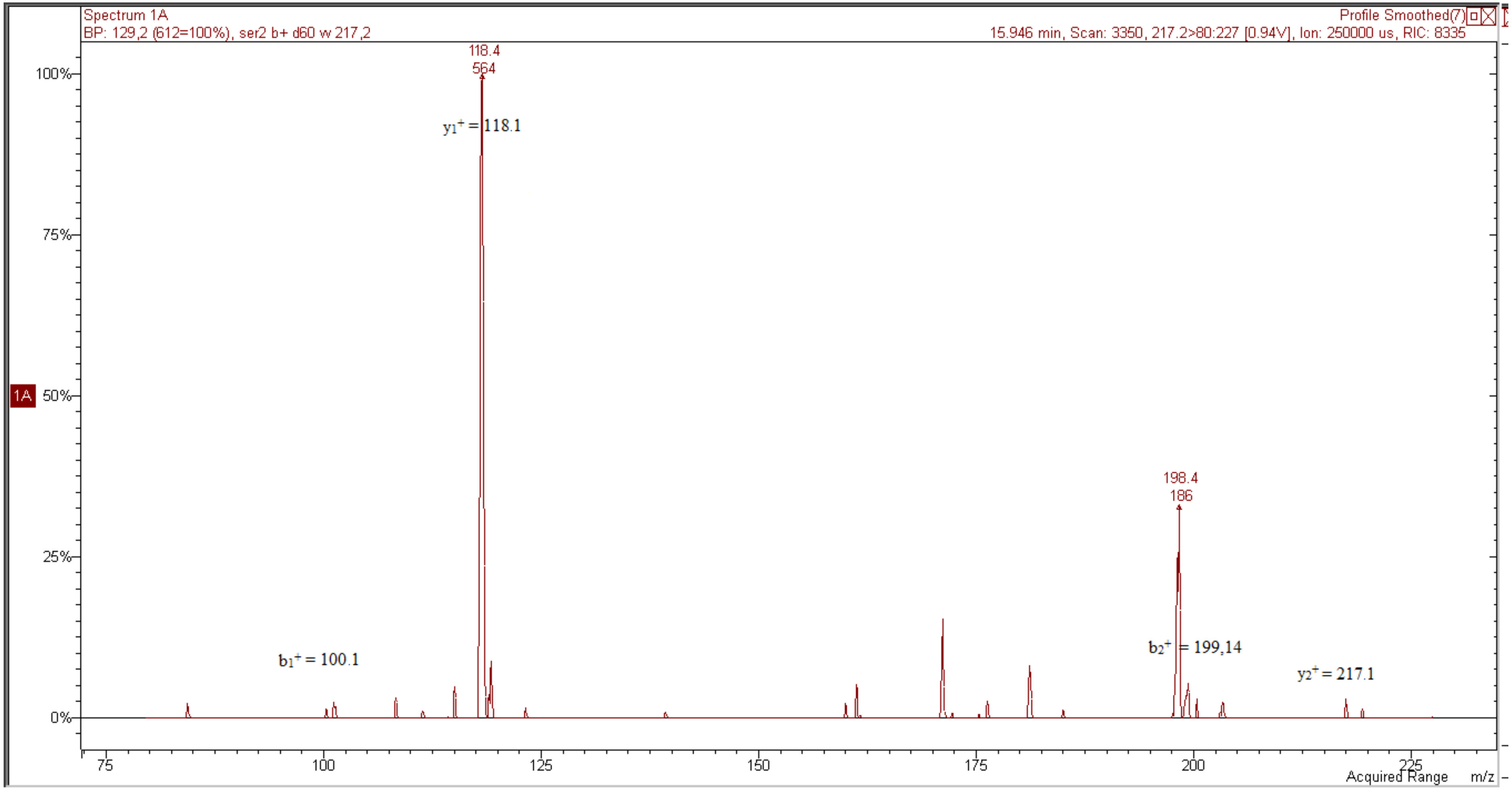

Figure S63. MS/MS spectrum of VV peptide.

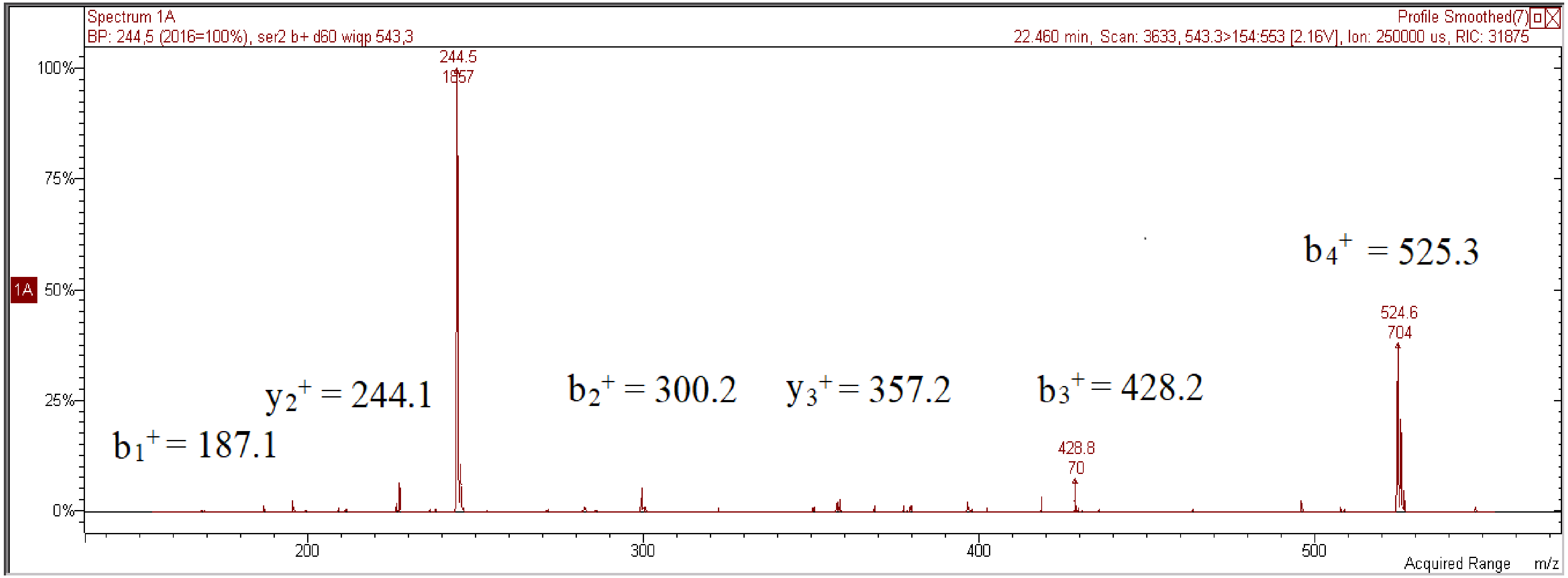

Figure S64. MS/MS spectrum of WIQP peptide.

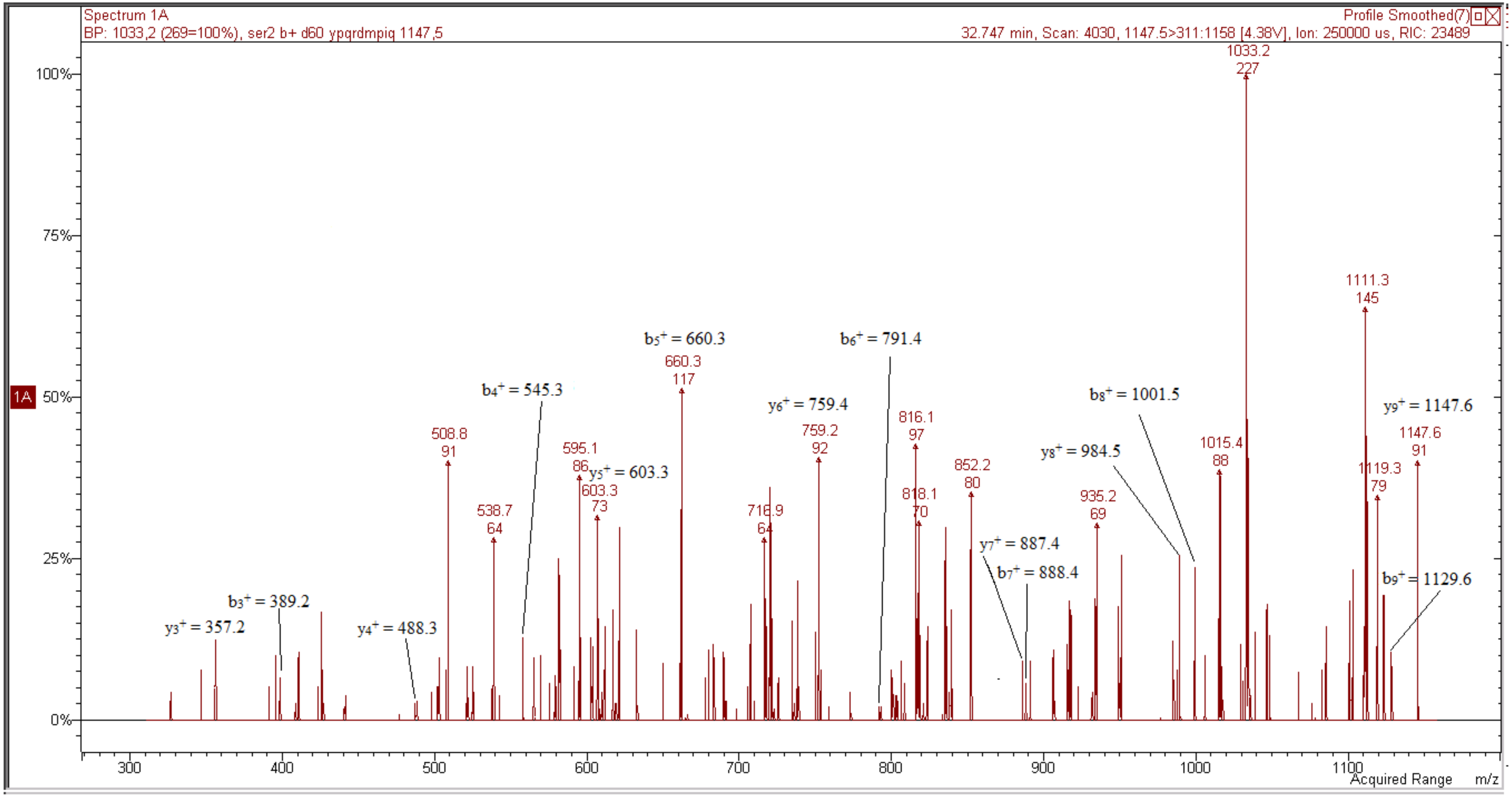

Figure S64. MS/MS spectrum of YPQRDMPIQ peptide.
